# Air-Stable, Highly Fluorescent Primary Phosphanes**

**DOI:** 10.1002/anie.201108416

**Published:** 2012-03-19

**Authors:** Laura H Davies, Beverly Stewart, Ross W Harrington, William Clegg, Lee J Higham

**Affiliations:** School of Chemistry, Bedson Building, Newcastle UniversityNewcastle upon Tyne, NE1 7RU (UK)

**Keywords:** fluorescence, phosphanes, rhenium, synthetic methods

Primary phosphanes (RPH_2_) have a reputation as noxious compounds which are spontaneously flammable in air.^[1]^ We have recently demonstrated, however, that they can be stabilized to air oxidation without any need for steric protection, providing sufficient π conjugation is incorporated into the organic R group; thus we were able to prepare the first air-stable chiral primary phosphanes.^[2]^ Since then, we have been developing a working model based on DFT, which indicates that, contrary to popular belief, many primary phosphanes will be air-stable if the molecule contains a high degree of conjugation (see below).^[3]^ As such, the model predicted that the incorporation of the phosphino group onto a boron dipyrromethene (Bodipy^[4]^) skeleton would also produce air-stable primary phosphanes. These phosphanes should provide a highly versatile gateway into a vast range of fluorescent phosphane derivatives, which are currently sorely underrepresented, despite the importance of phosphanes in catalytic and biomedical applications. To explore this exciting possibility, we commenced a synthetic study based on the strategy shown in Scheme 1. The fluorescent aryl bromide derivative of Bodipy, **1**, was synthesized in a one-pot reaction.^[5]^ Following difficulties with retaining the two fluorine atoms in the later reduction step,^[6]^ we treated **1** with two equivalents of phenyllithium to give the novel derivative **2 a**, which was characterized by X-ray crystallography (Figure S1 in the Supporting Information). A palladium-catalyzed coupling reaction of **2 a** with diethylphosphite yielded the fluorescent phosphonate **3 a**, which was also analyzed by X-ray crystallography (Figure S3 in the Supporting Information). Phosphonate **3 a** was then reduced quantitatively to the primary phosphane **4 a** by using a combination of lithium aluminum hydride and chlorotrimethylsilane. As predicted by the model, **4 a** was found to be stable to oxidation; when exposed to air both in the solid state and in chloroform solution over seven days, no decomposition was observed. Importantly, incorporation of the phosphonate and the phosphino group did not dramatically alter the photophysical properties of the molecules when compared to the parent aryl bromide **2 a** (Table 1). This finding was not unexpected, as the results from our DFT calculations show that the highest occupied molecular orbitals (HOMOs), up to the HOMO-6 of **2 a**, do not incorporate the phosphorus atom, although they are affected by the phenyl rings on the boron atom (Figure S6 in the Supporting Information). It is noteworthy that, after the aryl substitution of **1**, the quantum yield (*Φ*) of aryl bromide **2 a** drops from 0.65 to 0.079. In an effort to maintain the high quantum yield of **1**, but to still allow the reduction step, we treated **1** with two equivalents of methyllithium to give the dimethyl aryl bromide derivative **2 b**, which was also characterized by X-ray crystallography (Figure S2 in the Supporting Information). Substitution of the fluorine atoms by methyl groups has a less detrimental effect on the quantum yield (compare *Φ* values for **1**, **2 a**, and **2 b**, Table 1). We then adopted the previous methodology to prepare the corresponding phosphonate **3 b** and the primary phosphane **4 b**, which also retain generally better quantum yields and molar absorption coefficients (*Φ* and *ɛ* and Table 1).^[7]^ The primary phosphane **4 b** is air-stable as a solid and in solution over seven days; Figure 1 depicts its crystallographically determined molecular structure.

**Figure 1:**
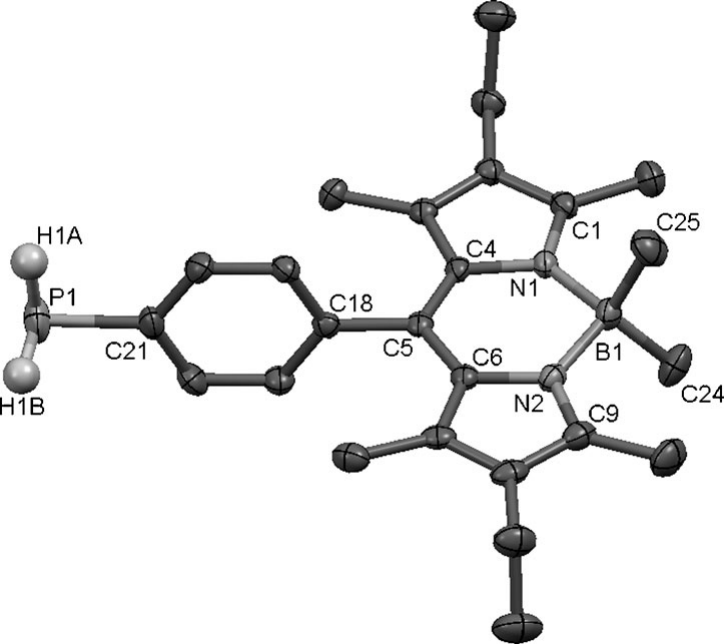
View of the molecular structure of 4 b with 50 % probability displacement ellipsoids. Hydrogen atoms bound to carbon atoms have been omitted for clarity. Selected bond distances [Å] and angles [°]: P1–H1A 1.42(4), P1–H1B 1.23(4), P1–C21 1.8289(17), C5–C18 1.493(2), C5–C6 1.397(2), N2–C6 1.400(2), B1–N1 1.591(2), B1–N2 1.593(2), B1–C24 1.615(3); H1A-P1-H1B 87(2), C4-C5-C6 122.60(15), N1-B1-C24 110.61(15), N1-B1-N2 104.89(12).

**Scheme 1:**
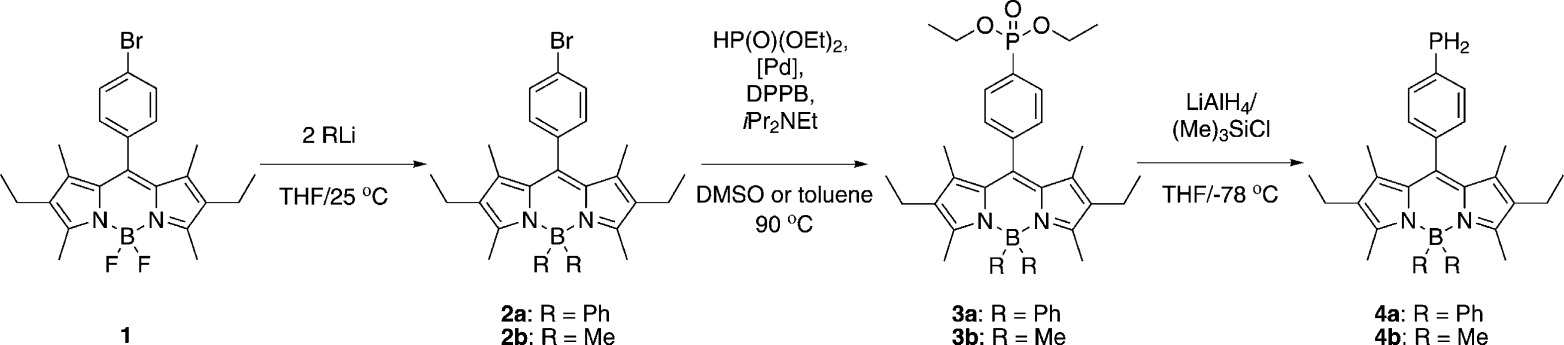
Synthetic procedure for the novel compounds 2 a 2 b, 3 a 3 b, and 4 a b. DPPB=1,4-bis(diphenylphosphino)butane, DMSO=dimethyl sulfoxide.

**Table 1:** Photochemical data of compounds 2 a/b, 3 a/b, and 4 a/b.[a]

	*λ*_abs_ [nm]	*λ*_em_ [nm]	*ɛ* [m^−1^ cm^−1^]	*Φ*
**1**	526	540	78 000	0.65
**2 a**	519	531	80 000	0.079
**2 b**	514	524	87 000	0.36
**3 a**	518	534	83 000	0.039
**3 b**	513	527	91 000	0.29
**4 a**	518	532	79 000	0.042
**4 b**	512	526	79 000	0.33
**5 b**	513	528	90 000	0.34
**6 b**	513	527	64 000	0.28

[a] Determined in THF at room temperature.

Again this air stability is in accord with that predicted by the model; the high degree of π conjugation raises the orbital energies of both the neutral molecule and the associated radical cation, which was found to correlate with a higher resistance to air oxidation.^[3]^ For **4 b**, the phosphino group is not incorporated in any orbital above HOMO-3 (Figure S7 in the Supporting Information). The two methyl groups on the boron of **4 b** are involved in the HOMO-1, but in contrast to the diphenyl substitution on **4 a**, the dimethyl substitution has a much lower impact on the quantum yield of **4 b** (see above, Table 1), which has a value almost eight times that of **4 a**. Figure 2 plots absorption coefficient *ɛ* and fluorescence intensity versus wavelength *λ* to illustrate the absorption and emission maxima of **4 a** and **4 b**.

**Figure 2:**
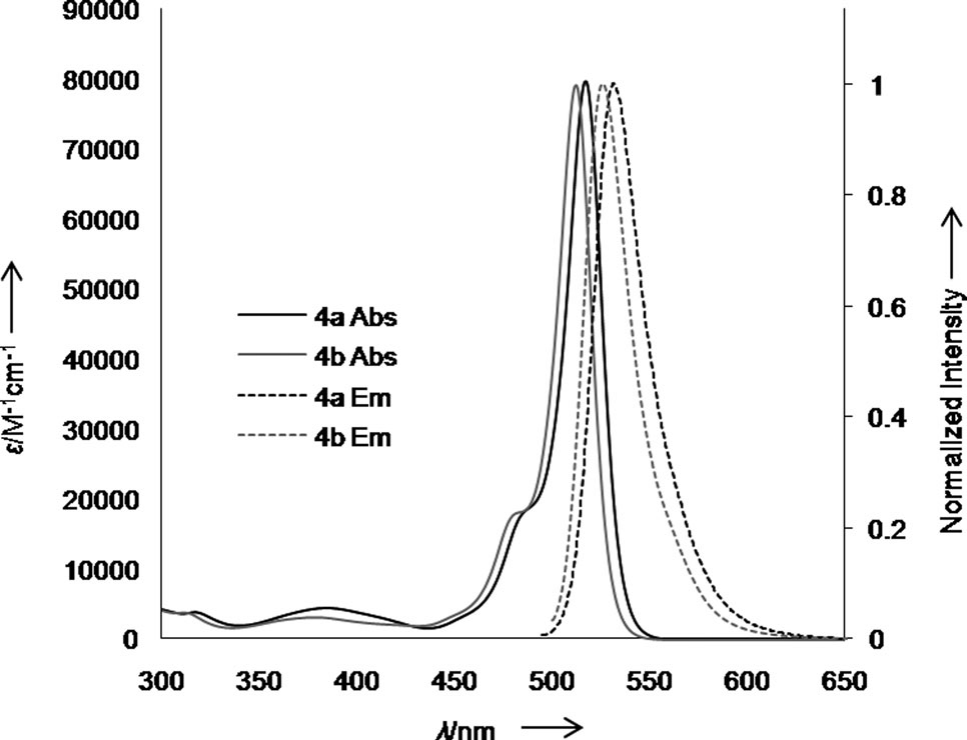
The absorption and emission photochemical profiles of 4 a and 4 b in THF; Stokes’ shifts of 14 nm are evident.

Having demonstrated that both **4 a** and **4 b** are air-stable and that **4 b** possesses desirable photophysical properties common to the Bodipy scaffold,[Bibr b4][Bibr b7] we next sought to measure the reactivity of the phosphino group, to establish if the resistance to air oxidation impinges on the behavior of the normally reactive P—H bonds. One of the classical reactions of a primary phosphane is the hydrophosphination reaction across a double bond. Zubieta, Valliant et al. have shown that fluorescent, tripodal, quinoline-derived complexes of rhenium and technetium have tremendous potential as radiopharmaceutical imaging agents.^[8]^ With this in mind, we treated **4 b** with two equivalents of vinyldiphenylphosphane, using [Pt(nbd)_3_] as catalyst (nbd=norbornadiene), to generate the tripodal ligand **5 b** in high yield. The ^31^P^{^1H} NMR spectrum in [D]chloroform gave a doublet at *δ*=−12.2 ppm and a triplet at *δ*=−16.4 ppm in a 2:1 ratio (^3^*J*_PP_=27.3 Hz). The phosphane also retains the photophysical characteristics of its precursor **4 b** (Table 1); modification at the phosphorus center is not detrimental. Having established that the air stability of **4 b** does not impede on the reactivity of its phosphino group, we sought to study some preliminary coordination chemistry of the tripodal phosphane **5 b**. Reaction of **5 b** with [ReCl(CO)_3_(PPh_3_)_2_] in mesitylene generated the octahedral rhenium complex **6 b** (Scheme 2).

**Scheme 2:**
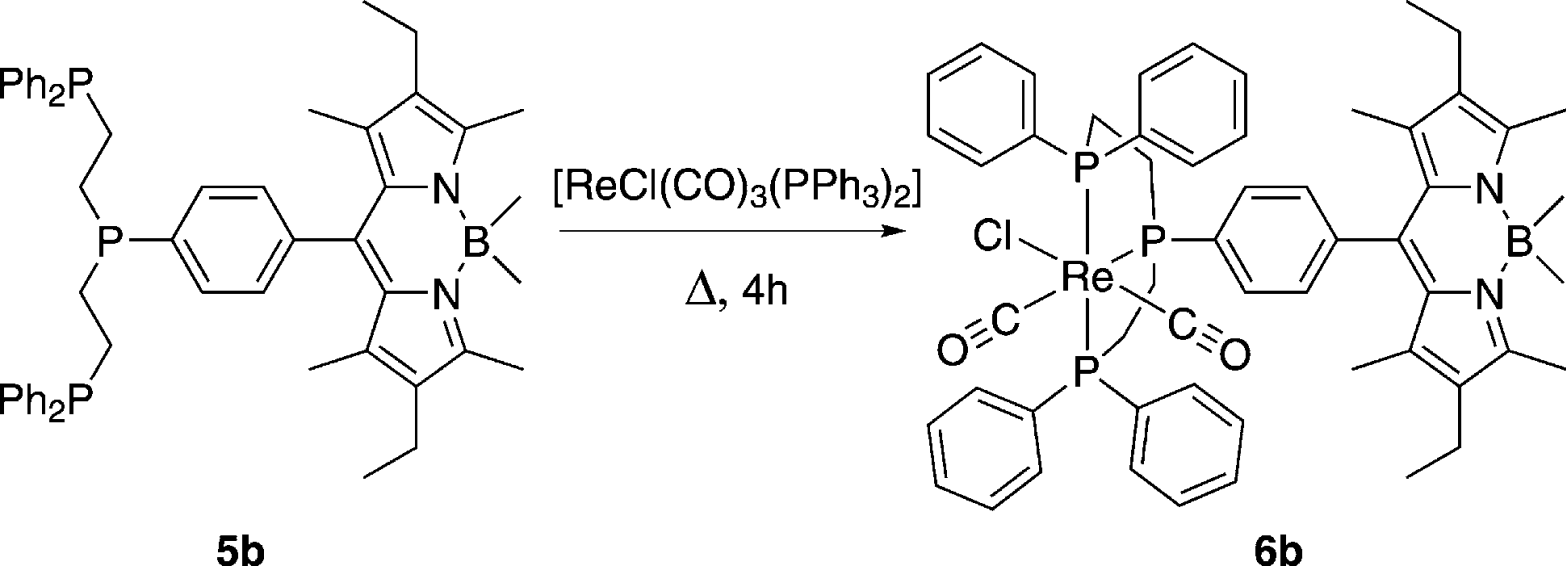
The synthesis of *cis*,*mer*-[ReCl(CO)_2_(5 b)].

A sample of **6 b** was analyzed by X-ray crystallography, and the molecular structure is depicted in Figure 3. This structure shows that the tripodal phosphane adopts a *mer* configuration about the rhenium center, with the central phosphorus atom *trans* to a carbonyl ligand and the terminal phosphorus atoms of the ligand **5 b** *trans* to each other. Of particular note is the elongated metal–carbon bond length of the carbonyl ligand *trans* to the phosphorus atom, relative to that of the carbonyl ligand *trans* to the chlorine atom (1.943(5) versus 1.904(6) Å) and the wider bond angle present for the rhenium–phosphorus–Bodipy carbon junction (122.08(18)°) when compared to the corresponding angles when the carbon atoms form part of a metallocyclic ring (110.61(18) and 109.18(19)°). The parameters compare well with the only other known tripodal phosphane rhenium complexes, [ReCl(CO)_2_(triphos)] (triphos=bis(2-diphenylphosphanoethyl)phenylphosphane).^[9]^ The complex **6 b** retains a similar absorption–emission profile (*λ*_abs_ 513 nm; *λ*_em_ 527 nm) to that of the uncomplexed tripodal phosphane **5 b** and, although the molar absorption coefficient and quantum yield are lowered relative to **5 b**, these values are not dramatically affected; thus the molar absorption coefficient *ɛ* is lowered from 90 000 to 64 000 m^−1^ cm^−1^, and the quantum yield is reduced from 0.34 to 0.28. The high quantum yield value is of significance; the aforementioned rhenium tripodal nitrogen complexes give quantum yields of 0.015 to 0.003 (depending on the solvent), as do many other transition-metal fluorescence probes.^[10]^ Therefore the results indicate that the rhenium phosphane core is straightforward to prepare and retains a highly desirable photophysical profile. Because of the similar coordination chemistry, rhenium is a frequently used mimic of ^99m^Tc, which is the most widely used radionuclide in medicinal diagnoses.^[8,^^11]^ Thus cores such as **6 b** offer the potential for correlating fluorescence studies with radioimaging data to better understand and improve the imaging and targeting of diseases. Fluorescence microscopy would facilitate an understanding of cellular activity in vitro, which could then be used in conjunction with information garnered from living specimens that had been subjected to nuclear imaging techniques after treatment with the gamma-emitting ^99m^Tc analogues. Our studies are now focused in this area.

**Figure 3:**
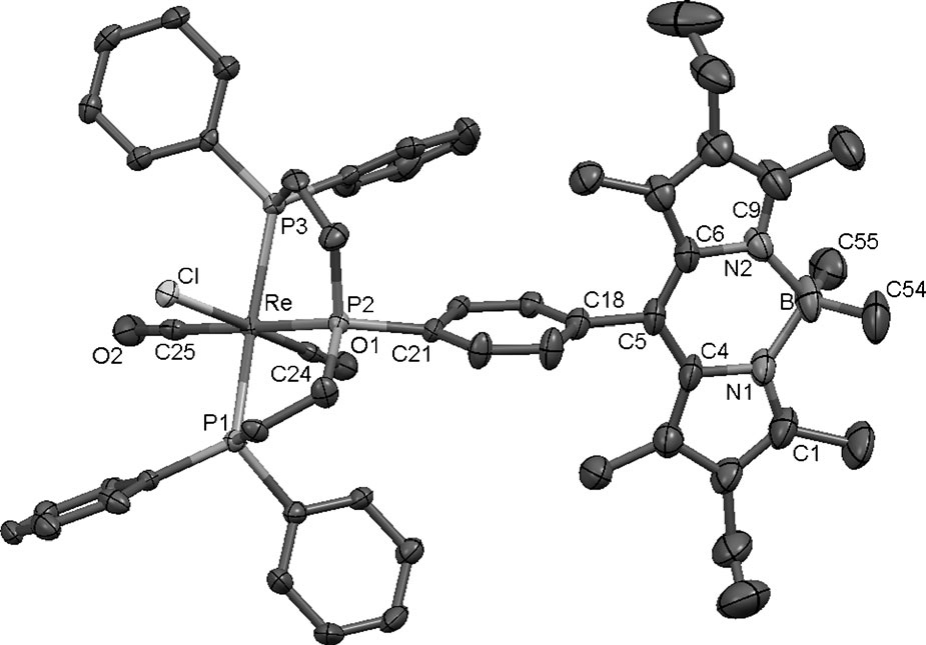
View of the molecular structure of 6 b with 50 % probability displacement ellipsoids. Hydrogen atoms have been omitted for clarity. Selected bond distances [Å] and angles [°]: Re–P1 2.3890(14), Re–P2 2.4068(13), Re–P3 2.3877(13), Re–C24 1.904(6), C24–O1 1.128(6), Re–C25 1.943(5), C25–O2 1.138(6), Re–Cl 2.5220(13), P2–C21 1.827(5), C5–C6 1.379(9), C6–N2 1.402(6), N2–B 1.591(9), B–C54 1.630(13); Cl-Re-P1 81.39(5), Cl-Re-P2 84.53(4), P1-Re-P3 155.18(5), C24-Re-C25 86.3(2), Re-C25-O2 178.0(5), Re-P2-C21 122.08(18), C4-C5-C6 122.5(5), N1-B-N2 105.5(5).^[12]^

## Experimental Section

Apart from the air stability studies, reactions were carried out using standard Schlenk-line techniques in anhydrous solvents. Full characterization data for compounds **1**–**6** are given in the Supporting Information.

**2 a**: Phenyllithium (4.57 mL, 9.15 mmol, 2.0 m solution in dibutyl ether) was added dropwise to a tetrahydrofuran solution (100 mL) of **1** (2.00 g, 4.36 mmol). The solution was stirred at room temperature until complete consumption of the starting material was observed by TLC. The reaction was quenched with water (20 mL), extracted with dichloromethane (2×100 mL), and the combined organic fractions were washed with water (40 mL) and brine (40 mL) and dried over magnesium sulfate. Column chromatography on silica (petroleum ether/toluene, 4:1) gave an orange solid (1.10 g, 44 %).

**2 b**: The compound was synthesized in a similar fashion to the above procedure from **1** (2.00 g, 4.36 mmol) and methyllithium (5.72 mL, 9.15 mmol, 1.6 m solution in diethyl ether). Column chromatography on silica (petroleum ether/toluene, 9:1) gave an orange solid (0.79 g, 40 %).

**3 a**: Compound **2 a** (1.00 g, 1.74 mmol) was dissolved in dimethyl sulfoxide (40 mL), and palladium acetate (0.117 g, 0.17 mmol), diethyl phosphite (0.25 mL, 1.91 mmol), *N*,*N*-diisopropylethylamine (0.91 mL, 5.21 mmol), and 1,4-bis(diphenylphosphino)butane (0.074 g, 0.17 mmol) were added. The mixture was degassed for 20 min, before being heated at 90 °C for three days. The reaction was diluted with dichloromethane (100 mL) and washed with water (40 mL) and brine (40 mL) and dried over magnesium sulfate. Column chromatography on silica (ethyl acetate/petroleum ether, 2:1) gave an orange solid (0.96 g, 87 %).

**3 b**: The compound was prepared in a similar fashion to **3 a**, by using **2 b** (1.00 g, 2.22 mmol), toluene (40 mL), bis(dibenzylideneacetone)palladium (0.128 g, 0.22 mmol), diethyl phosphite (0.34 mL, 2.66 mmol), *N*,*N*-diisopropylethylamine (1.16 mL, 6.66 mmol), and 1,4-bis(diphenylphosphino)butane (0.095 g, 0.22 mmol). Column chromatography on silica (ethyl acetate/*n*-hexane, 3:1) gave an orange solid (0.60 g, 53 %).

**4 a**: Lithium aluminum hydride (3.16 mL, 3.16 mmol, 1.0 m solution in tetrahydrofuran) was added to a Schlenk flask and cooled to −78 °C in a dry ice/acetone bath. Chlorotrimethylsilane (0.40 mL, 3.16 mmol) was added to the resultant white suspension, and the mixture was allowed to warm to room temperature over 30 min. A colorless solution was evident which was then cooled to −40 °C in a dry ice/acetonitrile bath, and a solution of **3 a** (1.00 g, 1.58 mmol) in tetrahydrofuran (100 mL) was added dropwise. The reaction was allowed to warm to room temperature and stirred overnight. The solution was concentrated in vacuo and then quenched in an ice-bath with degassed water (20 mL). The product was extracted with diethylether (3×20 mL) and dried over magnesium sulfate. Column chromatography on silica (dichloromethane/petroleum ether, 1:2) gave **4 a** as an orange solid (0.77 g, 92 %).

**4 b**: The compound was prepared in a similar fashion to **4 a**, by using lithium aluminum hydride (3.93 mL, 3.93 mmol, 1.0 m solution in tetrahydrofuran), chlorotrimethylsilane (0.50 mL, 3.93 mmol), and **3 b** (1.00 g, 1.97 mmol) in tetrahydrofuran (100 mL). Column chromatography on silica (chloroform/*n*-hexane, 1:4) gave **4 b** as an orange solid (0.74 g, 93 %).^[12]^

**5 b**: Vinyldiphenylphosphane (0.16 mL, 0.78 mmol), [Pt(nbd)_3_] (0.018 g, 0.037 mmol), and **4 b** (0.150 g, 0.37 mmol) were dissolved in toluene (10 mL), and the reaction was stirred at reflux for five days. Column chromatography on silica (dichloromethane/*n*-hexane, 1:1) gave **5 b** as an orange solid (0.21 g, 70 %).

**6 b**: **5 b** (0.050 g, 0.060 mmol), [ReCl(CO)_3_(PPh_3_)_2_] (0.050 g, 0.060 mmol), and mesitylene (2 mL) were heated to reflux for four hours. After passing the mixture through a silica pad, eluting first with *n*-hexane and then with dichloromethane, an orange solid was obtained (0.051 g, 82 %).^[12]^

## Notes

Detailed facts of importance to specialist readers are published as ”Supporting Information”. Such documents are peer-reviewed, but not copy-edited or typeset. They are made available as submitted by the authors.
