# Supplementary material for: Air-Stable, Highly Fluorescent Primary Phosphanes
Source: Angew Chem Int Ed Engl. 2012 Mar 19;51(20):4921–4. doi: 10.1002/anie.201108416 (PMC3531623; doi:10.1002/anie.201108416)
Supplement: Supplementary file 1 [file anie0051-4921-sd1.pdf]

Angewandte Chemie
Eine Zeitschrift der Gesellschaft Deutscher Chemiker

Supporting Information

© Wiley-VCH 2012

69451 Weinheim, Germany

Air-Stable, Highly Fluorescent Primary Phosphanes**

*Laura H. Davies, Beverly Stewart, Ross W. Harrington, William Clegg, and Lee J. Higham**

anie_201108416_sm_miscellaneous_information.pdf

Supporting Information

Table of Contents

Table of Contents 1

1 Experimental Procedures 2

1.1 General considerations 2

1.2 8-(4-bromophenyl)-4,4-diphenyl-1,3,5,7-tetramethyl-2,6-diethyl-4-bora-3a,4a-diaza-*s*-indacene (**2a**) 3

1.3 8-(4-bromophenyl)-4,4-dimethyl-1,3,5,7-tetramethyl-2,6-diethyl-4-bora-3a,4a-diaza-*s*-indacene (**2b**) 4

1.4 8-((4-diethylphosphonato)phenyl)-4,4-diphenyl-1,3,5,7-tetramethyl-2,6-diethyl-4-bora-3a,4a-diaza-*s*-indacene (**3a**) 5

1.5 8-((4-diethylphosphonato)phenyl)-4,4-dimethyl-1,3,5,7-tetramethyl-2,6-diethyl-4-bora-3a,4a-diaza-*s*-indacene (**3b**) 6

1.6 8-((4-phosphino)phenyl)-4,4-diphenyl-1,3,5,7-tetramethyl-2,6-diethyl-4-bora-3a,4a-diaza-*s*-indacene (**4a**) 7

1.7 8-((4-phosphino)phenyl)-4,4-dimethyl-1,3,5,7-tetramethyl-2,6-diethyl-4-bora-3a,4a-diaza-*s*-indacene (**4b**) 8

1.8 8-(4-(bis-2-ethyldiphenylphosphino)-phenylphosphane)-4,4-dimethyl-1,3,5,7-tetramethyl-2,6-diethyl-4-bora-3a,4a-diaza-*s*-indacene (**5b**) 9

1.9 *cis*,*mer*-[ReCl(CO)$_2$(**5b**)] (**6b**) 10

2 Primary Phosphane Air Stability Experiments 11

2.1 Solid-state experiments 11

2.2 Solution-state experiments 11

3 X-ray Crystallography 12

3.1 General information 12

3.2 Crystallographic data for **2a** 13

3.3 Crystallographic data for **2b** 27

3.4 Crystallographic data for **3a** 35

3.5 Crystallographic data for **4b** 44

3.6 Crystallographic data for **6b** 52

4 Quantum Chemical Calculations 63

4.1 General considerations 63

4.2 Molecular orbital surfaces 63

5 Absorption and Emission Spectroscopy 64

5.1 General considerations 64
5.2 Absorption and emission spectra 64
6 References 65

1 Experimental Procedures

1.1 General considerations

All air- and/or water-sensitive reactions were performed under a nitrogen atmosphere using standard Schlenk line techniques. Tetrahydrofuran, toluene and mesitylene were dried over sodium; solvents were distilled prior to use. DMSO (Acros) was purchased in an anhydrous state. All starting materials were purchased from either Aldrich, Acros Organics or Alfa Aesar and used as received. 8-(4-bromophenyl)-4,4-difluoro-1,3,5,7-tetramethyl-2,6-diethyl-4-bora-3a,4a-diaza-*s*-indacene, 8-phenyl-4,4-difluoro-1,3,5,7-tetramethyl-2,6-diethyl-4-bora-3a,4a-diaza-*s*-indacene, and $[ReCl(CO)_3(PPh_3)_2]$ were prepared according to literature procedures.[1,2,3] Flash chromatography was performed on silica gel from Fluorochem (silica gel, 40-63u, 60A, LC301). Thin-layer chromatography was performed on Merck aluminum-based plates with silica gel and fluorescent indicator (254 nm). Melting points were determined in open glass capillary tubes on a Stuart SMP3 melting point apparatus. ^{1}H, $^{13}C\{^{1}H\}$, $^{31}P\{^{1}H\}$, and $^{11}B\{^{1}H\}$ NMR spectra were recorded on a JEOL Lambda 500 (^{1}H 500.16 MHz) or JEOL ECS-400 (^{1}H 399.78 MHz) spectrometer at room temperature (21°C) using the indicated solvent as internal reference; ^{1}H and ^{13}C shifts were relative to tetramethylsilane, ^{31}P relative to 80% H_3PO_4 and ^{11}B referenced relative to $BF_3.Et_2O$. Infrared spectra were recorded on a Varian 800 FT-IR spectrometer. Mass spectrometry was carried out by the EPSRC National Mass Spectrometry Service Centre, Swansea.

1.2 8-(4-bromophenyl)-4,4-diphenyl-1,3,5,7-tetramethyl-2,6-diethyl-4-bora-3a,4a-diaza-*s*-indacene (**2a**)

8-(4-bromophenyl)-4,4-difluoro-1,3,5,7-tetramethyl-2,6-diethyl-4-bora-3a,4a-diaza-*s*-indacene (2.00 g, 4.36 mmol) was dissolved in anhydrous tetrahydrofuran (100 mL). To this solution phenyllithium (4.57 mL, 9.15 mmol, 2.0M solution in dibutyl ether) was added dropwise, which caused the solution to turn darker with an orange tint; the solution was stirred at room temperature until complete consumption of the starting material was observed by TLC (~15 min). The reaction was quenched with water (20 mL) and the solution was extracted with dichloromethane (2 x 100 mL); the combined organic fractions were washed with water (40 mL) and brine (40 mL), and dried over magnesium sulfate. Purification was performed by column chromatography on silica gel (petroleum ether/toluene 4:1, R_f = 0.6) to yield an orange solid (1.10 g, 44%). A sample suitable for X-ray crystallographic analysis was obtained from dichloromethane. **^{1}H NMR** (400 MHz, $CDCl_3$) δ 7.60 (d, $^{3}J_{HH}$ = 8.2 Hz, 2H), 7.36 (d, $^{3}J_{HH}$ = 8.2 Hz, 2H), 7.14-7.24 (m, 10H), 2.19 (q, $^{3}J_{HH}$ = 7.3 Hz, 4H), 1.74 (s, 6H), 1.32 (s, 6H), 0.88 (t, $^{3}J_{HH}$ = 7.3 Hz, 6H) ppm; **^{13}C{^{1}H} NMR** (100 MHz, $CDCl_3$) δ 153.4, 150.2, 138.9, 135.9, 135.0, 133.8, 133.1, 132.0, 130.7, 130.5, 127.1, 125.5, 122.6, 17.3, 14.7, 14.6, 12.3 ppm; **^{11}B{^{1}H} NMR** (128 MHz, $CDCl_3$) δ -1.2 ppm; **IR** (neat): $\tilde{\nu}$ = 2960 (w), 1548 (s), 1472 (m), 1385 (s), 1303 (w), 1169 (s), 1140 (m), 1111 (m), 968 (s), 772 (s) cm^{-1}; **MP**: 201-204°C; **HRMS** (ESI^{+}) calc. $C_{35}H_{37}B_1N_2Br_1$ for $[M+H]^{+}$ requires m/z 574.2264, found *m*/*z* 574.2262 (0.3 ppm).

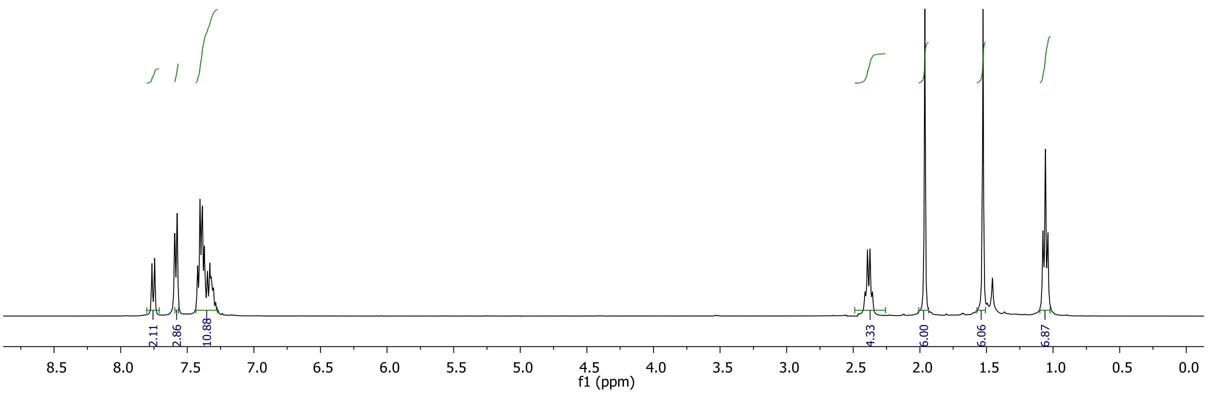

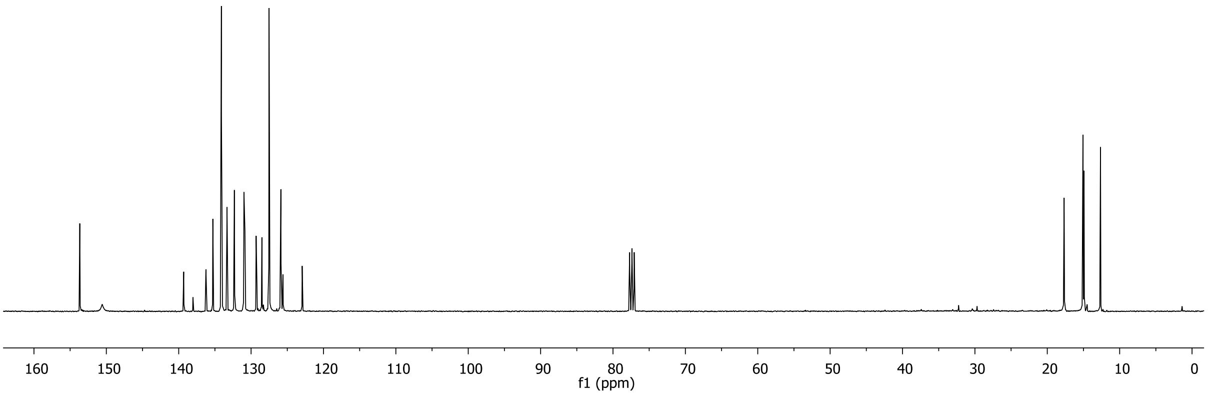

1.3 8-(4-bromophenyl)-4,4-dimethyl-1,3,5,7-tetramethyl-2,6-diethyl-4-bora-3a,4a-diaza-*s*-indacene (**2b**)

Prepared in the same manner as **2a**, using 2.00 g (4.36 mmol) of 8-(4-bromophenyl)-4,4-difluoro-1,3,5,7-tetramethyl-2,6-diethyl-4-bora-3a,4a-diaza-*s*-indacene and 5.72 mL (9.15 mmol) of methyllithium (1.6M solution in diethyl ether). Purification was performed by column chromatography on silica gel (petroleum ether/toluene 9:1, R_f = 0.5) to yield an orange solid (0.79 g, 40%). A sample suitable for X-ray crystallographic analysis was obtained from dichloromethane. **^{1}H NMR** (400 MHz, $CDCl_3$) δ 7.60 (d, $^3J_{HH}$ = 7.8 Hz, 2H), 7.19 (d, $^3J_{HH}$ = 7.8 Hz, 2H), 2.44 (s, 6H), 2.31 (q, $^3J_{HH}$ = 7.3 Hz, 4H), 1.23 (s, 6H), 0.91 (t, $^3J_{HH}$ = 7.3 Hz, 6H), 0.20 (s, 6H) ppm; **^{13}C{^{1}H} NMR** (100 MHz, $CDCl_3$) δ 150.9, 138.8, 136.3, 133.6, 132.6, 132.0, 130.6, 128.8, 122.4, 17.4, 14.7, 14.3, 12.0, 10.4 ppm; **^{11}B{^{1}H} NMR** (128 MHz, $CDCl_3$) δ -2.0 ppm; **IR** (neat): $\tilde{\nu}$ = 2925 (w), 1546 (s), 1470 (m), 1358 (m), 1314 (m), 1166 (s), 1142 (m), 1109 (m), 1060 (m), 940 (s), 796 (s) cm^{-1}; **MP**: 220-224°C; **HRMS** (ESI$^+$) calc. for $C_{25}H_{33}B_1N_2Br_1$ $[M+H]^+$ requires m/z 450.1951, found *m/z* 450.1952 (0.2 ppm).

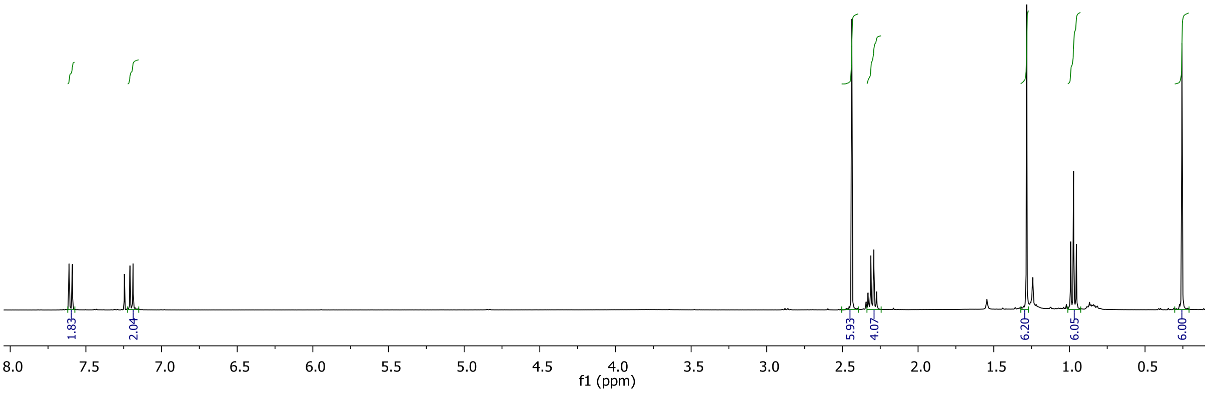

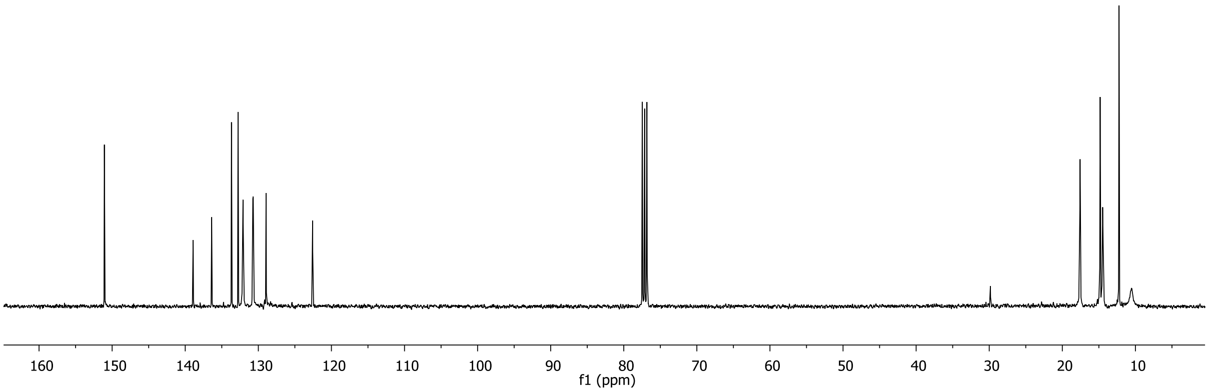

1.4 8-((4-diethylphosphonato)phenyl)-4,4-diphenyl-1,3,5,7-tetramethyl-2,6-diethyl-4-bora-3a,4a-diaza-*s*-indacene (**3a**)

2a (1.00 g, 1.74 mmol) was dissolved in anhydrous dimethyl sulfoxide (40 mL) and palladium acetate (0.117 g, 0.17 mmol), diethyl phosphite (0.25 mL, 1.91 mmol), *N*,*N*-diisopropylethylamine (0.91 mL, 5.21 mmol) and 1,4-bis(diphenylphosphino)butane (0.074 g, 0.17 mmol) were added. The mixture was degassed with nitrogen for 20 minutes before being heated at 90°C for three days. The reaction mixture was diluted with dichloromethane (100 mL) and washed with water (40 mL) and brine (40 mL), and dried over magnesium sulfate. Purification was performed by column chromatography on silica gel (ethyl acetate/petroleum ether 2:1, R_f = 0.5) to yield an orange solid (0.96 g, 87%). A sample suitable for X-ray crystallographic analysis was obtained from ethyl acetate/petroleum ether. **^{1}H NMR** (400 MHz, $CDCl_3$) δ 7.93 (dd, $^3J_{HH}$ = 7.8 Hz, $^3J_{HP}$ = 13.3 Hz, 2H), 7.49 (dd, $^3J_{HH}$ = 7.8 Hz, $^4J_{HP}$ = 4.1 Hz, 2H), 7.38-7.35 (m, 4H), 7.24-7.16 (m, 6H), 4.16 (m, 4H), 2.20 (q, $^3J_{HH}$ = 7.3 Hz, 4H), 2.01 (s, 6H), 1.75 (s, 6H), 1.35 (t, $^3J_{HH}$ = 6.8 Hz, 6H), 0.86 (t, $^3J_{HH}$ = 7.3 Hz, 6H) ppm; **^{13}C{^{1}H} NMR** (100 MHz, $CDCl_3$) δ 153.4, 150.2, 141.3, 138.9, 134.8, 133.8, 133.1, 132.0 (d, J_{CP} = 10.5 Hz), 130.2, 129.2 (d, J_{CP} = 15.3 Hz), 127.8, 127.1, 125.5, 62.2 (d, J_{CP} = 5.8 Hz), 17.7, 16.3 (d, J_{CP} = 5.8 Hz), 14.5, 14.1, 12.0 ppm; **^{31}P{^{1}H} NMR** (162 MHz, $CDCl_3$) δ 18.7 ppm; **^{11}B{^{1}H} NMR** (128 MHz, $CDCl_3$) δ -1.1 ppm; **IR** (neat): $\tilde{\nu}$ = 2960 (w), 1547 (s), 1474 (m), 1386 (s), 1303 (m), 1254 (w), 1168 (m), 1141 (m), 1032 (m), 970 (s), 773 (s) cm^{-1}; **MP**: 158-160°C; **HRMS** (AP$^+$) calc. $C_{39}H_{47}B_1N_2O_3P_1$ for $[M+H]^+$ 632.3448, found *m*/*z* 632.3462 (2.2 ppm).

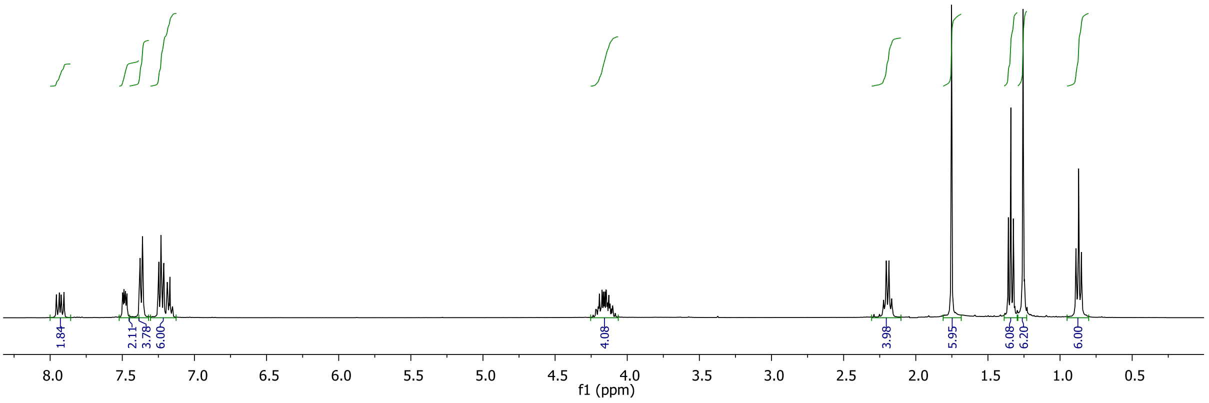

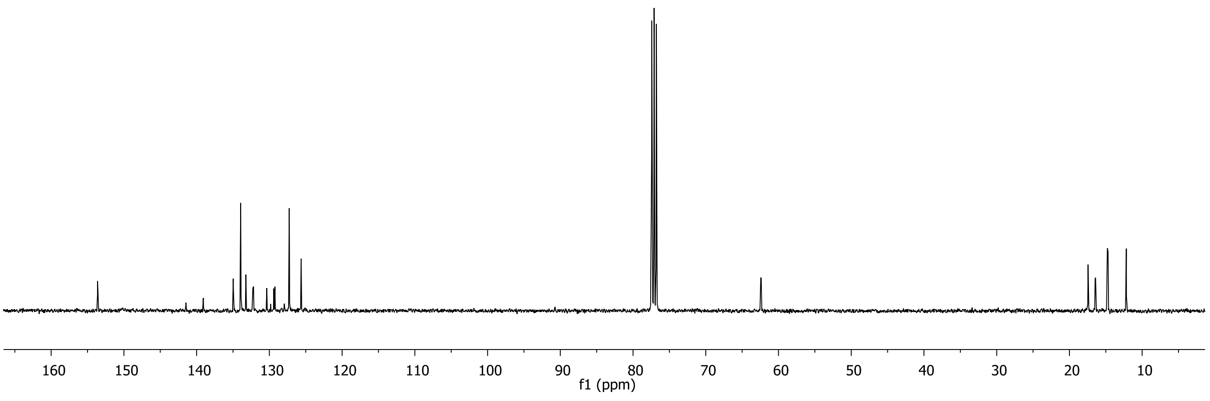

1.5 8-((4-diethylphosphonato)phenyl)-4,4-dimethyl-1,3,5,7-tetramethyl-2,6-diethyl-4-bora-3a,4a-diaza-*s*-indacene (**3b**)

2b (1.00 g, 2.22 mmol) was dissolved in anhydrous toluene (40 mL). Bis(dibenzylideneacetone)palladium (0.128 g, 0.22 mmol), diethyl phosphite (0.34 mL, 2.66 mmol), *N,N*-diisopropylethylamine (1.16 mL, 6.66 mmol) and 1,4-bis(diphenylphosphino)butane (0.095 g, 0.22 mmol) were added and the reaction mixture was degassed with nitrogen for 20 minutes then stirred at 90°C for 3 days. The reaction mixture was filtered then purified by column chromatography on silica gel (ethyl acetate/hexane 3:1, R_f = 0.4) to yield an orange solid (0.60 g, 53%). ^{1}**H NMR** (400 MHz, $CDCl_3$) δ 7.89 (dd, $^{3}J_{HH}$ = 7.8 Hz, $^{3}J_{HP}$ = 13.3 Hz, 2H), 7.42 (dd, $^{3}J_{HH}$ = 7.8 Hz, $^{4}J_{HP}$ = 4.1 Hz, 2H), 4.19-4.05 (m, 4H), 2.41 (s, 6H), 2.26 (q, $^{3}J_{HH}$ = 7.3 Hz, 4H), 1.30 (t, $^{3}J_{HH}$ = 6.9 Hz, 6H), 1.19 (s, 6H), 0.93 (t, $^{3}J_{HH}$ = 7.3 Hz, 6H), 0.24 (s, 6H) ppm; ^{13}**C{^{1}H} NMR** (100 MHz, $CDCl_3$) δ 150.8, 141.5 (d, J_{CP} = 2.9 Hz), 138.6, 133.2, 132.5, 131.9 (d, J_{CP} = 10.5 Hz), 129.3, 128.9 (d, J_{CP} = 15.3 Hz), 128.4, 62.0 (d, J_{CP} = 5.8 Hz), 17.2, 16.1 (d, J_{CP} = 5.8 Hz), 14.5, 14.1, 11.7, 10.2 ppm; ^{31}**P{^{1}H} NMR** (162 MHz, $CDCl_3$) δ 18.7 ppm; ^{11}**B{^{1}H} NMR** (128 MHz, $CDCl_3$) δ -1.7 ppm; **IR** (neat): $\tilde{\nu}$ = 2960 (w), 2931 (w), 1556 (s), 1453 (m), 1360 (m), 1322 (m), 1244 (m), 1172 (m), 1047 (m), 1014 (w), 945 (s) cm^{-1}; **MP**: 91-95°C; **HRMS** (ESI^{+}) calcd for $C_{29}H_{43}B_1N_2O_3P_1$ $[M+H]^+$ requires m/z 508.3135, found *m/z* 508.3133 (0.4 ppm).

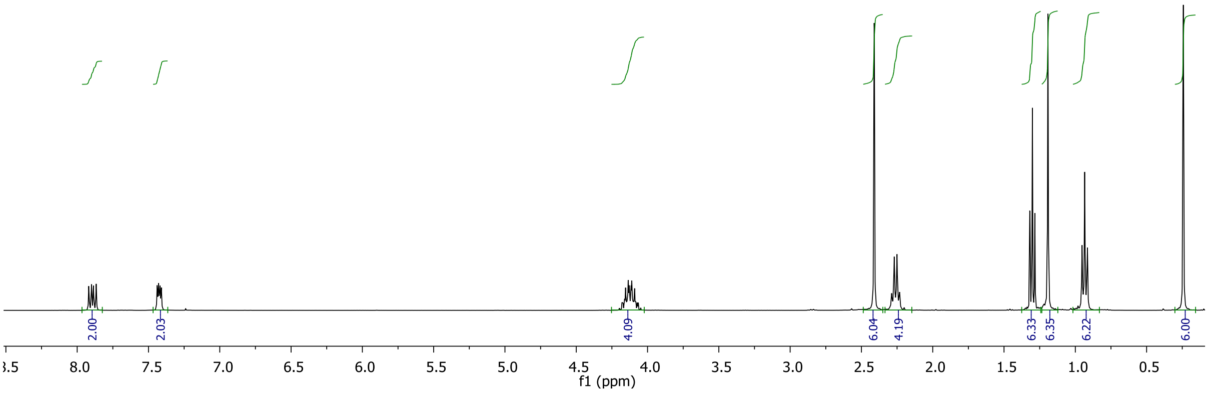

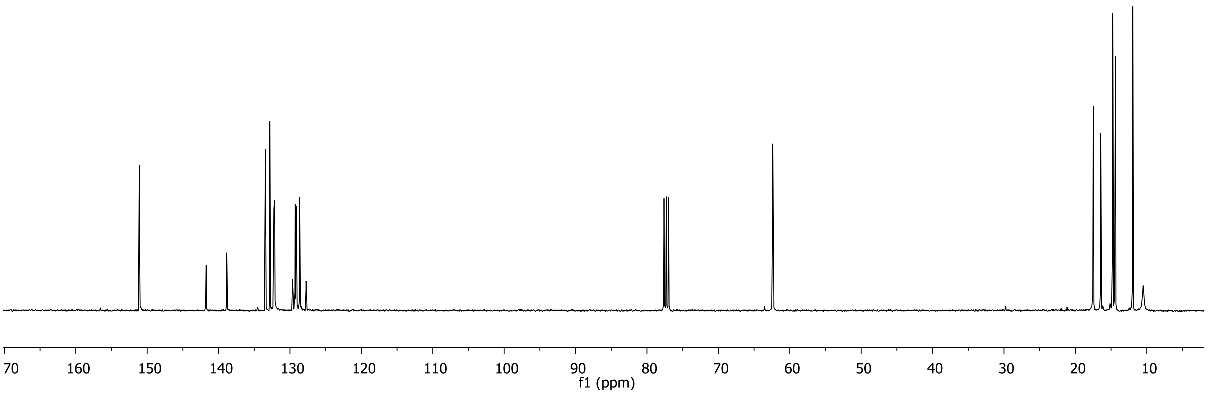

1.6 8-((4-phosphino)phenyl)-4,4-diphenyl-1,3,5,7-tetramethyl-2,6-diethyl-4-bora-3a,4a-diaza-*s*-indacene (**4a**)

Lithium aluminum hydride (3.16 mL, 3.16 mmol, 1.0M solution in tetrahydrofuran) was added to a Schlenk flask under nitrogen and cooled to -78°C in a dry ice/acetone bath; to the resultant white suspension chlorotrimethylsilane (0.40 mL, 3.16 mmol) was added. This reducing mixture was warmed to room temperature over ~30 min and the colorless solution was then cooled to -40°C in a dry ice/acetone bath. **3a** (1.00 g, 1.58 mmol) was dissolved in anhydrous tetrahydrofuran (100 mL), and this was added slowly to the reaction mixture. The cooling bath was removed and the reaction warmed to room temperature and stirred overnight to leave a red solution. The solution was concentrated *in vacuo* and degassed water (20 mL) was added dropwise to quench the reaction after first cooling it in an ice bath. The product was extracted with diethyl ether (3 x 20 mL) and dried over magnesium sulfate. Purification was performed by column chromatography on silica gel (dichloromethane/petroleum ether 1:2, R_f = 0.4) to yield an orange solid (0.77 g, 92%). **^{1}H NMR** (400 MHz, $CDCl_3$) δ 7.66-7.62 (m, 2H), 7.43-7.42 (m, 4H), 7.32-7.19 (m, 8H), 4.14 (d, $^1J_{HP}$ = 202.5 Hz, 2H), 2.25 (q, $^3J_{HH}$ = 7.8 Hz, 4H), 1.80 (s, 6H), 1.36 (s, 6H), 0.93 (t, $^3J_{HH}$ = 7.8 Hz, 6H) ppm; **^{13}C{^{1}H} NMR** (100 MHz, $CDCl_3$) δ 153.0, 150.3, 139.9, 136.8, 135.1, 134.9 (d, J_{CP} = 15.3 Hz), 133.8, 132.8, 130.6, 129.0, 128.8 (d, J_{CP} = 5.6 Hz), 127.1, 125.4, 17.3, 14.7, 14.5, 12.1 ppm; **^{31}P-^{1}H NMR** (162 MHz, $CDCl_3$) δ -121.5 (tt, $^1J_{PH}$ = 202.5 Hz, $^3J_{PH}$ = 7.6 Hz) ppm; **^{11}B{^{1}H} NMR** (128 MHz, $CDCl_3$) δ -1.0 ppm; **IR** (neat): $\tilde{\nu}$ = 2963 (w), 2928 (w), 2869 (w), 2285 (w) (PH), 1545 (s), 1469 (m), 1393 (m), 1303 (s), 1169 (s), 1142 (m), 962 (s), 774 (s) cm^{-1}; **MP**: 207-210°C; **HRMS** (ESI^+) calc. for $C_{35}H_{39}B_1N_2P_1$ $[M+H]^+$ requires m/z 528.2975, found *m/z* 528.2972 (0.5 ppm).

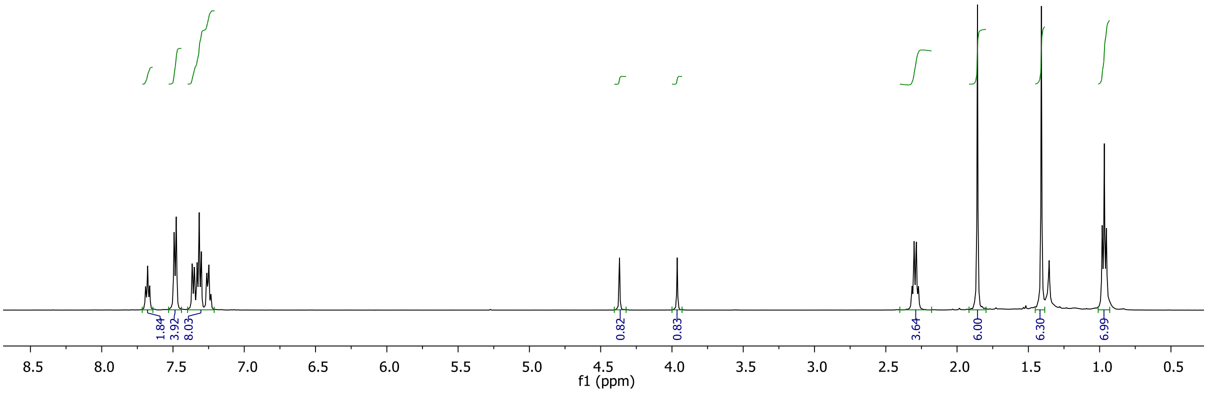

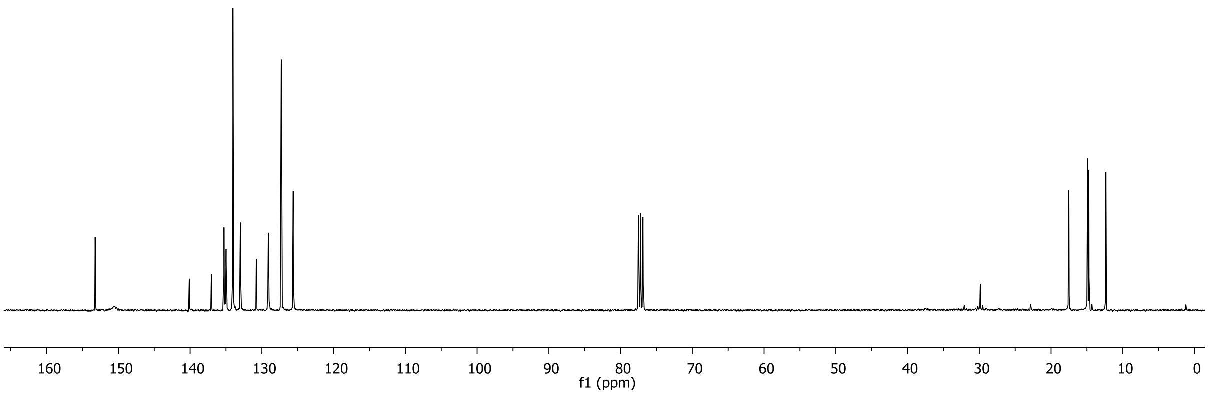

1.7 8-((4-phosphino)phenyl)-4,4-dimethyl-1,3,5,7-tetramethyl-2,6-diethyl-4-bora-3a,4a-diaza-*s*-indacene (**4b**)

Prepared in the same manner as **4a**, using 3.93 mL (3.93 mmol) of lithium aluminum hydride (1.0M solution in tetrahydrofuran), 0.50 mL (3.93 mmol) of chlorotrimethylsilane and 1.00 g (1.97 mmol) of **3b**. Purification was performed by column chromatography on silica gel (chloroform/hexane 1:4, R_f = 0.4) to yield an orange solid (0.74 g, 93%). A sample suitable for X-ray crystallographic analysis was obtained from chloroform. **^{1}H NMR** (500 MHz, $CDCl_3$) δ 7.60 (m, 2H), 7.26 (m, 2H), 4.11 (d, $^1J_{HP}$ = 202.5 Hz, 2H), 2.47 (s, 6H), 2.33 (q, $^3J_{HH}$ = 7.8 Hz, 4H), 1.29 (s, 6H), 1.00 (t, $^3J_{HH}$ = 7.8 Hz, 6H), 0.30 (s, 6H) ppm; **^{13}C{^{1}H} NMR** (100 MHz, $CDCl_3$) δ 150.7, 139.7, 137.2 134.9 (d, J_{CP} = 15.3 Hz), 133.7, 132.4, 128.9 (d, J_{CP} = 6.7 Hz), 128.8, 128.7, 17.4, 14.7, 14.3, 11.9, 10.4 ppm; **^{31}P-^{1}H NMR** (202 MHz, $CDCl_3$) δ -121.7 (tt, $^1J_{PH}$ = 202.5 Hz, $^3J_{PH}$ = 7.44 Hz) ppm; **^{11}B{^{1}H} NMR** (128 MHz, $CDCl_3$) δ -2.1 ppm; **IR** (neat): $\tilde{\nu}$ = 2958 (w), 2925 (w), 2361 (w) (PH), 2341 (w) (PH), 1551 (s), 1470 (s), 1531 (m), 1167 (s), 1143 (m), 1110 (m), 1060 (m), 942 (s) cm^{-1}; **MP**; 196-198°C; **HRMS** (ESI^+) calcd for $C_{25}H_{35}B_1N_2P_1$ $[M+H]^+$ requires m/z 404.2662, found *m/z* 404.2669 (1.8 ppm).

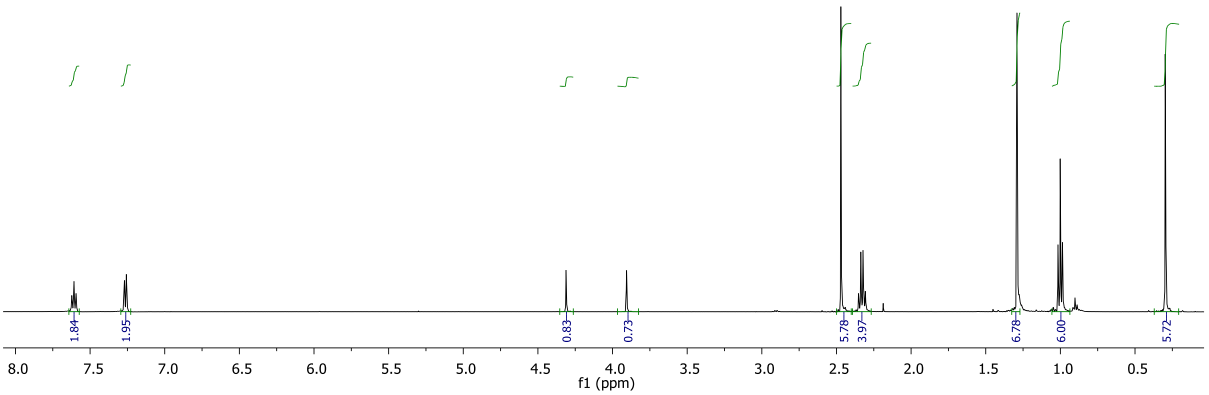

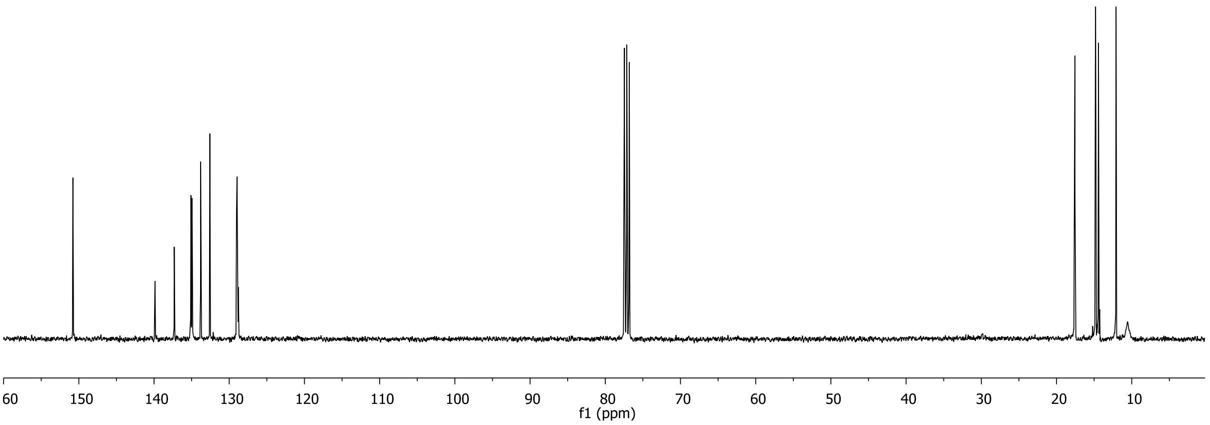

1.8 8-(4-(bis-2-ethyldiphenylphosphino)-phenylphosphane)-4,4-dimethyl-1,3,5,7-tetramethyl-2,6-diethyl-4-bora-3a,4a-diaza-*s*-indacene (**5b**)

4b (0.150 g, 0.37 mmol) was dissolved in anhydrous toluene (10 mL), to this [Pt(norbornene)$_3$] (0.018 g, 0.037 mmol) and vinyldiphenylphosphane (0.16 mL, 0.78 mmol) were added. The reaction was stirred at reflux for five days under nitrogen. On completion of the reaction, the volatiles were removed *in vacuo* to leave a deep red solid. Purification was performed by column chromatography on silica gel (hexane/dichloromethane 1:1, R_f = 0.4) to yield an orange solid (0.21 g, 70%). **^{1}H NMR** (500 MHz, $CDCl_3$) δ 7.46 (m, 2H), 7.39-7.31 (m, 22H), 2.49 (s, 6H), 2.31 (q, $^3J_{HH}$ = 7.3 Hz, 4H), 2.05-1.80 (m, 8H), 1.21 (s, 6H), 1.00 (t, $^3J_{HH}$ = 7.3 Hz, 6H), 0.34 (s, 6H) ppm; **^{13}C{^{1}H} NMR** (126 MHz, $CDCl_3$) δ 150.9, 139.9, 138.4-138.1 (m), 137.4 (m), 133.7, 133.1-132.7 (m), 130.9 (m), 129.2-128.4 (m), 23.8 (m, CH_2-P), 23.4 (m, CH_2-P), 17.6, 14.8, 14.5, 12.0, 10.6 ppm; **^{31}P{^{1}H} NMR** (202 MHz, $CDCl_3$) δ -12.2 (d, $^3J_{PP}$ = 27.3 Hz, 2P), -16.4 (t, $^3J_{PP}$ = 27.3 Hz, 1P) ppm; **^{11}B{^{1}H} NMR** (160 MHz, $CDCl_3$) δ -1.8 ppm; **IR** (neat): $\tilde{\nu}$ = 2961 (w), 2929 (w), 1557 (s), 1433 (m), 1386 (m), 1323 (s), 1174 (s), 1019, 946 (s) cm^{-1}; **MP**: 178-181°C; **HRMS** (AP$^+$) calcd for $C_{53}H_{61}B_1N_2P_3$ [M+H]$^+$ requires m/z 828.4171, found *m/z* 828.4166 (0.7 ppm).

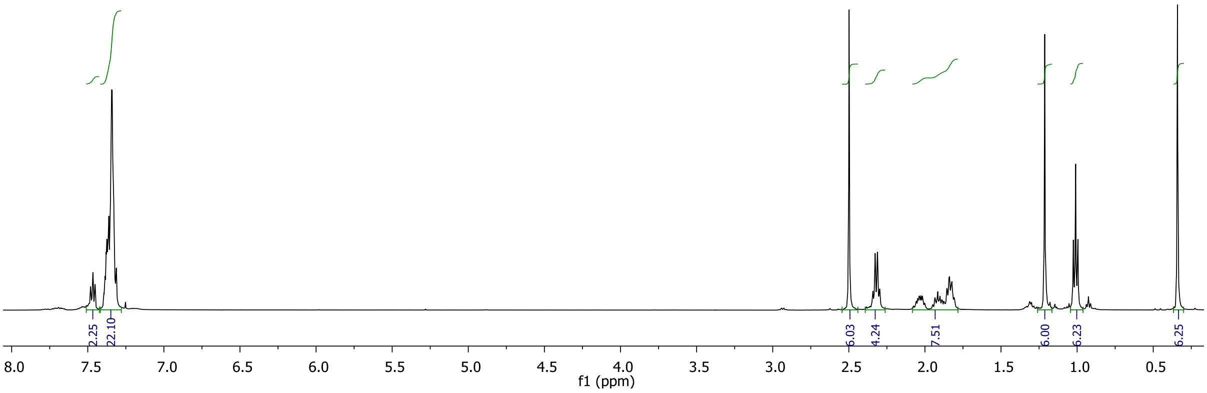

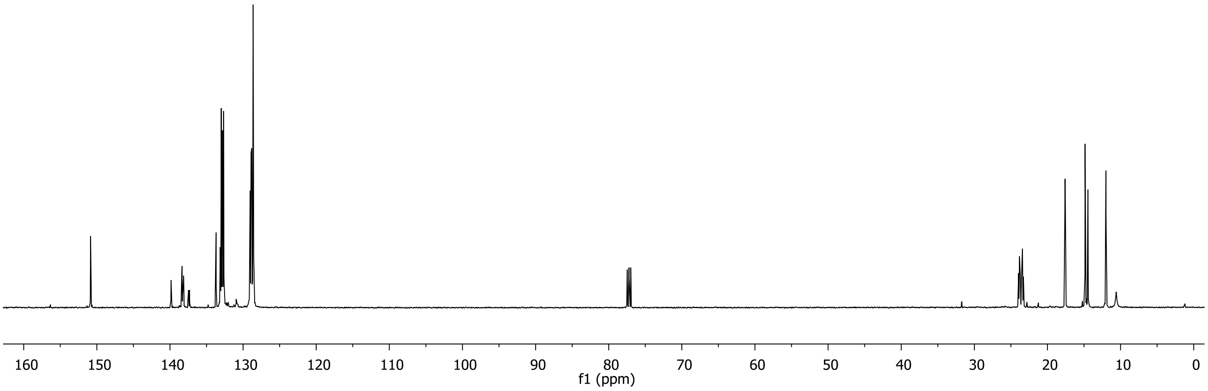

1.9 *cis,mer*-[ReCl(CO)$_2$(**5b**)] (**6b**)

5b (0.050 g, 0.060 mmol) and [ReCl(CO)$_3$(PPh$_3$)$_2$] (0.050 g, 0.060 mmol) were dissolved in anhydrous mesitylene (2 mL) and stirred at reflux for 4 hours. After passing the reaction mixture through a pad of silica, eluting first with hexane, followed by dichloromethane, an orange solid was obtained (0.051 g, 82%). A sample suitable for X-ray crystallographic analysis was obtained from tetrahydrofuran/pentane. **^{1}H NMR** (500 MHz, CDCl$_3$) δ 7.78-7.75 (m, 4H), 7.53-7.45 (m, 6H), 7.40-7.24 (m, 14H), 3.52-3.40 (m, 2H), 2.88-2.84 (m, 2H), 2.58-2.47 (m, 4H), 2.45 (s, 6H), 2.30 (q, $^3J_{HH}$ = 7.3 Hz, 4H), 1.14 (s, 6H), 0.99 (t, $^3J_{HH}$ = 7.3 Hz, 6H), 0.26 (s, 6H) ppm; **^{13}C{^{1}H} NMR** (126 MHz, CDCl$_3$) δ 201.8 (dt, $^2J_{CP}$ = 59.6 Hz, $^2J_{CP}$ = 6.7 Hz, CO), 196.2 (m, CO), 151.1, 140.0, 138.7, 137.8 (m), 134.4 (m), 133.3-131.7 (m), 129.8-128.2 (m), 33.1 (m, CH$_2$-P), 25.5 (dt, $^1J_{CP}$ = 27.8 Hz, $^2J_{CP}$ = 6.7 Hz, CH$_2$-P), 17.6, 14.8, 14.4, 11.8, 10.5 ppm; **^{31}P{^{1}H} NMR** (202 MHz, CDCl$_3$) δ 79.8 (1P), 37.7 (2P) ppm; **^{11}B{^{1}H} NMR** (160 MHz, CDCl$_3$) δ -2.0 ppm; **IR** (neat): $\tilde{\nu}$ = 1933 (s), 1852 (s) (CO) cm^{-1}; **HRMS** (ESI$^+$) calc. for C$_{55}$H$_{61}$B$_1$Cl$_1$N$_2$O$_2$P$_3$Re$_1$ [M+H]$^+$ requires m/z 1104.3288, found *m/z* 1104.3284 (0.4 ppm).

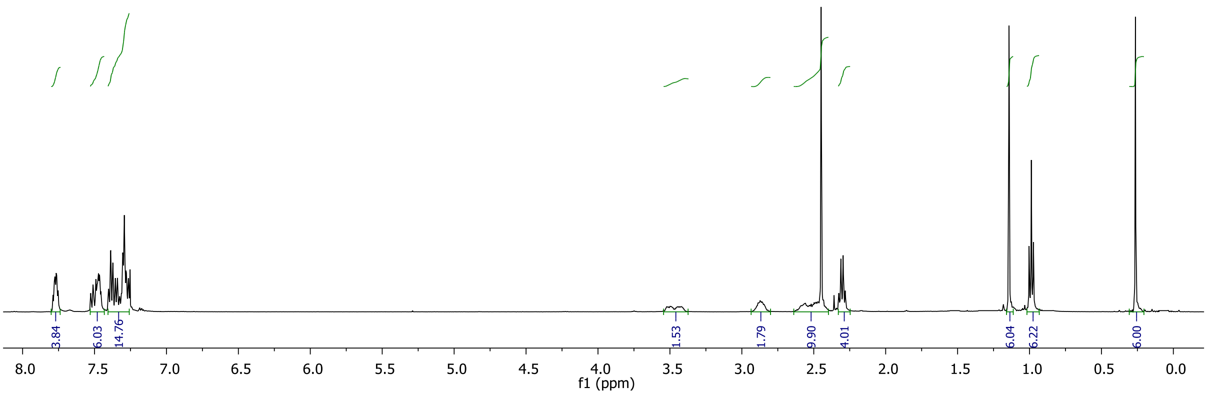

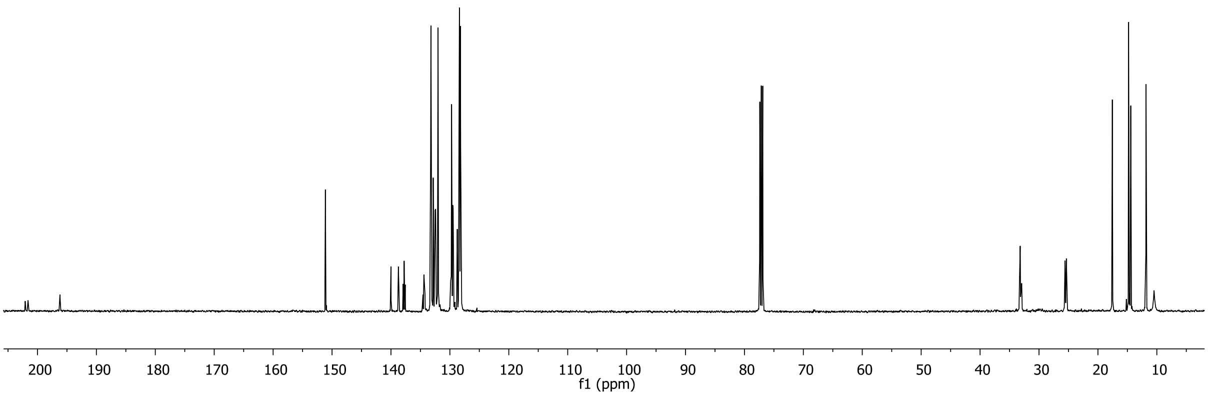

2 Primary Phosphane Air Stability Experiments

2.1 Solid-state experiments

These were carried out on a scale of 20 mg, by placing the solid in a glass vial open to air in the dark. The vial was isolated from any combustible materials as a precautionary measure, and after analysis, the samples were treated with commercial bleach prior to disposal. The ratios of phosphane oxide, H-phosphinic acid and phosphonic acid to phosphane in the oxidation studies were obtained from integration of the ^{31}P-^{1}H coupled spectra recorded in dry, degassed *d*-chloroform, with a relaxation time of 3 seconds and with NOE effects removed.

2.2 Solution-state experiments

These were carried out on a scale of 20 mg, by dissolving the solid in bench *d*-chloroform in a glass vial open to air in the dark. An NMR analysis was performed immediately to record any initial oxidation, and then every 24h each sample was analysed again over 7 days (using the same integrating technique as for the solid-state experiments). As above, the sample vial was isolated from any combustible materials as a precautionary measure, and after analysis, the samples were treated with commercial bleach prior to disposal.

3 X-ray Crystallography

3.1 General information

Crystal data and summaries of experimental and computing procedures are given in the following tables, together with full results of atomic coordinates, bond lengths and angles, torsion angles for the simpler structures not affected by disorder, and displacement parameters.

Data were collected on an Oxford Diffraction (now Agilent Technologies) Gemini A Ultra diffractometer for **2a**, **4b** and **6b** (CrysAlisPro software, Oxford Diffraction, 2008), and on beamline I19 of Diamond Light Source with synchrotron radiation for **3a** and **2b** (CrystalClear data collection software, Rigaku Corporation, 2010; APEX2 software for integration and data processing, Bruker AXS, 2010; image conversion software provided by Professor Simon Parsons, Edinburgh University, UK). Corrections were made for absorption, along with application of frame scaling, using repeated and symmetry-equivalent reflections (SADABS and TWINABS, G. M. Sheldrick, University of Göttingen, Germany, 2008–2011).

In all cases, structures were solved by direct methods and refined by full-matrix least-squares techniques using programs of the SHELX family (G. M. Sheldrick, *Acta Crystallogr. Sect. A*, **2008**, *64*, 112–122). Minor disorder problems were resolved using restraints on geometry and displacement parameters. H atoms were treated as riding in calculated idealized positions.

4b is a non-merohedral twin, for which both components were identified and treated in the data collection and processing, and the refinement indicated a 17.38(9)% contribution for the minor component. **2b** is a pseudo-merohedral twin with the two components overlapping in the diffraction pattern; the contribution of the minor component was refined to 25.61(12)%.

The crystal structure of **6b** is non-centrosymmetric, and the correct hand was confirmed by successful refinement of the 'absolute structure' parameter to a value of essentially zero (H. D. Flack, *Acta Crystallogr. Sect. A*, **1983**, *39*, 876–881).

The crystal structures of **3a** and **6b** contain highly disordered solvent, which could not be modeled as discrete atoms and was therefore treated by the SQUEEZE procedure of PLATON (A. L. Spek, *J. Appl. Crystallogr.* **2003**, *36*, 7–13).

3.2 Crystallographic data for **2a**

Table 1. Crystal data and structure refinement for **2a**.

Identification code	2a	
Chemical formula (moiety)	$C_{35}H_{36}BBrN_2$	
Chemical formula (total)	$C_{35}H_{36}BBrN_2$	
Formula weight	575.38	
Temperature	150(2) K	
Radiation, wavelength	MoKα, 0.71073 Å	
Crystal system, space group	monoclinic, $P2_1/n$	
Unit cell parameters	a = 9.2070(2) Å	α = 90°
	b = 31.4746(7) Å	β = 90.324(3)°
	c = 19.9059(6) Å	γ = 90°
Cell volume	5768.4(2) $Å^3$	
Z	8	
Calculated density	1.325 g/cm^3	
Absorption coefficient μ	1.452 mm^{-1}	
F(000)	2400	
Crystal colour and size	red, 0.42 × 0.34 × 0.20 mm^3	
Reflections for cell refinement	8969 (θ range 2.8 to 29.5°)	
Data collection method	Oxford Diffraction Gemini A Ultra diffractometer	
	ω scans	
θ range for data collection	2.8 to 29.6°	
Index ranges	h –12 to 12, k –43 to 39, l –19 to 25	
Completeness to θ = 26.0°	99.8 %	
Reflections collected	49783	
Independent reflections	14198 (R_{int} = 0.0841)	
Reflections with $F^2>2\sigma$	5487	
Absorption correction	semi-empirical from equivalents	
Min. and max. transmission	0.581 and 0.760	
Structure solution	direct methods	
Refinement method	Full-matrix least-squares on F^2	
Weighting parameters a, b	0.0164, 0.0000	
Data / restraints / parameters	14198 / 0 / 716	
Final R indices [$F^2>2\sigma$]	R1 = 0.0362, wR2 = 0.0488	
R indices (all data)	R1 = 0.1393, wR2 = 0.0564	
Goodness-of-fit on F^2	0.655	
Extinction coefficient	0.00018(4)	
Largest and mean shift/su	0.003 and 0.000	
Largest diff. peak and hole	0.28 and –0.45 e $Å^{-3}$	

Table 2. Atomic coordinates and equivalent isotropic displacement parameters (Å^2) for **2a**. U_{eq} is defined as one third of the trace of the orthogonalized U^{ij} tensor.

	x	y	z	U_{eq}
Br(1)	0.82930(3)	0.200002(7)	0.641215(15)	0.04673(9)
N(1)	0.75743(17)	–0.00867(5)	0.39058(9)	0.0188(5)
N(2)	0.87768(17)	0.04012(5)	0.30839(9)	0.0173(4)
B(1)	0.7773(3)	–0.00086(7)	0.31220(14)	0.0210(7)
C(1)	0.7074(2)	–0.04336(6)	0.42349(13)	0.0216(6)
C(2)	0.6769(2)	–0.03304(6)	0.49091(12)	0.0208(6)
C(3)	0.7090(2)	0.00927(6)	0.50078(12)	0.0189(5)
C(4)	0.7621(2)	0.02448(6)	0.43789(12)	0.0183(5)
C(5)	0.8193(2)	0.06399(6)	0.42014(12)	0.0188(6)
C(6)	0.8777(2)	0.07194(6)	0.35717(12)	0.0183(6)
C(7)	0.9491(2)	0.10811(6)	0.32988(13)	0.0216(6)
C(8)	0.9928(2)	0.09750(6)	0.26637(12)	0.0199(6)
C(9)	0.9450(2)	0.05530(6)	0.25331(12)	0.0183(5)
C(10)	0.6834(2)	–0.08612(6)	0.39349(12)	0.0286(6)
C(11)	0.7328(3)	–0.07921(8)	0.59214(14)	0.0537(9)
C(12)	0.6192(2)	–0.06322(6)	0.54306(12)	0.0293(6)
C(13)	0.6866(2)	0.03237(6)	0.56511(12)	0.0278(6)
C(14)	0.9753(2)	0.15068(6)	0.36286(12)	0.0299(6)
C(15)	0.9838(2)	0.15631(6)	0.17875(13)	0.0379(7)
C(16)	1.0766(2)	0.12491(6)	0.21842(12)	0.0259(6)
C(17)	0.9553(2)	0.03194(6)	0.18842(11)	0.0243(6)
C(18)	0.8242(2)	0.09755(6)	0.47256(12)	0.0198(6)
C(19)	0.9351(2)	0.09874(6)	0.51938(12)	0.0229(6)
C(20)	0.9370(2)	0.12925(6)	0.56949(12)	0.0268(6)
C(21)	0.8252(3)	0.15850(6)	0.57219(13)	0.0278(6)
C(22)	0.7138(2)	0.15802(6)	0.52636(13)	0.0285(6)
C(23)	0.7147(2)	0.12782(6)	0.47582(12)	0.0266(6)
C(24)	0.8621(2)	–0.04074(6)	0.27923(12)	0.0185(5)
C(25)	0.8085(2)	–0.06585(6)	0.22697(12)	0.0263(6)
C(26)	0.8847(3)	–0.10075(6)	0.20247(13)	0.0323(7)
C(27)	1.0181(3)	–0.11104(7)	0.23012(14)	0.0368(7)
C(28)	1.0758(2)	–0.08612(7)	0.27967(14)	0.0313(7)
C(29)	0.9980(2)	–0.05161(6)	0.30427(12)	0.0264(6)
C(30)	0.6232(2)	0.01153(6)	0.27854(11)	0.0183(5)
C(31)	0.5928(2)	0.05202(6)	0.25317(12)	0.0245(6)
C(32)	0.4615(2)	0.06266(7)	0.22377(13)	0.0317(7)
C(33)	0.3535(2)	0.03289(7)	0.21821(14)	0.0375(7)
C(34)	0.3770(2)	–0.00724(7)	0.24342(13)	0.0338(7)
C(35)	0.5087(2)	–0.01735(7)	0.27316(12)	0.0261(6)
Br(2)	0.12922(3)	0.518817(7)	0.570969(16)	0.04659(9)
N(3)	0.31859(17)	0.29814(5)	0.36694(9)	0.0193(4)
N(4)	0.55854(18)	0.30444(5)	0.42538(9)	0.0204(5)
B(2)	0.4415(3)	0.27075(7)	0.40278(15)	0.0209(7)
C(36)	0.2110(2)	0.28577(6)	0.32514(12)	0.0227(6)
C(37)	0.0999(2)	0.31673(6)	0.32441(12)	0.0248(6)
C(38)	0.1421(2)	0.34900(6)	0.36729(12)	0.0230(6)
C(39)	0.2783(2)	0.33753(6)	0.39478(12)	0.0191(6)
C(40)	0.3754(2)	0.35847(5)	0.43750(12)	0.0184(5)
C(41)	0.5130(2)	0.34367(6)	0.45140(12)	0.0197(6)
C(42)	0.6299(2)	0.36178(6)	0.48834(12)	0.0234(6)
C(43)	0.7451(2)	0.33369(6)	0.48355(12)	0.0227(6)
C(44)	0.6971(2)	0.29862(6)	0.44578(12)	0.0239(6)
C(45)	0.2084(2)	0.24495(6)	0.28666(12)	0.0297(6)
C(46)	–0.0345(3)	0.34424(7)	0.22324(14)	0.0488(8)

C(47)	−0.0388(2)	0.31534(6)	0.28363(13)	0.0338(7)
C(48)	0.0558(2)	0.38930(6)	0.37747(13)	0.0323(7)
C(49)	0.6342(2)	0.40351(6)	0.52524(13)	0.0355(7)
C(50)	0.9008(3)	0.32123(8)	0.58690(15)	0.0558(8)
C(51)	0.8922(2)	0.33811(7)	0.51546(13)	0.0353(7)
C(52)	0.7842(2)	0.25948(6)	0.43243(13)	0.0327(7)
C(53)	0.3230(2)	0.39844(6)	0.46985(12)	0.0212(6)
C(54)	0.3425(2)	0.43797(6)	0.44063(13)	0.0316(7)
C(55)	0.2862(2)	0.47394(6)	0.47125(13)	0.0340(7)
C(56)	0.2115(2)	0.47007(6)	0.53047(13)	0.0307(6)
C(57)	0.1909(2)	0.43123(7)	0.56050(13)	0.0295(6)
C(58)	0.2476(2)	0.39549(6)	0.52980(12)	0.0248(6)
C(59)	0.3720(2)	0.25078(6)	0.47013(12)	0.0177(6)
C(60)	0.2317(2)	0.23350(6)	0.46979(13)	0.0327(7)
C(61)	0.1694(3)	0.21607(7)	0.52658(15)	0.0382(7)
C(62)	0.2442(3)	0.21532(6)	0.58652(14)	0.0334(7)
C(63)	0.3811(3)	0.23265(6)	0.58868(13)	0.0348(7)
C(64)	0.4416(2)	0.25011(6)	0.53160(13)	0.0269(6)
C(65)	0.5076(2)	0.23686(6)	0.35038(12)	0.0188(6)
C(66)	0.5755(2)	0.24993(6)	0.29154(13)	0.0289(6)
C(67)	0.6297(2)	0.22141(7)	0.24488(13)	0.0350(7)
C(68)	0.6157(2)	0.17825(7)	0.25678(13)	0.0317(6)
C(69)	0.5508(2)	0.16425(6)	0.31422(13)	0.0310(7)
C(70)	0.4978(2)	0.19325(6)	0.36043(12)	0.0249(6)

Table 3. Bond lengths [Å] and angles [°] for **2a**.

Br(1)–C(21)	1.896(2)	N(1)–B(1)	1.591(3)
N(1)–C(1)	1.355(2)	N(1)–C(4)	1.406(2)
N(2)–B(1)	1.589(3)	N(2)–C(6)	1.395(2)
N(2)–C(9)	1.350(3)	B(1)–C(24)	1.619(3)
B(1)–C(30)	1.613(3)	C(1)–C(2)	1.410(3)
C(1)–C(10)	1.489(3)	C(2)–C(3)	1.378(3)
C(2)–C(12)	1.506(3)	C(3)–C(4)	1.429(3)
C(3)–C(13)	1.488(3)	C(4)–C(5)	1.397(3)
C(5)–C(6)	1.389(3)	C(5)–C(18)	1.485(3)
C(6)–C(7)	1.424(3)	C(7)–C(8)	1.370(3)
C(7)–C(14)	1.511(3)	C(8)–C(9)	1.423(3)
C(8)–C(16)	1.503(3)	C(9)–C(17)	1.490(3)
C(10)–H(10A)	0.980	C(10)–H(10B)	0.980
C(10)–H(10C)	0.980	C(11)–H(11A)	0.980
C(11)–H(11B)	0.980	C(11)–H(11C)	0.980
C(11)–C(12)	1.514(3)	C(12)–H(12A)	0.990
C(12)–H(12B)	0.990	C(13)–H(13A)	0.980
C(13)–H(13B)	0.980	C(13)–H(13C)	0.980
C(14)–H(14A)	0.980	C(14)–H(14B)	0.980
C(14)–H(14C)	0.980	C(15)–H(15A)	0.980
C(15)–H(15B)	0.980	C(15)–H(15C)	0.980
C(15)–C(16)	1.524(3)	C(16)–H(16A)	0.990
C(16)–H(16B)	0.990	C(17)–H(17A)	0.980
C(17)–H(17B)	0.980	C(17)–H(17C)	0.980
C(18)–C(19)	1.380(3)	C(18)–C(23)	1.388(3)
C(19)–H(19)	0.950	C(19)–C(20)	1.385(3)
C(20)–H(20)	0.950	C(20)–C(21)	1.382(3)
C(21)–C(22)	1.369(3)	C(22)–H(22)	0.950
C(22)–C(23)	1.384(3)	C(23)–H(23)	0.950
C(24)–C(25)	1.395(3)	C(24)–C(29)	1.387(3)
C(25)–H(25)	0.950	C(25)–C(26)	1.393(3)
C(26)–H(26)	0.950	C(26)–C(27)	1.382(3)
C(27)–H(27)	0.950	C(27)–C(28)	1.365(3)
C(28)–H(28)	0.950	C(28)–C(29)	1.392(3)
C(29)–H(29)	0.950	C(30)–C(31)	1.398(3)
C(30)–C(35)	1.395(3)	C(31)–H(31)	0.950
C(31)–C(32)	1.382(3)	C(32)–H(32)	0.950
C(32)–C(33)	1.370(3)	C(33)–H(33)	0.950
C(33)–C(34)	1.376(3)	C(34)–H(34)	0.950
C(34)–C(35)	1.383(3)	C(35)–H(35)	0.950
Br(2)–C(56)	1.893(2)	N(3)–B(2)	1.589(3)
N(3)–C(36)	1.348(3)	N(3)–C(39)	1.408(2)
N(4)–B(2)	1.575(3)	N(4)–C(41)	1.404(2)
N(4)–C(44)	1.349(2)	B(2)–C(59)	1.616(3)
B(2)–C(65)	1.614(3)	C(36)–C(37)	1.413(3)
C(36)–C(45)	1.496(3)	C(37)–C(38)	1.381(3)
C(37)–C(47)	1.509(3)	C(38)–C(39)	1.413(3)
C(38)–C(48)	1.511(3)	C(39)–C(40)	1.395(3)
C(40)–C(41)	1.377(3)	C(40)–C(53)	1.494(3)
C(41)–C(42)	1.419(3)	C(42)–C(43)	1.384(3)
C(42)–C(49)	1.505(3)	C(43)–C(44)	1.406(3)
C(43)–C(51)	1.499(3)	C(44)–C(52)	1.495(3)
C(45)–H(45A)	0.980	C(45)–H(45B)	0.980
C(45)–H(45C)	0.980	C(46)–H(46A)	0.980
C(46)–H(46B)	0.980	C(46)–H(46C)	0.980
C(46)–C(47)	1.508(3)	C(47)–H(47A)	0.990
C(47)–H(47B)	0.990	C(48)–H(48A)	0.980
C(48)–H(48B)	0.980	C(48)–H(48C)	0.980

C(49)–H(49A)	0.980	C(49)–H(49B)	0.980
C(49)–H(49C)	0.980	C(50)–H(50A)	0.980
C(50)–H(50B)	0.980	C(50)–H(50C)	0.980
C(50)–C(51)	1.520(3)	C(51)–H(51A)	0.990
C(51)–H(51B)	0.990	C(52)–H(52A)	0.980
C(52)–H(52B)	0.980	C(52)–H(52C)	0.980
C(53)–C(54)	1.386(3)	C(53)–C(58)	1.387(3)
C(54)–H(54)	0.950	C(54)–C(55)	1.387(3)
C(55)–H(55)	0.950	C(55)–C(56)	1.374(3)
C(56)–C(57)	1.374(3)	C(57)–H(57)	0.950
C(57)–C(58)	1.384(3)	C(58)–H(58)	0.950
C(59)–C(60)	1.401(3)	C(59)–C(64)	1.378(3)
C(60)–H(60)	0.950	C(60)–C(61)	1.384(3)
C(61)–H(61)	0.950	C(61)–C(62)	1.374(3)
C(62)–H(62)	0.950	C(62)–C(63)	1.374(3)
C(63)–H(63)	0.950	C(63)–C(64)	1.382(3)
C(64)–H(64)	0.950	C(65)–C(66)	1.393(3)
C(65)–C(70)	1.390(2)	C(66)–H(66)	0.950
C(66)–C(67)	1.387(3)	C(67)–H(67)	0.950
C(67)–C(68)	1.385(3)	C(68)–H(68)	0.950
C(68)–C(69)	1.366(3)	C(69)–H(69)	0.950
C(69)–C(70)	1.387(3)	C(70)–H(70)	0.950

B(1)–N(1)–C(1)	129.78(18)	B(1)–N(1)–C(4)	122.60(16)
C(1)–N(1)–C(4)	106.45(18)	B(1)–N(2)–C(6)	123.18(18)
B(1)–N(2)–C(9)	126.58(19)	C(6)–N(2)–C(9)	108.25(16)
N(1)–B(1)–N(2)	104.03(18)	N(1)–B(1)–C(24)	109.62(17)
N(1)–B(1)–C(30)	109.82(18)	N(2)–B(1)–C(24)	109.12(17)
N(2)–B(1)–C(30)	107.13(16)	C(24)–B(1)–C(30)	116.4(2)
N(1)–C(1)–C(2)	110.16(18)	N(1)–C(1)–C(10)	125.8(2)
C(2)–C(1)–C(10)	124.06(19)	C(1)–C(2)–C(3)	108.33(19)
C(1)–C(2)–C(12)	125.70(19)	C(3)–C(2)–C(12)	126.0(2)
C(2)–C(3)–C(4)	105.8(2)	C(2)–C(3)–C(13)	124.3(2)
C(4)–C(3)–C(13)	129.80(18)	N(1)–C(4)–C(3)	109.20(17)
N(1)–C(4)–C(5)	120.1(2)	C(3)–C(4)–C(5)	130.6(2)
C(4)–C(5)–C(6)	122.5(2)	C(4)–C(5)–C(18)	117.7(2)
C(6)–C(5)–C(18)	119.72(18)	N(2)–C(6)–C(5)	120.03(18)
N(2)–C(6)–C(7)	107.86(19)	C(5)–C(6)–C(7)	132.1(2)
C(6)–C(7)–C(8)	107.21(18)	C(6)–C(7)–C(14)	128.1(2)
C(8)–C(7)–C(14)	124.73(19)	C(7)–C(8)–C(9)	107.68(18)
C(7)–C(8)–C(16)	126.91(19)	C(9)–C(8)–C(16)	125.4(2)
N(2)–C(9)–C(8)	108.96(19)	N(2)–C(9)–C(17)	124.16(17)
C(8)–C(9)–C(17)	126.7(2)	C(1)–C(10)–H(10A)	109.5
C(1)–C(10)–H(10B)	109.5	C(1)–C(10)–H(10C)	109.5
H(10A)–C(10)–H(10B)	109.5	H(10A)–C(10)–H(10C)	109.5
H(10B)–C(10)–H(10C)	109.5	H(11A)–C(11)–H(11B)	109.5
H(11A)–C(11)–H(11C)	109.5	H(11A)–C(11)–C(12)	109.5
H(11B)–C(11)–H(11C)	109.5	H(11B)–C(11)–C(12)	109.5
H(11C)–C(11)–C(12)	109.5	C(2)–C(12)–C(11)	114.18(18)
C(2)–C(12)–H(12A)	108.7	C(2)–C(12)–H(12B)	108.7
C(11)–C(12)–H(12A)	108.7	C(11)–C(12)–H(12B)	108.7
H(12A)–C(12)–H(12B)	107.6	C(3)–C(13)–H(13A)	109.5
C(3)–C(13)–H(13B)	109.5	C(3)–C(13)–H(13C)	109.5
H(13A)–C(13)–H(13B)	109.5	H(13A)–C(13)–H(13C)	109.5
H(13B)–C(13)–H(13C)	109.5	C(7)–C(14)–H(14A)	109.5
C(7)–C(14)–H(14B)	109.5	C(7)–C(14)–H(14C)	109.5
H(14A)–C(14)–H(14B)	109.5	H(14A)–C(14)–H(14C)	109.5
H(14B)–C(14)–H(14C)	109.5	H(15A)–C(15)–H(15B)	109.5
H(15A)–C(15)–H(15C)	109.5	H(15A)–C(15)–C(16)	109.5
H(15B)–C(15)–H(15C)	109.5	H(15B)–C(15)–C(16)	109.5

H(15C)–C(15)–C(16)	109.5	C(8)–C(16)–C(15)	114.43(18)
C(8)–C(16)–H(16A)	108.7	C(8)–C(16)–H(16B)	108.7
C(15)–C(16)–H(16A)	108.7	C(15)–C(16)–H(16B)	108.7
H(16A)–C(16)–H(16B)	107.6	C(9)–C(17)–H(17A)	109.5
C(9)–C(17)–H(17B)	109.5	C(9)–C(17)–H(17C)	109.5
H(17A)–C(17)–H(17B)	109.5	H(17A)–C(17)–H(17C)	109.5
H(17B)–C(17)–H(17C)	109.5	C(5)–C(18)–C(19)	120.86(19)
C(5)–C(18)–C(23)	120.1(2)	C(19)–C(18)–C(23)	119.0(2)
C(18)–C(19)–H(19)	119.6	C(18)–C(19)–C(20)	120.8(2)
H(19)–C(19)–C(20)	119.6	C(19)–C(20)–H(20)	120.5
C(19)–C(20)–C(21)	119.0(2)	H(20)–C(20)–C(21)	120.5
Br(1)–C(21)–C(20)	118.40(19)	Br(1)–C(21)–C(22)	120.16(18)
C(20)–C(21)–C(22)	121.4(2)	C(21)–C(22)–H(22)	120.5
C(21)–C(22)–C(23)	119.0(2)	H(22)–C(22)–C(23)	120.5
C(18)–C(23)–C(22)	120.8(2)	C(18)–C(23)–H(23)	119.6
C(22)–C(23)–H(23)	119.6	B(1)–C(24)–C(25)	124.9(2)
B(1)–C(24)–C(29)	118.8(2)	C(25)–C(24)–C(29)	116.29(19)
C(24)–C(25)–H(25)	119.0	C(24)–C(25)–C(26)	122.1(2)
H(25)–C(25)–C(26)	119.0	C(25)–C(26)–H(26)	120.2
C(25)–C(26)–C(27)	119.6(2)	H(26)–C(26)–C(27)	120.2
C(26)–C(27)–H(27)	120.2	C(26)–C(27)–C(28)	119.6(2)
H(27)–C(27)–C(28)	120.2	C(27)–C(28)–H(28)	119.9
C(27)–C(28)–C(29)	120.2(2)	H(28)–C(28)–C(29)	119.9
C(24)–C(29)–C(28)	122.1(2)	C(24)–C(29)–H(29)	119.0
C(28)–C(29)–H(29)	119.0	B(1)–C(30)–C(31)	122.91(17)
B(1)–C(30)–C(35)	122.45(18)	C(31)–C(30)–C(35)	114.63(19)
C(30)–C(31)–H(31)	118.4	C(30)–C(31)–C(32)	123.10(19)
H(31)–C(31)–C(32)	118.4	C(31)–C(32)–H(32)	120.0
C(31)–C(32)–C(33)	120.1(2)	H(32)–C(32)–C(33)	120.0
C(32)–C(33)–H(33)	120.5	C(32)–C(33)–C(34)	119.1(2)
H(33)–C(33)–C(34)	120.5	C(33)–C(34)–H(34)	119.9
C(33)–C(34)–C(35)	120.2(2)	H(34)–C(34)–C(35)	119.9
C(30)–C(35)–C(34)	122.9(2)	C(30)–C(35)–H(35)	118.6
C(34)–C(35)–H(35)	118.6	B(2)–N(3)–C(36)	129.79(16)
B(2)–N(3)–C(39)	119.29(18)	C(36)–N(3)–C(39)	107.64(17)
B(2)–N(4)–C(41)	119.46(17)	B(2)–N(4)–C(44)	129.68(17)
C(41)–N(4)–C(44)	107.01(17)	N(3)–B(2)–N(4)	104.32(15)
N(3)–B(2)–C(59)	107.40(17)	N(3)–B(2)–C(65)	109.8(2)
N(4)–B(2)–C(59)	107.4(2)	N(4)–B(2)–C(65)	111.70(18)
C(59)–B(2)–C(65)	115.57(16)	N(3)–C(36)–C(37)	109.68(19)
N(3)–C(36)–C(45)	125.01(18)	C(37)–C(36)–C(45)	125.3(2)
C(36)–C(37)–C(38)	107.4(2)	C(36)–C(37)–C(47)	126.6(2)
C(38)–C(37)–C(47)	125.99(19)	C(37)–C(38)–C(39)	107.31(18)
C(37)–C(38)–C(48)	123.6(2)	C(39)–C(38)–C(48)	129.0(2)
N(3)–C(39)–C(38)	107.91(19)	N(3)–C(39)–C(40)	119.12(19)
C(38)–C(39)–C(40)	132.79(18)	C(39)–C(40)–C(41)	123.22(18)
C(39)–C(40)–C(53)	116.97(19)	C(41)–C(40)–C(53)	119.8(2)
N(4)–C(41)–C(40)	120.01(19)	N(4)–C(41)–C(42)	108.49(18)
C(40)–C(41)–C(42)	131.48(19)	C(41)–C(42)–C(43)	106.67(19)
C(41)–C(42)–C(49)	128.4(2)	C(43)–C(42)–C(49)	124.9(2)
C(42)–C(43)–C(44)	107.43(19)	C(42)–C(43)–C(51)	127.0(2)
C(44)–C(43)–C(51)	125.5(2)	N(4)–C(44)–C(43)	110.38(18)
N(4)–C(44)–C(52)	124.48(19)	C(43)–C(44)–C(52)	125.1(2)
C(36)–C(45)–H(45A)	109.5	C(36)–C(45)–H(45B)	109.5
C(36)–C(45)–H(45C)	109.5	H(45A)–C(45)–H(45B)	109.5
H(45A)–C(45)–H(45C)	109.5	H(45B)–C(45)–H(45C)	109.5
H(46A)–C(46)–H(46B)	109.5	H(46A)–C(46)–H(46C)	109.5
H(46A)–C(46)–C(47)	109.5	H(46B)–C(46)–H(46C)	109.5
H(46B)–C(46)–C(47)	109.5	H(46C)–C(46)–C(47)	109.5
C(37)–C(47)–C(46)	112.66(19)	C(37)–C(47)–H(47A)	109.1

C(37)–C(47)–H(47B)	109.1	C(46)–C(47)–H(47A)	109.1
C(46)–C(47)–H(47B)	109.1	H(47A)–C(47)–H(47B)	107.8
C(38)–C(48)–H(48A)	109.5	C(38)–C(48)–H(48B)	109.5
C(38)–C(48)–H(48C)	109.5	H(48A)–C(48)–H(48B)	109.5
H(48A)–C(48)–H(48C)	109.5	H(48B)–C(48)–H(48C)	109.5
C(42)–C(49)–H(49A)	109.5	C(42)–C(49)–H(49B)	109.5
C(42)–C(49)–H(49C)	109.5	H(49A)–C(49)–H(49B)	109.5
H(49A)–C(49)–H(49C)	109.5	H(49B)–C(49)–H(49C)	109.5
H(50A)–C(50)–H(50B)	109.5	H(50A)–C(50)–H(50C)	109.5
H(50A)–C(50)–C(51)	109.5	H(50B)–C(50)–H(50C)	109.5
H(50B)–C(50)–C(51)	109.5	H(50C)–C(50)–C(51)	109.5
C(43)–C(51)–C(50)	113.99(19)	C(43)–C(51)–H(51A)	108.8
C(43)–C(51)–H(51B)	108.8	C(50)–C(51)–H(51A)	108.8
C(50)–C(51)–H(51B)	108.8	H(51A)–C(51)–H(51B)	107.7
C(44)–C(52)–H(52A)	109.5	C(44)–C(52)–H(52B)	109.5
C(44)–C(52)–H(52C)	109.5	H(52A)–C(52)–H(52B)	109.5
H(52A)–C(52)–H(52C)	109.5	H(52B)–C(52)–H(52C)	109.5
C(40)–C(53)–C(54)	122.2(2)	C(40)–C(53)–C(58)	118.59(19)
C(54)–C(53)–C(58)	119.19(19)	C(53)–C(54)–H(54)	120.0
C(53)–C(54)–C(55)	119.9(2)	H(54)–C(54)–C(55)	120.0
C(54)–C(55)–H(55)	120.2	C(54)–C(55)–C(56)	119.7(2)
H(55)–C(55)–C(56)	120.2	Br(2)–C(56)–C(55)	119.80(17)
Br(2)–C(56)–C(57)	118.63(19)	C(55)–C(56)–C(57)	121.5(2)
C(56)–C(57)–H(57)	120.7	C(56)–C(57)–C(58)	118.5(2)
H(57)–C(57)–C(58)	120.7	C(53)–C(58)–C(57)	121.2(2)
C(53)–C(58)–H(58)	119.4	C(57)–C(58)–H(58)	119.4
B(2)–C(59)–C(60)	121.1(2)	B(2)–C(59)–C(64)	123.9(2)
C(60)–C(59)–C(64)	115.0(2)	C(59)–C(60)–H(60)	118.8
C(59)–C(60)–C(61)	122.4(2)	H(60)–C(60)–C(61)	118.8
C(60)–C(61)–H(61)	119.7	C(60)–C(61)–C(62)	120.5(2)
H(61)–C(61)–C(62)	119.7	C(61)–C(62)–H(62)	120.8
C(61)–C(62)–C(63)	118.4(2)	H(62)–C(62)–C(63)	120.8
C(62)–C(63)–H(63)	119.8	C(62)–C(63)–C(64)	120.4(3)
H(63)–C(63)–C(64)	119.8	C(59)–C(64)–C(63)	123.3(2)
C(59)–C(64)–H(64)	118.4	C(63)–C(64)–H(64)	118.4
B(2)–C(65)–C(66)	121.40(18)	B(2)–C(65)–C(70)	122.3(2)
C(66)–C(65)–C(70)	116.28(19)	C(65)–C(66)–H(66)	118.8
C(65)–C(66)–C(67)	122.5(2)	H(66)–C(66)–C(67)	118.8
C(66)–C(67)–H(67)	120.5	C(66)–C(67)–C(68)	119.1(2)
H(67)–C(67)–C(68)	120.5	C(67)–C(68)–H(68)	120.0
C(67)–C(68)–C(69)	120.1(2)	H(68)–C(68)–C(69)	120.0
C(68)–C(69)–H(69)	120.0	C(68)–C(69)–C(70)	120.0(2)
H(69)–C(69)–C(70)	120.0	C(65)–C(70)–C(69)	122.1(2)
C(65)–C(70)–H(70)	119.0	C(69)–C(70)–H(70)	119.0

Table 4. Anisotropic displacement parameters (Å^2) for **2a**. The anisotropic displacement factor exponent takes the form: $-2\pi^2[h^2a^{*2}U^{11} + ... + 2hka^*b^*U^{12}]$

	U^{11}	U^{22}	U^{33}	U^{23}	U^{13}	U^{12}
Br(1)	0.0610(2)	0.03800(15)	0.0414(2)	–0.01960(15)	0.01298(15)	–0.00859(13)
N(1)	0.0191(11)	0.0185(10)	0.0187(13)	0.0026(9)	0.0007(9)	0.0003(8)
N(2)	0.0157(10)	0.0202(9)	0.0158(13)	0.0010(9)	–0.0005(9)	–0.0026(8)
B(1)	0.0244(16)	0.0180(13)	0.0207(19)	0.0027(12)	0.0007(14)	–0.0057(11)
C(1)	0.0174(13)	0.0208(12)	0.0266(18)	0.0040(12)	–0.0024(12)	–0.0003(10)
C(2)	0.0143(13)	0.0254(12)	0.0227(17)	0.0080(11)	0.0008(12)	–0.0005(10)
C(3)	0.0124(13)	0.0277(13)	0.0166(16)	–0.0017(11)	–0.0028(11)	–0.0002(10)
C(4)	0.0169(13)	0.0219(12)	0.0160(16)	–0.0016(11)	–0.0045(11)	0.0024(10)
C(5)	0.0132(13)	0.0207(12)	0.0225(17)	–0.0017(11)	–0.0054(11)	0.0034(9)
C(6)	0.0176(13)	0.0175(11)	0.0198(17)	–0.0029(11)	–0.0035(11)	0.0001(10)
C(7)	0.0211(14)	0.0190(12)	0.0246(18)	–0.0002(11)	–0.0035(12)	–0.0006(10)
C(8)	0.0129(13)	0.0208(12)	0.0260(18)	0.0056(11)	–0.0009(11)	–0.0015(10)
C(9)	0.0117(13)	0.0223(12)	0.0207(17)	–0.0002(11)	–0.0023(11)	0.0035(10)
C(10)	0.0317(14)	0.0235(12)	0.0308(18)	0.0010(12)	0.0056(12)	–0.0047(11)
C(11)	0.0388(17)	0.0753(19)	0.047(2)	0.0414(17)	0.0094(16)	0.0090(14)
C(12)	0.0291(15)	0.0325(13)	0.0262(18)	0.0051(12)	0.0034(13)	–0.0014(11)
C(13)	0.0296(15)	0.0342(13)	0.0195(17)	0.0014(12)	0.0009(12)	–0.0036(10)
C(14)	0.0345(15)	0.0240(12)	0.0312(18)	0.0001(12)	–0.0008(13)	–0.0048(10)
C(15)	0.0407(16)	0.0377(14)	0.0356(19)	0.0132(13)	0.0120(14)	0.0035(12)
C(16)	0.0297(15)	0.0244(12)	0.0236(17)	0.0024(12)	0.0034(12)	–0.0011(11)
C(17)	0.0245(14)	0.0273(12)	0.0211(17)	0.0033(11)	0.0045(12)	–0.0052(10)
C(18)	0.0237(14)	0.0186(12)	0.0171(16)	0.0033(11)	0.0024(12)	–0.0041(10)
C(19)	0.0232(14)	0.0232(12)	0.0222(17)	–0.0016(12)	0.0028(12)	0.0006(10)
C(20)	0.0254(15)	0.0328(13)	0.0222(17)	–0.0030(12)	–0.0003(12)	–0.0085(11)
C(21)	0.0388(16)	0.0225(13)	0.0222(18)	–0.0065(11)	0.0109(14)	–0.0093(12)
C(22)	0.0317(15)	0.0198(12)	0.0340(19)	–0.0001(12)	0.0022(14)	0.0026(11)
C(23)	0.0300(15)	0.0225(12)	0.0274(18)	0.0052(12)	–0.0060(13)	–0.0010(11)
C(24)	0.0186(14)	0.0199(11)	0.0168(16)	0.0016(11)	–0.0007(11)	–0.0047(10)
C(25)	0.0269(15)	0.0256(12)	0.0264(18)	0.0008(12)	0.0039(13)	–0.0038(11)
C(26)	0.0398(17)	0.0253(13)	0.0321(19)	–0.0053(12)	0.0103(14)	–0.0066(12)
C(27)	0.0490(19)	0.0243(13)	0.037(2)	0.0005(13)	0.0201(16)	0.0093(13)
C(28)	0.0256(15)	0.0333(14)	0.035(2)	0.0087(13)	0.0088(13)	0.0094(12)
C(29)	0.0325(15)	0.0238(12)	0.0230(17)	–0.0016(11)	0.0026(13)	–0.0029(11)
C(30)	0.0191(13)	0.0194(12)	0.0165(15)	–0.0041(10)	0.0024(11)	–0.0003(10)
C(31)	0.0192(14)	0.0243(13)	0.0298(18)	–0.0033(12)	–0.0005(12)	0.0010(10)
C(32)	0.0281(16)	0.0244(12)	0.043(2)	0.0005(12)	–0.0029(14)	0.0064(12)
C(33)	0.0178(14)	0.0415(15)	0.053(2)	–0.0009(14)	–0.0076(13)	0.0060(12)
C(34)	0.0231(16)	0.0345(15)	0.044(2)	–0.0061(13)	0.0003(14)	–0.0104(11)
C(35)	0.0198(14)	0.0289(12)	0.0297(18)	–0.0019(12)	0.0008(12)	–0.0047(11)
Br(2)	0.05333(19)	0.03767(15)	0.0488(2)	–0.02173(15)	–0.00223(15)	0.01514(13)
N(3)	0.0228(11)	0.0158(9)	0.0193(13)	–0.0010(9)	–0.0012(10)	–0.0004(8)
N(4)	0.0199(11)	0.0162(9)	0.0252(14)	0.0002(9)	0.0004(9)	0.0005(8)
B(2)	0.0207(16)	0.0169(13)	0.025(2)	0.0011(13)	–0.0036(14)	–0.0007(11)
C(36)	0.0281(15)	0.0180(12)	0.0219(17)	0.0029(11)	–0.0029(13)	–0.0033(11)
C(37)	0.0283(15)	0.0226(12)	0.0233(17)	0.0031(11)	–0.0051(13)	0.0014(11)
C(38)	0.0288(15)	0.0204(12)	0.0199(17)	0.0021(11)	0.0026(12)	0.0013(11)
C(39)	0.0255(14)	0.0141(11)	0.0176(16)	0.0005(11)	0.0033(12)	0.0017(10)
C(40)	0.0268(14)	0.0146(11)	0.0139(15)	0.0034(10)	0.0059(12)	–0.0029(11)
C(41)	0.0242(15)	0.0170(12)	0.0179(16)	0.0012(11)	0.0021(12)	–0.0033(11)
C(42)	0.0256(15)	0.0244(12)	0.0203(17)	0.0011(11)	0.0029(12)	–0.0079(11)
C(43)	0.0199(14)	0.0270(13)	0.0214(17)	0.0043(11)	–0.0005(12)	–0.0075(11)
C(44)	0.0231(14)	0.0225(12)	0.0262(17)	0.0056(12)	0.0054(12)	–0.0042(11)

C(45)	0.0370(16)	0.0241(12)	0.0278(19)	–0.0020(12)	–0.0088(13)	–0.0018(10)
C(46)	0.0573(19)	0.0461(15)	0.043(2)	–0.0019(15)	–0.0115(16)	0.0105(14)
C(47)	0.0407(16)	0.0257(13)	0.0347(19)	–0.0026(13)	–0.0108(14)	0.0077(11)
C(48)	0.0376(16)	0.0272(12)	0.0319(19)	–0.0004(12)	–0.0069(13)	0.0102(11)
C(49)	0.0340(15)	0.0295(13)	0.043(2)	–0.0068(13)	–0.0011(14)	–0.0095(11)
C(50)	0.0458(19)	0.0751(19)	0.046(2)	0.0079(17)	–0.0187(16)	–0.0117(14)
C(51)	0.0277(16)	0.0378(14)	0.040(2)	–0.0011(14)	0.0006(14)	–0.0078(11)
C(52)	0.0276(15)	0.0302(13)	0.040(2)	–0.0003(12)	0.0019(13)	0.0043(11)
C(53)	0.0206(14)	0.0187(12)	0.0243(18)	–0.0047(11)	0.0004(12)	–0.0015(10)
C(54)	0.0439(16)	0.0205(12)	0.0307(18)	–0.0026(12)	0.0125(13)	0.0000(11)
C(55)	0.0520(17)	0.0193(12)	0.0306(19)	–0.0040(12)	0.0039(14)	–0.0019(12)
C(56)	0.0346(16)	0.0259(14)	0.0314(19)	–0.0142(12)	–0.0025(13)	0.0089(11)
C(57)	0.0288(15)	0.0341(14)	0.0256(18)	–0.0054(13)	–0.0023(12)	0.0027(11)
C(58)	0.0277(15)	0.0217(12)	0.0248(18)	–0.0021(11)	–0.0037(13)	–0.0032(10)
C(59)	0.0208(14)	0.0122(11)	0.0201(17)	–0.0042(10)	–0.0041(12)	0.0029(9)
C(60)	0.0329(16)	0.0425(15)	0.0228(18)	0.0064(13)	–0.0055(13)	–0.0086(12)
C(61)	0.0337(16)	0.0474(16)	0.034(2)	0.0089(14)	0.0019(15)	–0.0141(12)
C(62)	0.0448(18)	0.0339(14)	0.0217(19)	0.0066(12)	0.0126(15)	–0.0047(12)
C(63)	0.0474(18)	0.0384(14)	0.0187(18)	0.0074(13)	–0.0009(14)	–0.0016(13)
C(64)	0.0254(15)	0.0282(13)	0.0270(19)	–0.0018(13)	0.0010(14)	–0.0054(11)
C(65)	0.0193(13)	0.0205(12)	0.0167(16)	0.0001(11)	–0.0039(11)	0.0013(10)
C(66)	0.0339(15)	0.0238(12)	0.0290(19)	–0.0014(12)	0.0004(14)	–0.0012(11)
C(67)	0.0386(17)	0.0390(15)	0.0274(19)	–0.0016(13)	0.0131(14)	0.0004(12)
C(68)	0.0360(16)	0.0297(14)	0.0295(19)	–0.0110(13)	0.0022(14)	0.0068(11)
C(69)	0.0423(16)	0.0180(12)	0.0328(19)	–0.0021(12)	–0.0008(14)	0.0056(11)
C(70)	0.0292(14)	0.0205(12)	0.0251(17)	–0.0013(11)	0.0023(12)	0.0029(10)

Table 5. Hydrogen coordinates and isotropic displacement parameters (Å^2) for **2a**.

	x	y	z	U
H(10A)	0.7287	–0.0873	0.3491	0.043
H(10B)	0.5789	–0.0914	0.3890	0.043
H(10C)	0.7268	–0.1078	0.4227	0.043
H(11A)	0.6859	–0.0964	0.6269	0.081
H(11B)	0.7821	–0.0550	0.6132	0.081
H(11C)	0.8040	–0.0966	0.5682	0.081
H(12A)	0.5748	–0.0879	0.5199	0.035
H(12B)	0.5416	–0.0487	0.5685	0.035
H(13A)	0.6231	0.0156	0.5943	0.042
H(13B)	0.6413	0.0600	0.5559	0.042
H(13C)	0.7804	0.0367	0.5875	0.042
H(14A)	1.0445	0.1671	0.3361	0.045
H(14B)	1.0151	0.1463	0.4081	0.045
H(14C)	0.8834	0.1663	0.3658	0.045
H(15A)	1.0465	0.1736	0.1500	0.057
H(15B)	0.9314	0.1748	0.2099	0.057
H(15C)	0.9140	0.1407	0.1508	0.057
H(16A)	1.1283	0.1063	0.1864	0.031
H(16B)	1.1508	0.1410	0.2441	0.031
H(17A)	0.8586	0.0220	0.1749	0.036
H(17B)	1.0204	0.0075	0.1939	0.036
H(17C)	0.9938	0.0509	0.1538	0.036
H(19)	1.0112	0.0784	0.5172	0.027
H(20)	1.0138	0.1301	0.6015	0.032
H(22)	0.6371	0.1781	0.5292	0.034
H(23)	0.6395	0.1278	0.4430	0.032
H(25)	0.7171	–0.0589	0.2075	0.032
H(26)	0.8452	–0.1174	0.1670	0.039
H(27)	1.0695	–0.1353	0.2147	0.044
H(28)	1.1694	–0.0924	0.2974	0.038
H(29)	1.0393	–0.0350	0.3393	0.032
H(31)	0.6660	0.0732	0.2563	0.029
H(32)	0.4460	0.0907	0.2074	0.038
H(33)	0.2637	0.0399	0.1972	0.045
H(34)	0.3026	–0.0281	0.2404	0.041
H(35)	0.5217	–0.0452	0.2907	0.031
H(45A)	0.2164	0.2210	0.3180	0.045
H(45B)	0.2901	0.2443	0.2553	0.045
H(45C)	0.1169	0.2428	0.2615	0.045
H(46A)	–0.1257	0.3417	0.1980	0.073
H(46B)	0.0469	0.3361	0.1944	0.073
H(46C)	–0.0218	0.3737	0.2381	0.073
H(47A)	–0.0561	0.2858	0.2684	0.041
H(47B)	–0.1210	0.3237	0.3126	0.041
H(48A)	–0.0453	0.3845	0.3638	0.048
H(48B)	0.0975	0.4121	0.3502	0.048
H(48C)	0.0592	0.3974	0.4250	0.048
H(49A)	0.7263	0.4060	0.5498	0.053
H(49B)	0.5534	0.4048	0.5570	0.053
H(49C)	0.6256	0.4269	0.4929	0.053
H(50A)	1.0003	0.3243	0.6039	0.084
H(50B)	0.8733	0.2911	0.5873	0.084
H(50C)	0.8344	0.3374	0.6155	0.084
H(51A)	0.9196	0.3685	0.5157	0.042

H(51B)	0.9639	0.3228	0.4876	0.042
H(52A)	0.7279	0.2344	0.4453	0.049
H(52B)	0.8744	0.2605	0.4588	0.049
H(52C)	0.8074	0.2580	0.3845	0.049
H(54)	0.3943	0.4404	0.3997	0.038
H(55)	0.2992	0.5011	0.4514	0.041
H(57)	0.1389	0.4290	0.6014	0.035
H(58)	0.2346	0.3685	0.5501	0.030
H(60)	0.1774	0.2338	0.4291	0.039
H(61)	0.0742	0.2045	0.5241	0.046
H(62)	0.2022	0.2031	0.6255	0.040
H(63)	0.4345	0.2326	0.6297	0.042
H(64)	0.5358	0.2623	0.5349	0.032
H(66)	0.5849	0.2795	0.2830	0.035
H(67)	0.6759	0.2313	0.2053	0.042
H(68)	0.6513	0.1584	0.2250	0.038
H(69)	0.5420	0.1346	0.3225	0.037
H(70)	0.4533	0.1830	0.4002	0.030

Table 6. Torsion angles [°] for **2a**.

C(6)–N(2)–B(1)–N(1)	−29.8(2)	C(6)–N(2)–B(1)–C(24)	−146.71(19)
C(6)–N(2)–B(1)–C(30)	86.5(2)	C(9)–N(2)–B(1)–N(1)	168.19(17)
C(9)–N(2)–B(1)–C(24)	51.3(3)	C(9)–N(2)–B(1)–C(30)	−75.5(2)
C(1)–N(1)–B(1)–N(2)	−165.34(18)	C(1)–N(1)–B(1)–C(24)	−48.8(3)
C(1)–N(1)–B(1)–C(30)	80.3(2)	C(4)–N(1)–B(1)–N(2)	28.7(2)
C(4)–N(1)–B(1)–C(24)	145.33(17)	C(4)–N(1)–B(1)–C(30)	−85.6(2)
B(1)–N(1)–C(1)–C(2)	−166.50(19)	B(1)–N(1)–C(1)–C(10)	11.9(3)
C(4)–N(1)–C(1)–C(2)	1.2(2)	C(4)–N(1)–C(1)–C(10)	179.60(19)
N(1)–C(1)–C(2)–C(3)	−0.2(3)	N(1)–C(1)–C(2)–C(12)	179.86(18)
C(10)–C(1)–C(2)–C(3)	−178.66(18)	C(10)–C(1)–C(2)–C(12)	1.4(3)
C(1)–C(2)–C(3)–C(4)	−0.8(2)	C(1)–C(2)–C(3)–C(13)	177.9(2)
C(12)–C(2)–C(3)–C(4)	179.1(2)	C(12)–C(2)–C(3)–C(13)	−2.2(3)
B(1)–N(1)–C(4)–C(3)	167.07(18)	B(1)–N(1)–C(4)–C(5)	−15.8(3)
C(1)–N(1)–C(4)–C(3)	−1.7(2)	C(1)–N(1)–C(4)–C(5)	175.48(18)
C(2)–C(3)–C(4)–N(1)	1.6(2)	C(2)–C(3)–C(4)–C(5)	−175.2(2)
C(13)–C(3)–C(4)–N(1)	−177.1(2)	C(13)–C(3)–C(4)–C(5)	6.2(4)
N(1)–C(4)–C(5)–C(6)	−2.2(3)	N(1)–C(4)–C(5)–C(18)	−178.82(17)
C(3)–C(4)–C(5)–C(6)	174.2(2)	C(3)–C(4)–C(5)–C(18)	−2.4(3)
C(4)–C(5)–C(6)–N(2)	1.5(3)	C(4)–C(5)–C(6)–C(7)	−175.4(2)
C(18)–C(5)–C(6)–N(2)	177.98(17)	C(18)–C(5)–C(6)–C(7)	1.1(4)
B(1)–N(2)–C(6)–C(5)	17.5(3)	B(1)–N(2)–C(6)–C(7)	−164.96(18)
C(9)–N(2)–C(6)–C(5)	−177.63(19)	C(9)–N(2)–C(6)–C(7)	−0.1(2)
N(2)–C(6)–C(7)–C(8)	−1.1(2)	N(2)–C(6)–C(7)–C(14)	179.38(19)
C(5)–C(6)–C(7)–C(8)	176.1(2)	C(5)–C(6)–C(7)–C(14)	−3.5(4)
C(6)–C(7)–C(8)–C(9)	1.8(2)	C(6)–C(7)–C(8)–C(16)	−178.09(19)
C(14)–C(7)–C(8)–C(9)	−178.68(18)	C(14)–C(7)–C(8)–C(16)	1.5(4)
B(1)–N(2)–C(9)–C(8)	165.40(18)	B(1)–N(2)–C(9)–C(17)	−10.3(3)
C(6)–N(2)–C(9)–C(8)	1.2(2)	C(6)–N(2)–C(9)–C(17)	−174.53(19)
C(7)–C(8)–C(9)–N(2)	−1.9(2)	C(7)–C(8)–C(9)–C(17)	173.7(2)
C(16)–C(8)–C(9)–N(2)	178.00(19)	C(16)–C(8)–C(9)–C(17)	−6.4(3)
C(1)–C(2)–C(12)–C(11)	102.3(3)	C(3)–C(2)–C(12)–C(11)	−77.6(3)
C(7)–C(8)–C(16)–C(15)	−82.5(3)	C(9)–C(8)–C(16)–C(15)	97.7(2)
C(4)–C(5)–C(18)–C(19)	82.0(3)	C(4)–C(5)–C(18)–C(23)	−96.8(2)
C(6)–C(5)–C(18)–C(19)	−94.7(3)	C(6)–C(5)–C(18)–C(23)	86.5(2)
C(5)–C(18)–C(19)–C(20)	−178.0(2)	C(23)–C(18)–C(19)–C(20)	0.8(3)
C(18)–C(19)–C(20)–C(21)	0.3(3)	C(19)–C(20)–C(21)–Br(1)	179.90(16)
C(19)–C(20)–C(21)–C(22)	−0.3(3)	Br(1)–C(21)–C(22)–C(23)	178.96(16)
C(20)–C(21)–C(22)–C(23)	−0.8(3)	C(21)–C(22)–C(23)–C(18)	2.0(3)
C(5)–C(18)–C(23)–C(22)	176.8(2)	C(19)–C(18)–C(23)–C(22)	−2.0(3)
N(1)–B(1)–C(24)–C(25)	122.0(2)	N(1)–B(1)–C(24)–C(29)	−57.4(2)
N(2)–B(1)–C(24)–C(25)	−124.7(2)	N(2)–B(1)–C(24)–C(29)	55.9(3)
C(30)–B(1)–C(24)–C(25)	−3.3(3)	C(30)–B(1)–C(24)–C(29)	177.21(19)
B(1)–C(24)–C(25)–C(26)	−177.6(2)	C(29)–C(24)–C(25)–C(26)	1.9(3)
C(24)–C(25)–C(26)–C(27)	−0.3(3)	C(25)–C(26)–C(27)–C(28)	−2.0(3)
C(26)–C(27)–C(28)–C(29)	2.8(4)	B(1)–C(24)–C(29)–C(28)	178.33(19)
C(25)–C(24)–C(29)–C(28)	−1.2(3)	C(27)–C(28)–C(29)–C(24)	−1.1(3)
N(1)–B(1)–C(30)–C(31)	111.3(2)	N(1)–B(1)–C(30)–C(35)	−67.9(3)
N(2)–B(1)–C(30)–C(31)	−1.1(3)	N(2)–B(1)–C(30)–C(35)	179.69(19)
C(24)–B(1)–C(30)–C(31)	−123.4(2)	C(24)–B(1)–C(30)–C(35)	57.3(3)
B(1)–C(30)–C(31)–C(32)	179.4(2)	C(35)–C(30)–C(31)–C(32)	−1.3(3)
C(30)–C(31)–C(32)–C(33)	−0.2(4)	C(31)–C(32)–C(33)–C(34)	1.3(4)
C(32)–C(33)–C(34)–C(35)	−0.8(4)	C(33)–C(34)–C(35)–C(30)	−0.8(4)
B(1)–C(30)–C(35)–C(34)	−178.9(2)	C(31)–C(30)–C(35)–C(34)	1.8(3)
C(41)–N(4)–B(2)–N(3)	39.9(3)	C(41)–N(4)–B(2)–C(59)	−73.8(2)
C(41)–N(4)–B(2)–C(65)	158.49(19)	C(44)–N(4)–B(2)–N(3)	−165.2(2)

C(44)–N(4)–B(2)–C(59)	81.0(3)	C(44)–N(4)–B(2)–C(65)	−46.7(3)
C(36)–N(3)–B(2)–N(4)	162.9(2)	C(36)–N(3)–B(2)–C(59)	−83.4(3)
C(36)–N(3)–B(2)–C(65)	43.1(3)	C(39)–N(3)–B(2)–N(4)	−40.3(2)
C(39)–N(3)–B(2)–C(59)	73.5(2)	C(39)–N(3)–B(2)–C(65)	−160.10(17)
B(2)–N(3)–C(36)–C(37)	159.4(2)	B(2)–N(3)–C(36)–C(45)	−19.3(4)
C(39)–N(3)–C(36)–C(37)	0.5(2)	C(39)–N(3)–C(36)–C(45)	−178.2(2)
N(3)–C(36)–C(37)–C(38)	0.1(3)	N(3)–C(36)–C(37)–C(47)	179.3(2)
C(45)–C(36)–C(37)–C(38)	178.8(2)	C(45)–C(36)–C(37)–C(47)	−2.1(4)
C(36)–C(37)–C(38)–C(39)	−0.6(3)	C(36)–C(37)–C(38)–C(48)	176.6(2)
C(47)–C(37)–C(38)–C(39)	−179.8(2)	C(47)–C(37)–C(38)–C(48)	−2.6(4)
B(2)–N(3)–C(39)–C(38)	−162.38(18)	B(2)–N(3)–C(39)–C(40)	21.8(3)
C(36)–N(3)–C(39)–C(38)	−0.9(2)	C(36)–N(3)–C(39)–C(40)	−176.71(19)
C(37)–C(38)–C(39)–N(3)	0.9(2)	C(37)–C(38)–C(39)–C(40)	176.0(2)
C(48)–C(38)–C(39)–N(3)	−176.1(2)	C(48)–C(38)–C(39)–C(40)	−1.0(4)
N(3)–C(39)–C(40)–C(41)	3.5(3)	N(3)–C(39)–C(40)–C(53)	−175.39(18)
C(38)–C(39)–C(40)–C(41)	−171.1(2)	C(38)–C(39)–C(40)–C(53)	10.0(4)
C(39)–C(40)–C(41)–N(4)	−3.9(3)	C(39)–C(40)–C(41)–C(42)	174.1(2)
C(53)–C(40)–C(41)–N(4)	174.96(18)	C(53)–C(40)–C(41)–C(42)	−7.0(4)
B(2)–N(4)–C(41)–C(40)	−21.2(3)	B(2)–N(4)–C(41)–C(42)	160.32(19)
C(44)–N(4)–C(41)–C(40)	178.78(19)	C(44)–N(4)–C(41)–C(42)	0.3(2)
N(4)–C(41)–C(42)–C(43)	0.8(2)	N(4)–C(41)–C(42)–C(49)	179.7(2)
C(40)–C(41)–C(42)–C(43)	−177.4(2)	C(40)–C(41)–C(42)–C(49)	1.5(4)
C(41)–C(42)–C(43)–C(44)	−1.5(2)	C(41)–C(42)–C(43)–C(51)	−178.5(2)
C(49)–C(42)–C(43)–C(44)	179.5(2)	C(49)–C(42)–C(43)–C(51)	2.5(4)
B(2)–N(4)–C(44)–C(43)	−158.5(2)	B(2)–N(4)–C(44)–C(52)	19.0(4)
C(41)–N(4)–C(44)–C(43)	−1.3(2)	C(41)–N(4)–C(44)–C(52)	176.2(2)
C(42)–C(43)–C(44)–N(4)	1.8(3)	C(42)–C(43)–C(44)–C(52)	−175.7(2)
C(51)–C(43)–C(44)–N(4)	178.9(2)	C(51)–C(43)–C(44)–C(52)	1.3(4)
C(36)–C(37)–C(47)–C(46)	−103.4(3)	C(38)–C(37)–C(47)–C(46)	75.6(3)
C(42)–C(43)–C(51)–C(50)	86.1(3)	C(44)–C(43)–C(51)–C(50)	−90.4(3)
C(39)–C(40)–C(53)–C(54)	−90.8(3)	C(39)–C(40)–C(53)–C(58)	86.6(3)
C(41)–C(40)–C(53)–C(54)	90.2(3)	C(41)–C(40)–C(53)–C(58)	−92.4(3)
C(40)–C(53)–C(54)–C(55)	177.1(2)	C(58)–C(53)–C(54)–C(55)	−0.3(3)
C(53)–C(54)–C(55)–C(56)	0.0(4)	C(54)–C(55)–C(56)–Br(2)	−178.02(18)
C(54)–C(55)–C(56)–C(57)	0.0(4)	Br(2)–C(56)–C(57)–C(58)	178.19(17)
C(55)–C(56)–C(57)–C(58)	0.1(4)	C(56)–C(57)–C(58)–C(53)	−0.3(3)
C(40)–C(53)–C(58)–C(57)	−177.1(2)	C(54)–C(53)–C(58)–C(57)	0.4(3)
N(3)–B(2)–C(59)–C(60)	42.7(2)	N(3)–B(2)–C(59)–C(64)	−135.7(2)
N(4)–B(2)–C(59)–C(60)	154.42(17)	N(4)–B(2)–C(59)–C(64)	−24.0(3)
C(65)–B(2)–C(59)–C(60)	−80.2(3)	C(65)–B(2)–C(59)–C(64)	101.4(2)
B(2)–C(59)–C(60)–C(61)	179.81(19)	C(64)–C(59)–C(60)–C(61)	−1.6(3)
C(59)–C(60)–C(61)–C(62)	0.4(4)	C(60)–C(61)–C(62)–C(63)	0.7(3)
C(61)–C(62)–C(63)–C(64)	−0.4(3)	B(2)–C(59)–C(64)–C(63)	−179.55(19)
C(60)–C(59)–C(64)–C(63)	2.0(3)	C(62)–C(63)–C(64)–C(59)	−1.0(3)
N(3)–B(2)–C(65)–C(66)	60.7(3)	N(3)–B(2)–C(65)–C(70)	−117.9(2)
N(4)–B(2)–C(65)–C(66)	−54.5(3)	N(4)–B(2)–C(65)–C(70)	126.8(2)
C(59)–B(2)–C(65)–C(66)	−177.6(2)	C(59)–B(2)–C(65)–C(70)	3.7(3)
B(2)–C(65)–C(66)–C(67)	−178.3(2)	C(70)–C(65)–C(66)–C(67)	0.5(3)
C(65)–C(66)–C(67)–C(68)	0.3(4)	C(66)–C(67)–C(68)–C(69)	−0.8(4)
C(67)–C(68)–C(69)–C(70)	0.5(4)	C(68)–C(69)–C(70)–C(65)	0.3(4)
B(2)–C(65)–C(70)–C(69)	178.0(2)	C(66)–C(65)–C(70)–C(69)	−0.8(3)

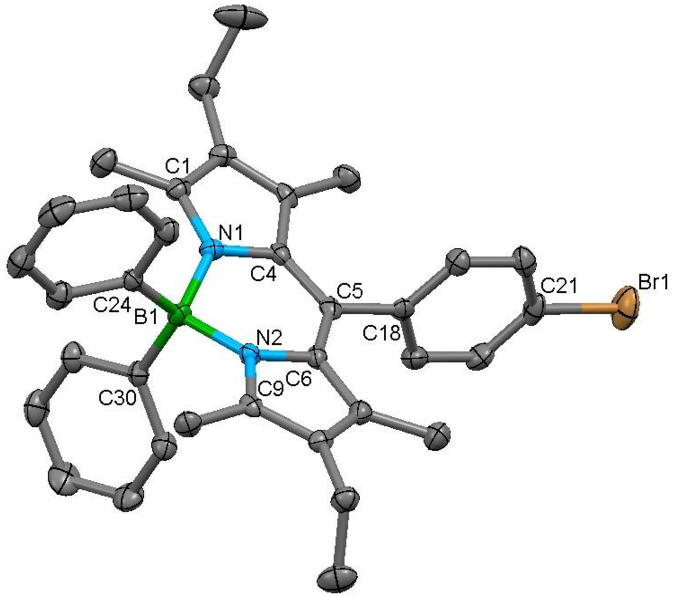

Figure S1. View of the molecular structure of one of the two crystallographically independent molecules of **2a** with 50% probability displacement ellipsoids. Hydrogen atoms have been omitted for clarity.

3.3 Crystallographic data for **2b**

Table 7. Crystal data and structure refinement for **2b**.

Identification code	2b	
Chemical formula (moiety)	$C_{25}H_{32}BBrN_2$	
Chemical formula (total)	$C_{25}H_{32}BBrN_2$	
Formula weight	451.25	
Temperature	120(2) K	
Radiation, wavelength	synchrotron, 0.6889 Å	
Crystal system, space group	monoclinic, $P2_1/n$	
Unit cell parameters	a = 7.9405(19) Å	α = 90°
	b = 11.056(3) Å	β = 90.180(2)°
	c = 25.950(6) Å	γ = 90°
Cell volume	2278.1(10) $Å^3$	
Z	4	
Calculated density	1.316 g/cm^3	
Absorption coefficient μ	1.685 mm^{-1}	
F(000)	944	
Crystal colour and size	red, 0.04 × 0.03 × 0.02 mm^3	
Reflections for cell refinement	9947 (θ range 2.9 to 25.6°)	
Data collection method	Crystal Logic diffractometer and Rigaku Saturn 724+ CCD thick-slice ω scans	
θ range for data collection	0.8 to 25.6°	
Index ranges	h –9 to 9, k –13 to 13, l –32 to 32	
Completeness to θ = 25.6°	97.8 %	
Reflections collected	19429	
Independent reflections	4574 (R_{int} = 0.0563)	
Reflections with $F^2>2\sigma$	3992	
Absorption correction	semi-empirical from equivalents	
Min. and max. transmission	0.936 and 0.967	
Structure solution	direct methods	
Refinement method	Full-matrix least-squares on F^2	
Weighting parameters a, b	0.0438, 1.6969	
Data / restraints / parameters	4574 / 0 / 271	
Final R indices [$F^2>2\sigma$]	R1 = 0.0423, wR2 = 0.1045	
R indices (all data)	R1 = 0.0500, wR2 = 0.1099	
Goodness-of-fit on F^2	1.126	
Largest and mean shift/su	0.002 and 0.000	
Largest diff. peak and hole	1.20 and –0.91 e $Å^{-3}$	

Table 8. Atomic coordinates and equivalent isotropic displacement parameters (Å^2) for **2b**. U_{eq} is defined as one third of the trace of the orthogonalized U^{ij} tensor.

	x	y	z	U_{eq}
Br	0.42229(7)	0.17677(3)	0.668290(14)	0.04939(15)
B	0.5634(5)	0.3088(3)	0.32122(12)	0.0247(7)
N(1)	0.4154(4)	0.3597(2)	0.35664(9)	0.0235(5)
N(2)	0.6977(3)	0.2548(2)	0.36033(9)	0.0215(5)
C(1)	0.2767(4)	0.4191(3)	0.34099(12)	0.0269(7)
C(2)	0.1627(4)	0.4355(3)	0.38294(13)	0.0269(7)
C(3)	0.2371(4)	0.3845(3)	0.42588(11)	0.0227(6)
C(4)	0.3964(4)	0.3380(3)	0.40960(11)	0.0213(6)
C(5)	0.5240(4)	0.2802(2)	0.43679(11)	0.0191(6)
C(6)	0.6717(4)	0.2398(3)	0.41371(11)	0.0203(6)
C(7)	0.8159(4)	0.1792(3)	0.43450(11)	0.0217(6)
C(8)	0.9248(4)	0.1578(2)	0.39359(11)	0.0228(6)
C(9)	0.8473(4)	0.2052(3)	0.34849(12)	0.0232(6)
C(10)	0.2503(5)	0.4640(3)	0.28722(13)	0.0354(8)
C(11)	0.0096(5)	0.6359(3)	0.38531(17)	0.0415(9)
C(12)	–0.0033(5)	0.4990(3)	0.37981(14)	0.0329(7)
C(13)	0.1581(4)	0.3794(3)	0.47873(12)	0.0274(7)
C(14)	0.8497(4)	0.1446(3)	0.48933(12)	0.0240(6)
C(15)	1.0789(5)	–0.0396(3)	0.38003(13)	0.0352(7)
C(16)	1.0918(4)	0.0947(3)	0.39373(12)	0.0278(7)
C(17)	0.9233(5)	0.1989(3)	0.29567(11)	0.0300(7)
C(18)	0.4982(4)	0.2573(3)	0.49338(11)	0.0204(6)
C(19)	0.4296(4)	0.1481(3)	0.50962(11)	0.0235(6)
C(20)	0.4065(5)	0.1237(3)	0.56178(11)	0.0282(7)
C(21)	0.4524(4)	0.2109(3)	0.59708(11)	0.0293(7)
C(22)	0.5198(5)	0.3201(3)	0.58221(13)	0.0308(7)
C(23)	0.5428(4)	0.3428(3)	0.52994(12)	0.0268(7)
C(24)	0.6462(5)	0.4184(3)	0.28799(13)	0.0326(8)
C(25)	0.4887(5)	0.1965(3)	0.28776(14)	0.0357(8)

Table 9. Bond lengths [Å] and angles [°] for **2b**.

Br–C(21)	1.902(3)	B–N(1)	1.597(4)
B–N(2)	1.587(4)	B–C(24)	1.628(5)
B–C(25)	1.626(5)	N(1)–C(1)	1.344(4)
N(1)–C(4)	1.404(4)	N(2)–C(6)	1.411(4)
N(2)–C(9)	1.344(4)	C(1)–C(2)	1.429(5)
C(1)–C(10)	1.495(4)	C(2)–C(3)	1.380(5)
C(2)–C(12)	1.495(5)	C(3)–C(4)	1.430(4)
C(3)–C(13)	1.511(4)	C(4)–C(5)	1.389(4)
C(5)–C(6)	1.392(4)	C(5)–C(18)	1.505(4)
C(6)–C(7)	1.430(4)	C(7)–C(8)	1.392(4)
C(7)–C(14)	1.497(4)	C(8)–C(9)	1.421(4)
C(8)–C(16)	1.499(5)	C(9)–C(17)	1.501(4)
C(10)–H(10A)	0.980	C(10)–H(10B)	0.980
C(10)–H(10C)	0.980	C(11)–H(11A)	0.980
C(11)–H(11B)	0.980	C(11)–H(11C)	0.980
C(11)–C(12)	1.523(5)	C(12)–H(12A)	0.990
C(12)–H(12B)	0.990	C(13)–H(13A)	0.980
C(13)–H(13B)	0.980	C(13)–H(13C)	0.980
C(14)–H(14A)	0.980	C(14)–H(14B)	0.980
C(14)–H(14C)	0.980	C(15)–H(15A)	0.980
C(15)–H(15B)	0.980	C(15)–H(15C)	0.980
C(15)–C(16)	1.530(4)	C(16)–H(16A)	0.990
C(16)–H(16B)	0.990	C(17)–H(17A)	0.980
C(17)–H(17B)	0.980	C(17)–H(17C)	0.980
C(18)–C(19)	1.390(4)	C(18)–C(23)	1.385(4)
C(19)–H(19)	0.950	C(19)–C(20)	1.393(4)
C(20)–H(20)	0.950	C(20)–C(21)	1.378(5)
C(21)–C(22)	1.376(5)	C(22)–H(22)	0.950
C(22)–C(23)	1.392(4)	C(23)–H(23)	0.950
C(24)–H(24A)	0.980	C(24)–H(24B)	0.980
C(24)–H(24C)	0.980	C(25)–H(25A)	0.980
C(25)–H(25B)	0.980	C(25)–H(25C)	0.980
N(1)–B–N(2)	105.0(2)	N(1)–B–C(24)	110.0(3)
N(1)–B–C(25)	108.0(3)	N(2)–B–C(24)	110.3(3)
N(2)–B–C(25)	107.3(3)	C(24)–B–C(25)	115.7(3)
B–N(1)–C(1)	127.0(2)	B–N(1)–C(4)	125.8(2)
C(1)–N(1)–C(4)	106.8(3)	B–N(2)–C(6)	124.9(3)
B–N(2)–C(9)	126.8(2)	C(6)–N(2)–C(9)	108.0(2)
N(1)–C(1)–C(2)	110.6(3)	N(1)–C(1)–C(10)	123.8(3)
C(2)–C(1)–C(10)	125.6(3)	C(1)–C(2)–C(3)	107.0(3)
C(1)–C(2)–C(12)	125.3(3)	C(3)–C(2)–C(12)	127.6(3)
C(2)–C(3)–C(4)	106.6(3)	C(2)–C(3)–C(13)	124.8(3)
C(4)–C(3)–C(13)	128.7(3)	N(1)–C(4)–C(3)	109.0(3)
N(1)–C(4)–C(5)	119.7(3)	C(3)–C(4)–C(5)	131.3(3)
C(4)–C(5)–C(6)	122.9(3)	C(4)–C(5)–C(18)	118.2(3)
C(6)–C(5)–C(18)	118.9(3)	N(2)–C(6)–C(5)	120.7(3)
N(2)–C(6)–C(7)	107.8(3)	C(5)–C(6)–C(7)	131.5(3)
C(6)–C(7)–C(8)	106.9(3)	C(6)–C(7)–C(14)	128.2(3)
C(8)–C(7)–C(14)	124.9(3)	C(7)–C(8)–C(9)	107.3(3)
C(7)–C(8)–C(16)	129.0(3)	C(9)–C(8)–C(16)	123.7(3)
N(2)–C(9)–C(8)	110.0(3)	N(2)–C(9)–C(17)	125.9(3)
C(8)–C(9)–C(17)	124.1(3)	C(1)–C(10)–H(10A)	109.5
C(1)–C(10)–H(10B)	109.5	C(1)–C(10)–H(10C)	109.5
H(10A)–C(10)–H(10B)	109.5	H(10A)–C(10)–H(10C)	109.5
H(10B)–C(10)–H(10C)	109.5	H(11A)–C(11)–H(11B)	109.5
H(11A)–C(11)–H(11C)	109.5	H(11A)–C(11)–C(12)	109.5
H(11B)–C(11)–H(11C)	109.5	H(11B)–C(11)–C(12)	109.5

H(11C)–C(11)–C(12)	109.5	C(2)–C(12)–C(11)	113.7(3)
C(2)–C(12)–H(12A)	108.8	C(2)–C(12)–H(12B)	108.8
C(11)–C(12)–H(12A)	108.8	C(11)–C(12)–H(12B)	108.8
H(12A)–C(12)–H(12B)	107.7	C(3)–C(13)–H(13A)	109.5
C(3)–C(13)–H(13B)	109.5	C(3)–C(13)–H(13C)	109.5
H(13A)–C(13)–H(13B)	109.5	H(13A)–C(13)–H(13C)	109.5
H(13B)–C(13)–H(13C)	109.5	C(7)–C(14)–H(14A)	109.5
C(7)–C(14)–H(14B)	109.5	C(7)–C(14)–H(14C)	109.5
H(14A)–C(14)–H(14B)	109.5	H(14A)–C(14)–H(14C)	109.5
H(14B)–C(14)–H(14C)	109.5	H(15A)–C(15)–H(15B)	109.5
H(15A)–C(15)–H(15C)	109.5	H(15A)–C(15)–C(16)	109.5
H(15B)–C(15)–H(15C)	109.5	H(15B)–C(15)–C(16)	109.5
H(15C)–C(15)–C(16)	109.5	C(8)–C(16)–C(15)	113.1(3)
C(8)–C(16)–H(16A)	109.0	C(8)–C(16)–H(16B)	109.0
C(15)–C(16)–H(16A)	109.0	C(15)–C(16)–H(16B)	109.0
H(16A)–C(16)–H(16B)	107.8	C(9)–C(17)–H(17A)	109.5
C(9)–C(17)–H(17B)	109.5	C(9)–C(17)–H(17C)	109.5
H(17A)–C(17)–H(17B)	109.5	H(17A)–C(17)–H(17C)	109.5
H(17B)–C(17)–H(17C)	109.5	C(5)–C(18)–C(19)	119.8(3)
C(5)–C(18)–C(23)	121.2(3)	C(19)–C(18)–C(23)	119.0(3)
C(18)–C(19)–H(19)	119.5	C(18)–C(19)–C(20)	121.0(3)
H(19)–C(19)–C(20)	119.5	C(19)–C(20)–H(20)	120.8
C(19)–C(20)–C(21)	118.4(3)	H(20)–C(20)–C(21)	120.8
Br–C(21)–C(20)	118.3(2)	Br–C(21)–C(22)	119.8(2)
C(20)–C(21)–C(22)	121.9(3)	C(21)–C(22)–H(22)	120.5
C(21)–C(22)–C(23)	118.9(3)	H(22)–C(22)–C(23)	120.5
C(18)–C(23)–C(22)	120.7(3)	C(18)–C(23)–H(23)	119.6
C(22)–C(23)–H(23)	119.6	B–C(24)–H(24A)	109.5
B–C(24)–H(24B)	109.5	B–C(24)–H(24C)	109.5
H(24A)–C(24)–H(24B)	109.5	H(24A)–C(24)–H(24C)	109.5
H(24B)–C(24)–H(24C)	109.5	B–C(25)–H(25A)	109.5
B–C(25)–H(25B)	109.5	B–C(25)–H(25C)	109.5
H(25A)–C(25)–H(25B)	109.5	H(25A)–C(25)–H(25C)	109.5
H(25B)–C(25)–H(25C)	109.5		

Table 10. Anisotropic displacement parameters ($Å^2$) for **2b**. The anisotropic displacement factor exponent takes the form: $-2\pi^2[h^2a^{*2}U^{11} + ... + 2hka^*b^*U^{12}]$

	U^{11}	U^{22}	U^{33}	U^{23}	U^{13}	U^{12}
Br	0.0755(3)	0.0559(2)	0.01690(16)	0.00503(15)	0.00963(19)	0.0107(2)
B	0.0305(18)	0.0257(16)	0.0178(16)	–0.0004(12)	0.0015(14)	0.0012(15)
N(1)	0.0319(14)	0.0188(12)	0.0198(11)	0.0023(9)	–0.0014(11)	0.0026(12)
N(2)	0.0290(14)	0.0202(12)	0.0153(11)	0.0004(9)	0.0028(10)	–0.0011(11)
C(1)	0.0351(18)	0.0201(14)	0.0256(15)	0.0003(12)	–0.0012(13)	–0.0013(13)
C(2)	0.0284(17)	0.0210(15)	0.0313(16)	–0.0034(13)	–0.0033(13)	–0.0016(13)
C(3)	0.0234(15)	0.0170(14)	0.0278(15)	–0.0014(12)	0.0018(12)	0.0010(12)
C(4)	0.0265(16)	0.0183(14)	0.0190(13)	–0.0016(10)	–0.0006(12)	–0.0012(12)
C(5)	0.0275(16)	0.0132(13)	0.0165(13)	0.0002(10)	0.0009(11)	–0.0030(11)
C(6)	0.0273(16)	0.0169(14)	0.0167(13)	0.0015(10)	0.0028(11)	–0.0034(12)
C(7)	0.0281(16)	0.0180(14)	0.0190(14)	0.0009(11)	0.0038(12)	–0.0018(12)
C(8)	0.0268(15)	0.0171(14)	0.0245(14)	–0.0026(11)	0.0039(13)	–0.0015(13)
C(9)	0.0301(16)	0.0204(14)	0.0191(14)	0.0003(11)	0.0041(12)	–0.0007(12)
C(10)	0.040(2)	0.0364(19)	0.0300(17)	0.0070(15)	–0.0043(15)	0.0040(16)
C(11)	0.042(2)	0.0256(17)	0.057(2)	–0.0041(17)	–0.0026(18)	0.0088(16)
C(12)	0.0300(17)	0.0281(17)	0.0406(18)	–0.0006(14)	–0.0051(15)	0.0045(14)
C(13)	0.0278(16)	0.0252(16)	0.0292(16)	0.0000(13)	0.0076(13)	0.0009(13)
C(14)	0.0285(16)	0.0240(15)	0.0195(14)	0.0050(12)	0.0008(12)	–0.0015(13)
C(15)	0.0407(19)	0.0277(17)	0.0371(17)	–0.0021(13)	0.0014(17)	0.0116(16)
C(16)	0.0273(16)	0.0287(16)	0.0274(14)	–0.0023(12)	0.0043(14)	0.0013(14)
C(17)	0.0402(19)	0.0289(16)	0.0209(14)	–0.0013(12)	0.0070(15)	0.0050(16)
C(18)	0.0252(14)	0.0191(14)	0.0169(13)	0.0016(11)	0.0041(11)	0.0017(12)
C(19)	0.0305(16)	0.0206(14)	0.0194(13)	–0.0012(10)	0.0034(13)	–0.0021(14)
C(20)	0.0378(18)	0.0225(15)	0.0244(14)	0.0046(12)	0.0084(14)	–0.0013(14)
C(21)	0.039(2)	0.0329(17)	0.0161(13)	0.0026(12)	0.0042(13)	0.0074(14)
C(22)	0.0397(19)	0.0302(17)	0.0226(15)	–0.0067(13)	0.0016(13)	0.0012(15)
C(23)	0.0358(19)	0.0215(15)	0.0231(14)	–0.0013(11)	0.0017(13)	–0.0049(13)
C(24)	0.037(2)	0.0352(18)	0.0251(16)	0.0104(14)	–0.0013(14)	0.0013(15)
C(25)	0.040(2)	0.0375(19)	0.0293(17)	–0.0087(14)	–0.0014(15)	0.0005(16)

Table 11. Hydrogen coordinates and isotropic displacement parameters (Å^2) for **2b**.

	x	y	z	U
H(10A)	0.2945	0.4046	0.2627	0.053
H(10B)	0.3095	0.5411	0.2828	0.053
H(10C)	0.1297	0.4758	0.2809	0.053
H(11A)	–0.1029	0.6717	0.3824	0.062
H(11B)	0.0818	0.6682	0.3580	0.062
H(11C)	0.0582	0.6558	0.4190	0.062
H(12A)	–0.0775	0.4675	0.4073	0.039
H(12B)	–0.0566	0.4799	0.3463	0.039
H(13A)	0.0400	0.4048	0.4764	0.041
H(13B)	0.2191	0.4337	0.5021	0.041
H(13C)	0.1639	0.2965	0.4919	0.041
H(14A)	0.9638	0.1115	0.4923	0.036
H(14B)	0.7678	0.0834	0.5002	0.036
H(14C)	0.8395	0.2162	0.5114	0.036
H(15A)	1.1916	–0.0757	0.3802	0.053
H(15B)	1.0286	–0.0484	0.3457	0.053
H(15C)	1.0082	–0.0809	0.4054	0.053
H(16A)	1.1435	0.1027	0.4283	0.033
H(16B)	1.1671	0.1350	0.3687	0.033
H(17A)	0.8335	0.2010	0.2697	0.045
H(17B)	0.9872	0.1235	0.2922	0.045
H(17C)	0.9988	0.2680	0.2906	0.045
H(19)	0.3979	0.0892	0.4847	0.028
H(20)	0.3604	0.0488	0.5728	0.034
H(22)	0.5501	0.3790	0.6072	0.037
H(23)	0.5896	0.4177	0.5192	0.032
H(24A)	0.6476	0.4924	0.3088	0.049
H(24B)	0.5793	0.4320	0.2567	0.049
H(24C)	0.7617	0.3969	0.2784	0.049
H(25A)	0.4520	0.1320	0.3111	0.054
H(25B)	0.5764	0.1653	0.2648	0.054
H(25C)	0.3925	0.2243	0.2672	0.054

Table 12. Torsion angles [°] for **2b**.

N(2)–B–N(1)–C(1)	176.7(3)	N(2)–B–N(1)–C(4)	−11.4(4)
C(24)–B–N(1)–C(1)	58.0(4)	C(24)–B–N(1)–C(4)	−130.1(3)
C(25)–B–N(1)–C(1)	−69.0(4)	C(25)–B–N(1)–C(4)	102.8(3)
N(1)–B–N(2)–C(6)	9.2(4)	N(1)–B–N(2)–C(9)	−177.6(3)
C(24)–B–N(2)–C(6)	127.6(3)	C(24)–B–N(2)–C(9)	−59.2(4)
C(25)–B–N(2)–C(6)	−105.5(3)	C(25)–B–N(2)–C(9)	67.6(4)
B–N(1)–C(1)–C(2)	172.0(3)	B–N(1)–C(1)–C(10)	−9.6(5)
C(4)–N(1)–C(1)–C(2)	−1.1(3)	C(4)–N(1)–C(1)–C(10)	177.3(3)
N(1)–C(1)–C(2)–C(3)	0.5(4)	N(1)–C(1)–C(2)–C(12)	179.2(3)
C(10)–C(1)–C(2)–C(3)	−177.8(3)	C(10)–C(1)–C(2)–C(12)	0.9(5)
C(1)–C(2)–C(3)–C(4)	0.2(3)	C(1)–C(2)–C(3)–C(13)	−178.7(3)
C(12)–C(2)–C(3)–C(4)	−178.4(3)	C(12)–C(2)–C(3)–C(13)	2.6(5)
B–N(1)–C(4)–C(3)	−172.0(3)	B–N(1)–C(4)–C(5)	8.5(4)
C(1)–N(1)–C(4)–C(3)	1.2(3)	C(1)–N(1)–C(4)–C(5)	−178.3(3)
C(2)–C(3)–C(4)–N(1)	−0.9(3)	C(2)–C(3)–C(4)–C(5)	178.5(3)
C(13)–C(3)–C(4)–N(1)	178.0(3)	C(13)–C(3)–C(4)–C(5)	−2.6(5)
N(1)–C(4)–C(5)–C(6)	−1.3(4)	N(1)–C(4)–C(5)–C(18)	−179.3(3)
C(3)–C(4)–C(5)–C(6)	179.4(3)	C(3)–C(4)–C(5)–C(18)	1.3(5)
C(4)–C(5)–C(6)–N(2)	−0.8(4)	C(4)–C(5)–C(6)–C(7)	−180.0(3)
C(18)–C(5)–C(6)–N(2)	177.2(3)	C(18)–C(5)–C(6)–C(7)	−1.9(5)
B–N(2)–C(6)–C(5)	−4.2(4)	B–N(2)–C(6)–C(7)	175.1(2)
C(9)–N(2)–C(6)–C(5)	−178.5(3)	C(9)–N(2)–C(6)–C(7)	0.9(3)
N(2)–C(6)–C(7)–C(8)	−0.6(3)	N(2)–C(6)–C(7)–C(14)	178.9(3)
C(5)–C(6)–C(7)–C(8)	178.6(3)	C(5)–C(6)–C(7)–C(14)	−1.8(5)
C(6)–C(7)–C(8)–C(9)	0.2(3)	C(6)–C(7)–C(8)–C(16)	−178.5(3)
C(14)–C(7)–C(8)–C(9)	−179.4(3)	C(14)–C(7)–C(8)–C(16)	2.0(5)
B–N(2)–C(9)–C(8)	−174.9(3)	B–N(2)–C(9)–C(17)	4.7(5)
C(6)–N(2)–C(9)–C(8)	−0.8(3)	C(6)–N(2)–C(9)–C(17)	178.8(3)
C(7)–C(8)–C(9)–N(2)	0.4(3)	C(7)–C(8)–C(9)–C(17)	−179.2(3)
C(16)–C(8)–C(9)–N(2)	179.1(3)	C(16)–C(8)–C(9)–C(17)	−0.5(5)
C(1)–C(2)–C(12)–C(11)	−84.0(4)	C(3)–C(2)–C(12)–C(11)	94.4(4)
C(7)–C(8)–C(16)–C(15)	94.4(4)	C(9)–C(8)–C(16)–C(15)	−84.0(4)
C(4)–C(5)–C(18)–C(19)	92.4(3)	C(4)–C(5)–C(18)–C(23)	−88.0(4)
C(6)–C(5)–C(18)–C(19)	−85.7(4)	C(6)–C(5)–C(18)–C(23)	93.8(4)
C(5)–C(18)–C(19)–C(20)	179.1(3)	C(23)–C(18)–C(19)–C(20)	−0.5(5)
C(18)–C(19)–C(20)–C(21)	0.5(5)	C(19)–C(20)–C(21)–Br	−179.5(3)
C(19)–C(20)–C(21)–C(22)	−0.1(5)	Br–C(21)–C(22)–C(23)	179.2(3)
C(20)–C(21)–C(22)–C(23)	−0.2(5)	C(5)–C(18)–C(23)–C(22)	−179.4(3)
C(19)–C(18)–C(23)–C(22)	0.1(5)	C(21)–C(22)–C(23)–C(18)	0.2(5)

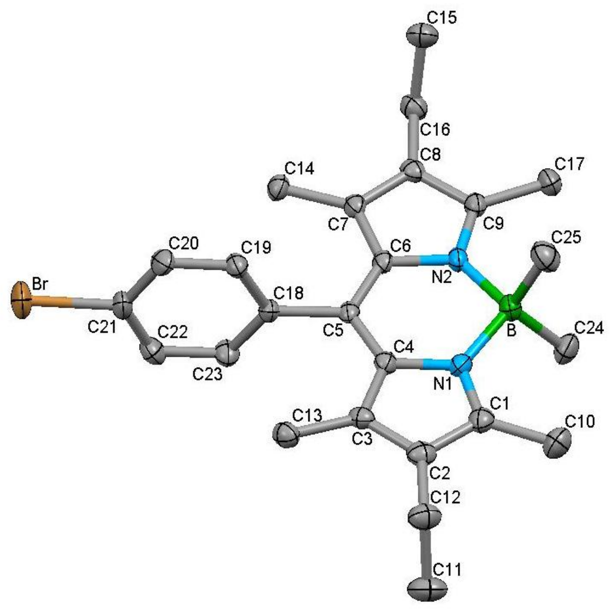

Figure S2. View of the molecular structure of **2b** with 50% probability displacement ellipsoids. Hydrogen atoms have been omitted for clarity.

3.4 Crystallographic data for **3a**

Table 13. Crystal data and structure refinement for **3a**.

Identification code	3a	
Chemical formula (moiety)	$C_{39}H_{46}BN_2O_3P$	
Chemical formula (total)	$C_{39}H_{46}BN_2O_3P$	
Formula weight	632.56	
Temperature	120(2) K	
Radiation, wavelength	synchrotron, 0.6889 Å	
Crystal system, space group	rhombohedral, $R\bar{3}$	
Unit cell parameters	a = 48.953(8) Å	α = 90°
	b = 48.953(8) Å	β = 90°
	c = 7.8657(12) Å	γ = 120°
Cell volume	16324(5) $Å^3$	
Z	18	
Calculated density	1.158 g/cm^3	
Absorption coefficient μ	0.109 mm^{-1}	
F(000)	6084	
Crystal colour and size	red, 0.10 × 0.00 × 0.00 mm^3	
Reflections for cell refinement	9848 (θ range 2.2 to 27.2°)	
Data collection method	Crystal Logic diffractometer and Rigaku Saturn 724+ CCD thick-slice ω scans	
θ range for data collection	3.5 to 24.2°	
Index ranges	h –58 to 58, k –58 to 58, l –7 to 9	
Completeness to θ = 24.2°	98.7 %	
Reflections collected	42859	
Independent reflections	6330 (R_{int} = 0.0584)	
Reflections with F^2>2σ	5133	
Absorption correction	semi-empirical from equivalents	
Min. and max. transmission	0.989 and 1.000	
Structure solution	direct methods	
Refinement method	Full-matrix least-squares on F^2	
Weighting parameters a, b	0.0974, 62.8825	
Data / restraints / parameters	6330 / 322 / 495	
Final R indices [F^2>2σ]	R1 = 0.0854, wR2 = 0.2190	
R indices (all data)	R1 = 0.0983, wR2 = 0.2268	
Goodness-of-fit on F^2	1.096	
Extinction coefficient	0.0019(4)	
Largest and mean shift/su	0.005 and 0.000	
Largest diff. peak and hole	0.76 and –0.64 e $Å^{-3}$	

Table 14. Atomic coordinates and equivalent isotropic displacement parameters (Å^2) for **3a**. U_{eq} is defined as one third of the trace of the orthogonalized U^{ij} tensor.

	x	y	z	U_{eq}
N(1)	0.54160(7)	0.14061(8)	0.1157(4)	0.0349(8)
N(2)	0.53204(7)	0.11503(7)	0.4038(4)	0.0351(8)
B(1)	0.55788(10)	0.13084(11)	0.2597(6)	0.0352(10)
C(1)	0.55498(9)	0.15688(10)	–0.0279(5)	0.0392(9)
C(2)	0.53205(10)	0.15892(10)	–0.1283(5)	0.0401(9)
C(3)	0.50387(9)	0.14364(9)	–0.0422(5)	0.0357(9)
C(4)	0.50959(8)	0.13187(9)	0.1110(5)	0.0316(8)
C(5)	0.48957(9)	0.11474(8)	0.2443(5)	0.0315(8)
C(6)	0.50021(8)	0.10672(9)	0.3889(5)	0.0327(8)
C(7)	0.48385(9)	0.09008(9)	0.5395(5)	0.0358(9)
C(8)	0.50592(10)	0.08844(9)	0.6409(5)	0.0386(9)
C(9)	0.53510(9)	0.10335(9)	0.5533(5)	0.0395(9)
C(10)	0.58881(11)	0.17102(12)	–0.0715(6)	0.0535(12)
C(11)	0.55026(16)	0.21035(13)	–0.2795(7)	0.0712(17)
C(12)	0.53852(12)	0.17548(11)	–0.2972(6)	0.0501(11)
C(13)	0.47388(10)	0.14184(11)	–0.1021(6)	0.0459(10)
C(14)	0.44989(10)	0.07709(10)	0.5844(6)	0.0435(10)
C(15)	0.48571(13)	0.03820(10)	0.8127(6)	0.0559(13)
C(16)	0.50045(11)	0.07389(10)	0.8152(5)	0.0455(10)
C(17)	0.56491(11)	0.10508(12)	0.6116(6)	0.0547(12)
C(18)	0.45529(9)	0.10389(9)	0.2281(5)	0.0341(9)
C(19)	0.43520(10)	0.07498(10)	0.1546(6)	0.0449(10)
C(20)	0.40347(10)	0.06461(12)	0.1378(7)	0.0552(12)
C(22)	0.41124(10)	0.11203(11)	0.2690(6)	0.0471(11)
C(23)	0.44297(9)	0.12219(10)	0.2870(5)	0.0389(9)
C(24)	0.58804(9)	0.16259(9)	0.3324(5)	0.0367(9)
C(25)	0.61611(10)	0.16441(10)	0.3878(6)	0.0438(10)
C(26)	0.64103(10)	0.19176(11)	0.4527(6)	0.0502(11)
C(27)	0.63906(11)	0.21879(11)	0.4638(6)	0.0526(12)
C(28)	0.61213(11)	0.21832(11)	0.4107(6)	0.0514(11)
C(29)	0.58700(9)	0.19055(10)	0.3461(6)	0.0419(10)
C(30)	0.56433(9)	0.10331(9)	0.1833(5)	0.0356(9)
C(31)	0.59195(10)	0.10927(10)	0.0980(5)	0.0412(10)
C(32)	0.59539(11)	0.08522(11)	0.0268(6)	0.0475(11)
C(33)	0.57165(11)	0.05442(11)	0.0390(6)	0.0489(11)
C(34)	0.54429(11)	0.04761(10)	0.1232(6)	0.0480(11)
C(35)	0.54077(9)	0.07170(10)	0.1924(6)	0.0414(10)
C(21)	0.39128(10)	0.08332(12)	0.1933(6)	0.0537(12)
P(1)	0.35103(9)	0.07169(10)	0.1419(5)	0.0607(13)
O(1)	0.34585(19)	0.09586(18)	0.0341(9)	0.070(2)
C(36)	0.3550(4)	0.1275(3)	0.062(3)	0.094(4)
C(37)	0.3310(5)	0.1324(5)	0.040(3)	0.091(5)
O(2)	0.33430(16)	0.0643(2)	0.3214(9)	0.0658(19)
C(38)	0.3374(3)	0.0624(4)	0.4850(14)	0.075(3)
C(39)	0.3526(4)	0.0601(4)	0.6041(17)	0.095(4)
O(3)	0.3343(2)	0.03545(17)	0.0745(14)	0.080(3)
P(1A)	0.34828(8)	0.06475(11)	0.2146(6)	0.0742(13)
O(1A)	0.34448(14)	0.09722(17)	0.2080(8)	0.0670(17)
C(36A)	0.3515(4)	0.1222(3)	0.108(2)	0.097(3)
C(37A)	0.3288(4)	0.1217(4)	0.011(2)	0.076(4)
O(2A)	0.33689(17)	0.0515(2)	0.4029(11)	0.0741(19)
C(38A)	0.3475(3)	0.0383(3)	0.5212(15)	0.091(3)
C(39A)	0.3603(4)	0.0519(3)	0.6664(18)	0.095(4)
O(3A)	0.3350(2)	0.0512(3)	0.0348(13)	0.127(4)

Table 15. Bond lengths [Å] and angles [°] for **3a**.

N(1)–B(1)	1.590(5)	N(1)–C(1)	1.348(5)
N(1)–C(4)	1.403(5)	N(2)–B(1)	1.583(5)
N(2)–C(6)	1.404(5)	N(2)–C(9)	1.348(5)
B(1)–C(24)	1.621(6)	B(1)–C(30)	1.644(6)
C(1)–C(2)	1.416(6)	C(1)–C(10)	1.481(6)
C(2)–C(3)	1.374(6)	C(2)–C(12)	1.506(6)
C(3)–C(4)	1.423(5)	C(3)–C(13)	1.502(5)
C(4)–C(5)	1.393(5)	C(5)–C(6)	1.388(5)
C(5)–C(18)	1.491(5)	C(6)–C(7)	1.434(5)
C(7)–C(8)	1.377(6)	C(7)–C(14)	1.495(5)
C(8)–C(9)	1.416(6)	C(8)–C(16)	1.506(6)
C(9)–C(17)	1.491(6)	C(10)–H(10A)	0.980
C(10)–H(10B)	0.980	C(10)–H(10C)	0.980
C(11)–H(11A)	0.980	C(11)–H(11B)	0.980
C(11)–H(11C)	0.980	C(11)–C(12)	1.511(7)
C(12)–H(12A)	0.990	C(12)–H(12B)	0.990
C(13)–H(13A)	0.980	C(13)–H(13B)	0.980
C(13)–H(13C)	0.980	C(14)–H(14A)	0.980
C(14)–H(14B)	0.980	C(14)–H(14C)	0.980
C(15)–H(15A)	0.980	C(15)–H(15B)	0.980
C(15)–H(15C)	0.980	C(15)–C(16)	1.521(6)
C(16)–H(16A)	0.990	C(16)–H(16B)	0.990
C(17)–H(17A)	0.980	C(17)–H(17B)	0.980
C(17)–H(17C)	0.980	C(18)–C(19)	1.383(6)
C(18)–C(23)	1.386(5)	C(19)–H(19)	0.950
C(19)–C(20)	1.378(6)	C(20)–H(20)	0.950
C(20)–C(21)	1.390(7)	C(22)–H(22)	0.950
C(22)–C(23)	1.381(6)	C(22)–C(21)	1.383(7)
C(23)–H(23)	0.950	C(24)–C(25)	1.401(6)
C(24)–C(29)	1.399(6)	C(25)–H(25)	0.950
C(25)–C(26)	1.381(6)	C(26)–H(26)	0.950
C(26)–C(27)	1.377(7)	C(27)–H(27)	0.950
C(27)–C(28)	1.372(7)	C(28)–H(28)	0.950
C(28)–C(29)	1.396(6)	C(29)–H(29)	0.950
C(30)–C(31)	1.403(6)	C(30)–C(35)	1.395(6)
C(31)–H(31)	0.950	C(31)–C(32)	1.388(6)
C(32)–H(32)	0.950	C(32)–C(33)	1.371(7)
C(33)–H(33)	0.950	C(33)–C(34)	1.377(7)
C(34)–H(34)	0.950	C(34)–C(35)	1.386(6)
C(35)–H(35)	0.950	C(21)–P(1)	1.802(6)
C(21)–P(1A)	1.837(5)	P(1)–O(1)	1.576(7)
P(1)–O(2)	1.581(7)	P(1)–O(3)	1.627(8)
O(1)–C(36)	1.397(12)	C(36)–H(36A)	0.990
C(36)–H(36B)	0.990	C(36)–C(37)	1.323(12)
C(37)–H(37A)	0.980	C(37)–H(37B)	0.980
C(37)–H(37C)	0.980	O(2)–C(38)	1.305(11)
C(38)–H(38A)	0.990	C(38)–H(38B)	0.990
C(38)–C(39)	1.235(12)	C(39)–H(39A)	0.980
C(39)–H(39B)	0.980	C(39)–H(39C)	0.980
P(1A)–O(1A)	1.691(7)	P(1A)–O(2A)	1.600(8)
P(1A)–O(3A)	1.560(8)	O(1A)–C(36A)	1.346(11)
C(36A)–H(36C)	0.990	C(36A)–H(36D)	0.990
C(36A)–C(37A)	1.336(12)	C(37A)–H(37D)	0.980
C(37A)–H(37E)	0.980	C(37A)–H(37F)	0.980
O(2A)–C(38A)	1.375(11)	C(38A)–H(38C)	0.990
C(38A)–H(38D)	0.990	C(38A)–C(39A)	1.314(13)
C(39A)–H(39D)	0.980	C(39A)–H(39E)	0.980
C(39A)–H(39F)	0.980		

B(1)–N(1)–C(1)	127.3(3)	B(1)–N(1)–C(4)	125.3(3)
C(1)–N(1)–C(4)	107.3(3)	B(1)–N(2)–C(6)	125.6(3)
B(1)–N(2)–C(9)	126.9(3)	C(6)–N(2)–C(9)	107.0(3)
N(1)–B(1)–N(2)	105.0(3)	N(1)–B(1)–C(24)	108.4(3)
N(1)–B(1)–C(30)	108.5(3)	N(2)–B(1)–C(24)	109.7(3)
N(2)–B(1)–C(30)	107.2(3)	C(24)–B(1)–C(30)	117.3(3)
N(1)–C(1)–C(2)	109.9(3)	N(1)–C(1)–C(10)	124.7(4)
C(2)–C(1)–C(10)	125.3(4)	C(1)–C(2)–C(3)	107.6(3)
C(1)–C(2)–C(12)	124.7(4)	C(3)–C(2)–C(12)	127.7(4)
C(2)–C(3)–C(4)	106.9(3)	C(2)–C(3)–C(13)	124.3(4)
C(4)–C(3)–C(13)	128.8(4)	N(1)–C(4)–C(3)	108.3(3)
N(1)–C(4)–C(5)	120.4(3)	C(3)–C(4)–C(5)	131.3(3)
C(4)–C(5)–C(6)	122.5(3)	C(4)–C(5)–C(18)	118.6(3)
C(6)–C(5)–C(18)	119.0(3)	N(2)–C(6)–C(5)	120.4(3)
N(2)–C(6)–C(7)	108.6(3)	C(5)–C(6)–C(7)	131.0(3)
C(6)–C(7)–C(8)	106.2(3)	C(6)–C(7)–C(14)	128.6(4)
C(8)–C(7)–C(14)	125.2(4)	C(7)–C(8)–C(9)	107.9(3)
C(7)–C(8)–C(16)	126.6(4)	C(9)–C(8)–C(16)	125.5(4)
N(2)–C(9)–C(8)	110.2(3)	N(2)–C(9)–C(17)	123.9(4)
C(8)–C(9)–C(17)	125.9(4)	C(1)–C(10)–H(10A)	109.5
C(1)–C(10)–H(10B)	109.5	C(1)–C(10)–H(10C)	109.5
H(10A)–C(10)–H(10B)	109.5	H(10A)–C(10)–H(10C)	109.5
H(10B)–C(10)–H(10C)	109.5	H(11A)–C(11)–H(11B)	109.5
H(11A)–C(11)–H(11C)	109.5	H(11A)–C(11)–C(12)	109.5
H(11B)–C(11)–H(11C)	109.5	H(11B)–C(11)–C(12)	109.5
H(11C)–C(11)–C(12)	109.5	C(2)–C(12)–C(11)	112.7(4)
C(2)–C(12)–H(12A)	109.1	C(2)–C(12)–H(12B)	109.1
C(11)–C(12)–H(12A)	109.1	C(11)–C(12)–H(12B)	109.1
H(12A)–C(12)–H(12B)	107.8	C(3)–C(13)–H(13A)	109.5
C(3)–C(13)–H(13B)	109.5	C(3)–C(13)–H(13C)	109.5
H(13A)–C(13)–H(13B)	109.5	H(13A)–C(13)–H(13C)	109.5
H(13B)–C(13)–H(13C)	109.5	C(7)–C(14)–H(14A)	109.5
C(7)–C(14)–H(14B)	109.5	C(7)–C(14)–H(14C)	109.5
H(14A)–C(14)–H(14B)	109.5	H(14A)–C(14)–H(14C)	109.5
H(14B)–C(14)–H(14C)	109.5	H(15A)–C(15)–H(15B)	109.5
H(15A)–C(15)–H(15C)	109.5	H(15A)–C(15)–C(16)	109.5
H(15B)–C(15)–H(15C)	109.5	H(15B)–C(15)–C(16)	109.5
H(15C)–C(15)–C(16)	109.5	C(8)–C(16)–C(15)	113.7(4)
C(8)–C(16)–H(16A)	108.8	C(8)–C(16)–H(16B)	108.8
C(15)–C(16)–H(16A)	108.8	C(15)–C(16)–H(16B)	108.8
H(16A)–C(16)–H(16B)	107.7	C(9)–C(17)–H(17A)	109.5
C(9)–C(17)–H(17B)	109.5	C(9)–C(17)–H(17C)	109.5
H(17A)–C(17)–H(17B)	109.5	H(17A)–C(17)–H(17C)	109.5
H(17B)–C(17)–H(17C)	109.5	C(5)–C(18)–C(19)	119.9(3)
C(5)–C(18)–C(23)	121.1(3)	C(19)–C(18)–C(23)	119.0(4)
C(18)–C(19)–H(19)	119.6	C(18)–C(19)–C(20)	120.7(4)
H(19)–C(19)–C(20)	119.6	C(19)–C(20)–H(20)	119.9
C(19)–C(20)–C(21)	120.2(4)	H(20)–C(20)–C(21)	119.9
H(22)–C(22)–C(23)	119.8	H(22)–C(22)–C(21)	119.8
C(23)–C(22)–C(21)	120.4(4)	C(18)–C(23)–C(22)	120.5(4)
C(18)–C(23)–H(23)	119.8	C(22)–C(23)–H(23)	119.8
B(1)–C(24)–C(25)	124.4(4)	B(1)–C(24)–C(29)	120.3(3)
C(25)–C(24)–C(29)	115.3(4)	C(24)–C(25)–H(25)	118.7
C(24)–C(25)–C(26)	122.7(4)	H(25)–C(25)–C(26)	118.7
C(25)–C(26)–H(26)	119.9	C(25)–C(26)–C(27)	120.2(4)
H(26)–C(26)–C(27)	119.9	C(26)–C(27)–H(27)	120.2
C(26)–C(27)–C(28)	119.6(4)	H(27)–C(27)–C(28)	120.2
C(27)–C(28)–H(28)	120.0	C(27)–C(28)–C(29)	119.9(4)
H(28)–C(28)–C(29)	120.0	C(24)–C(29)–C(28)	122.4(4)
C(24)–C(29)–H(29)	118.8	C(28)–C(29)–H(29)	118.8

B(1)–C(30)–C(31)	124.4(3)	B(1)–C(30)–C(35)	120.0(3)
C(31)–C(30)–C(35)	115.6(4)	C(30)–C(31)–H(31)	119.0
C(30)–C(31)–C(32)	122.0(4)	H(31)–C(31)–C(32)	119.0
C(31)–C(32)–H(32)	119.7	C(31)–C(32)–C(33)	120.6(4)
H(32)–C(32)–C(33)	119.7	C(32)–C(33)–H(33)	120.4
C(32)–C(33)–C(34)	119.1(4)	H(33)–C(33)–C(34)	120.4
C(33)–C(34)–H(34)	119.9	C(33)–C(34)–C(35)	120.2(4)
H(34)–C(34)–C(35)	119.9	C(30)–C(35)–C(34)	122.6(4)
C(30)–C(35)–H(35)	118.7	C(34)–C(35)–H(35)	118.7
C(20)–C(21)–C(22)	119.2(4)	C(20)–C(21)–P(1)	118.9(4)
C(20)–C(21)–P(1A)	117.8(4)	C(22)–C(21)–P(1)	121.5(4)
C(22)–C(21)–P(1A)	121.1(4)	C(21)–P(1)–O(1)	116.4(4)
C(21)–P(1)–O(2)	103.4(3)	C(21)–P(1)–O(3)	107.1(4)
O(1)–P(1)–O(2)	113.1(5)	O(1)–P(1)–O(3)	117.4(5)
O(2)–P(1)–O(3)	96.9(5)	P(1)–O(1)–C(36)	131.7(9)
O(1)–C(36)–H(36A)	109.4	O(1)–C(36)–H(36B)	109.4
O(1)–C(36)–C(37)	111.0(12)	H(36A)–C(36)–H(36B)	108.0
H(36A)–C(36)–C(37)	109.4	H(36B)–C(36)–C(37)	109.4
C(36)–C(37)–H(37A)	109.5	C(36)–C(37)–H(37B)	109.5
C(36)–C(37)–H(37C)	109.5	H(37A)–C(37)–H(37B)	109.5
H(37A)–C(37)–H(37C)	109.5	H(37B)–C(37)–H(37C)	109.5
P(1)–O(2)–C(38)	146.2(7)	O(2)–C(38)–H(38A)	99.6
O(2)–C(38)–H(38B)	99.6	O(2)–C(38)–C(39)	148.5(15)
H(38A)–C(38)–H(38B)	104.1	H(38A)–C(38)–C(39)	99.6
H(38B)–C(38)–C(39)	99.6	C(38)–C(39)–H(39A)	109.5
C(38)–C(39)–H(39B)	109.5	C(38)–C(39)–H(39C)	109.5
H(39A)–C(39)–H(39B)	109.5	H(39A)–C(39)–H(39C)	109.5
H(39B)–C(39)–H(39C)	109.5	C(21)–P(1A)–O(1A)	99.8(3)
C(21)–P(1A)–O(2A)	111.5(4)	C(21)–P(1A)–O(3A)	105.0(5)
O(1A)–P(1A)–O(2A)	103.2(4)	O(1A)–P(1A)–O(3A)	98.8(6)
O(2A)–P(1A)–O(3A)	132.9(6)	P(1A)–O(1A)–C(36A)	140.8(9)
O(1A)–C(36A)–H(36C)	107.6	O(1A)–C(36A)–H(36D)	107.6
O(1A)–C(36A)–C(37A)	118.9(12)	H(36C)–C(36A)–H(36D)	107.0
H(36C)–C(36A)–C(37A)	107.6	H(36D)–C(36A)–C(37A)	107.6
C(36A)–C(37A)–H(37D)	109.5	C(36A)–C(37A)–H(37E)	109.5
C(36A)–C(37A)–H(37F)	109.5	H(37D)–C(37A)–H(37E)	109.5
H(37D)–C(37A)–H(37F)	109.5	H(37E)–C(37A)–H(37F)	109.5
P(1A)–O(2A)–C(38A)	132.8(7)	O(2A)–C(38A)–H(38C)	106.6
O(2A)–C(38A)–H(38D)	106.6	O(2A)–C(38A)–C(39A)	123.1(12)
H(38C)–C(38A)–H(38D)	106.5	H(38C)–C(38A)–C(39A)	106.6
H(38D)–C(38A)–C(39A)	106.6	C(38A)–C(39A)–H(39D)	109.5
C(38A)–C(39A)–H(39E)	109.5	C(38A)–C(39A)–H(39F)	109.5
H(39D)–C(39A)–H(39E)	109.5	H(39D)–C(39A)–H(39F)	109.5
H(39E)–C(39A)–H(39F)	109.5		

Table 16. Anisotropic displacement parameters ($Å^2$) for **3a**. The anisotropic displacement factor exponent takes the form: $-2\pi^2[h^2a^{*2}U^{11} + ...+ 2hka^*b^*U^{12}]$

	U^{11}	U^{22}	U^{33}	U^{23}	U^{13}	U^{12}
N(1)	0.0311(16)	0.0399(17)	0.0364(19)	0.0064(14)	0.0043(13)	0.0196(14)
N(2)	0.0309(16)	0.0374(17)	0.0341(18)	0.0032(14)	–0.0022(13)	0.0150(14)
B(1)	0.029(2)	0.041(2)	0.038(3)	0.0054(19)	0.0007(18)	0.0193(19)
C(1)	0.039(2)	0.045(2)	0.037(2)	0.0089(18)	0.0103(17)	0.0226(18)
C(2)	0.045(2)	0.045(2)	0.036(2)	0.0061(17)	0.0056(17)	0.0266(19)
C(3)	0.038(2)	0.039(2)	0.033(2)	0.0013(16)	–0.0001(16)	0.0221(17)
C(4)	0.0297(18)	0.0353(19)	0.033(2)	0.0012(15)	0.0001(15)	0.0187(16)
C(5)	0.0326(19)	0.0304(18)	0.033(2)	–0.0020(15)	0.0000(15)	0.0169(15)
C(6)	0.0296(18)	0.0324(19)	0.035(2)	0.0001(15)	0.0011(15)	0.0145(15)
C(7)	0.038(2)	0.0305(19)	0.034(2)	0.0020(15)	0.0047(16)	0.0138(16)
C(8)	0.047(2)	0.035(2)	0.028(2)	0.0010(16)	–0.0005(17)	0.0161(18)
C(9)	0.039(2)	0.040(2)	0.036(2)	0.0009(17)	–0.0063(17)	0.0171(18)
C(10)	0.045(2)	0.072(3)	0.049(3)	0.020(2)	0.016(2)	0.033(2)
C(11)	0.112(5)	0.075(4)	0.055(3)	0.029(3)	0.030(3)	0.068(4)
C(12)	0.060(3)	0.062(3)	0.038(2)	0.015(2)	0.010(2)	0.038(2)
C(13)	0.045(2)	0.059(3)	0.041(2)	0.007(2)	–0.0039(19)	0.032(2)
C(14)	0.041(2)	0.044(2)	0.040(2)	0.0079(18)	0.0095(18)	0.0171(19)
C(15)	0.071(3)	0.039(2)	0.043(3)	0.0062(19)	–0.010(2)	0.017(2)
C(16)	0.053(3)	0.038(2)	0.034(2)	0.0037(17)	–0.0024(19)	0.0147(19)
C(17)	0.049(3)	0.069(3)	0.045(3)	0.011(2)	–0.010(2)	0.029(2)
C(18)	0.0321(19)	0.036(2)	0.034(2)	0.0045(16)	0.0026(15)	0.0174(16)
C(19)	0.036(2)	0.043(2)	0.058(3)	–0.010(2)	–0.0042(19)	0.0209(18)
C(20)	0.036(2)	0.054(3)	0.071(3)	–0.015(2)	–0.012(2)	0.019(2)
C(22)	0.043(2)	0.060(3)	0.050(3)	–0.002(2)	0.0011(19)	0.035(2)
C(23)	0.037(2)	0.038(2)	0.044(2)	–0.0034(17)	–0.0016(17)	0.0207(17)
C(24)	0.0319(19)	0.041(2)	0.038(2)	0.0043(17)	0.0052(16)	0.0184(17)
C(25)	0.036(2)	0.046(2)	0.052(3)	–0.0018(19)	–0.0011(18)	0.0216(19)
C(26)	0.034(2)	0.058(3)	0.054(3)	–0.005(2)	–0.0044(19)	0.020(2)
C(27)	0.042(2)	0.046(2)	0.056(3)	–0.005(2)	0.000(2)	0.011(2)
C(28)	0.051(3)	0.040(2)	0.059(3)	0.000(2)	0.010(2)	0.019(2)
C(29)	0.035(2)	0.044(2)	0.047(3)	0.0046(19)	0.0067(18)	0.0210(18)
C(30)	0.0327(19)	0.039(2)	0.036(2)	0.0022(16)	–0.0054(16)	0.0181(17)
C(31)	0.037(2)	0.042(2)	0.045(2)	0.0023(18)	0.0009(18)	0.0201(18)
C(32)	0.049(2)	0.056(3)	0.044(3)	–0.004(2)	0.002(2)	0.030(2)
C(33)	0.057(3)	0.047(2)	0.048(3)	–0.010(2)	–0.008(2)	0.030(2)
C(34)	0.047(2)	0.039(2)	0.056(3)	–0.001(2)	–0.009(2)	0.020(2)
C(35)	0.035(2)	0.043(2)	0.047(3)	0.0050(18)	–0.0010(18)	0.0193(18)
C(21)	0.033(2)	0.067(3)	0.062(3)	–0.009(2)	–0.0053(19)	0.026(2)
P(1)	0.0281(14)	0.092(2)	0.053(2)	0.0422(19)	0.0028(14)	0.0228(13)
O(1)	0.075(4)	0.084(4)	0.048(4)	0.005(3)	0.007(3)	0.037(3)
C(36)	0.096(6)	0.103(5)	0.082(7)	0.022(5)	0.009(6)	0.050(5)
C(37)	0.108(8)	0.093(8)	0.094(9)	0.030(7)	0.012(7)	0.066(7)
O(2)	0.034(3)	0.115(5)	0.062(4)	0.007(3)	0.007(3)	0.048(3)
C(38)	0.058(5)	0.092(6)	0.083(5)	0.021(5)	0.014(4)	0.043(4)
C(39)	0.091(8)	0.080(7)	0.069(7)	0.010(6)	–0.010(6)	0.010(6)
O(3)	0.054(4)	0.044(4)	0.154(8)	–0.023(4)	–0.050(5)	0.035(4)
P(1A)	0.0360(16)	0.109(3)	0.083(3)	–0.033(2)	–0.0175(19)	0.0398(19)
O(1A)	0.047(3)	0.139(4)	0.042(3)	0.017(3)	0.007(2)	0.066(3)
C(36A)	0.088(5)	0.123(5)	0.079(6)	0.004(5)	0.004(5)	0.051(5)
C(37A)	0.068(6)	0.088(8)	0.090(7)	0.004(6)	0.002(5)	0.053(6)
O(2A)	0.045(3)	0.090(4)	0.108(5)	–0.014(4)	0.022(3)	0.049(3)
C(38A)	0.073(5)	0.094(6)	0.099(6)	0.008(5)	0.027(5)	0.038(5)
C(39A)	0.098(7)	0.084(7)	0.125(8)	0.014(6)	0.005(6)	0.061(6)

O(3A)	0.053(5)	0.208(10)	0.125(7)	–0.087(7)	–0.049(5)	0.069(7)

Table 17. Hydrogen coordinates and isotropic displacement parameters (Å^2) for **3a**.

	x	y	z	U
H(10A)	0.6017	0.1845	0.0222	0.080
H(10B)	0.5933	0.1837	–0.1751	0.080
H(10C)	0.5938	0.1542	–0.0905	0.080
H(11A)	0.5350	0.2134	–0.2133	0.107
H(11B)	0.5527	0.2197	–0.3925	0.107
H(11C)	0.5707	0.2205	–0.2211	0.107
H(12A)	0.5189	0.1657	–0.3656	0.060
H(12B)	0.5545	0.1726	–0.3593	0.060
H(13A)	0.4695	0.1559	–0.0329	0.069
H(13B)	0.4564	0.1201	–0.0911	0.069
H(13C)	0.4761	0.1484	–0.2215	0.069
H(14A)	0.4461	0.0683	0.6996	0.065
H(14B)	0.4366	0.0605	0.5035	0.065
H(14C)	0.4447	0.0940	0.5794	0.065
H(15A)	0.4655	0.0288	0.7526	0.084
H(15B)	0.4823	0.0302	0.9296	0.084
H(15C)	0.4999	0.0326	0.7543	0.084
H(16A)	0.4865	0.0794	0.8798	0.055
H(16B)	0.5209	0.0832	0.8759	0.055
H(17A)	0.5725	0.0962	0.5238	0.082
H(17B)	0.5610	0.0930	0.7170	0.082
H(17C)	0.5809	0.1271	0.6321	0.082
H(19)	0.4434	0.0621	0.1153	0.054
H(20)	0.3899	0.0446	0.0880	0.066
H(22)	0.4031	0.1249	0.3088	0.056
H(23)	0.4565	0.1419	0.3401	0.047
H(25)	0.6181	0.1461	0.3803	0.053
H(26)	0.6596	0.1919	0.4899	0.060
H(27)	0.6562	0.2376	0.5079	0.063
H(28)	0.6106	0.2369	0.4180	0.062
H(29)	0.5685	0.1906	0.3101	0.050
H(31)	0.6088	0.1304	0.0887	0.049
H(32)	0.6144	0.0901	–0.0308	0.057
H(33)	0.5740	0.0380	–0.0100	0.059
H(34)	0.5278	0.0263	0.1338	0.058
H(35)	0.5216	0.0665	0.2483	0.050
H(36A)	0.3631	0.1335	0.1793	0.113
H(36B)	0.3722	0.1409	–0.0176	0.113
H(37A)	0.3378	0.1545	0.0624	0.137
H(37B)	0.3139	0.1189	0.1179	0.137
H(37C)	0.3234	0.1272	–0.0777	0.137
H(38A)	0.3364	0.0811	0.5229	0.090
H(38B)	0.3163	0.0444	0.5119	0.090
H(39A)	0.3438	0.0621	0.7121	0.142
H(39B)	0.3747	0.0769	0.5954	0.142
H(39C)	0.3514	0.0395	0.5994	0.142
H(36C)	0.3598	0.1410	0.1823	0.117
H(36D)	0.3690	0.1253	0.0320	0.117
H(37D)	0.3377	0.1408	–0.0582	0.114
H(37E)	0.3121	0.1207	0.0842	0.114
H(37F)	0.3200	0.1031	–0.0629	0.114
H(38C)	0.3293	0.0173	0.5495	0.109
H(38D)	0.3631	0.0343	0.4624	0.109
H(39D)	0.3664	0.0386	0.7301	0.143

H(39E)	0.3451	0.0549	0.7329	0.143
H(39F)	0.3791	0.0725	0.6445	0.143

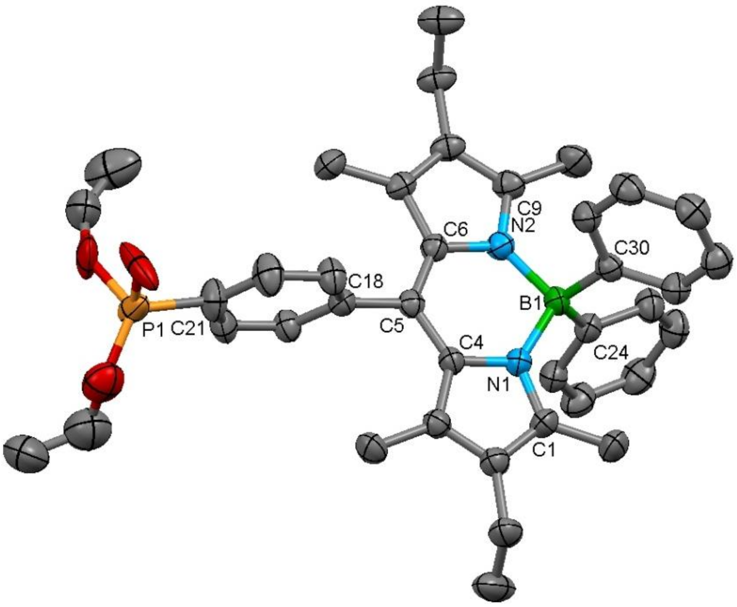

Figure S3. View of the molecular structure of **3a** with 50% probability displacement ellipsoids. Hydrogen atoms have been omitted for clarity.

3.5 Crystallographic data for **4b**

Table 18. Crystal data and structure refinement for **4b**.

Identification code	4b	
Chemical formula (moiety)	$C_{25}H_{34}BN_2P$	
Chemical formula (total)	$C_{25}H_{34}BN_2P$	
Formula weight	404.32	
Temperature	150(2) K	
Radiation, wavelength	MoKα, 0.71073 Å	
Crystal system, space group	monoclinic, $P2_1/n$	
Unit cell parameters	a = 7.9693(6) Å	α = 90°
	b = 11.0597(7) Å	β = 90.343(6)°
	c = 26.1232(16) Å	γ = 90°
Cell volume	2302.4(3) $Å^3$	
Z	4	
Calculated density	1.166 g/cm^3	
Absorption coefficient μ	0.133 mm^{-1}	
F(000)	872	
Crystal colour and size	red, 0.34 × 0.30 × 0.12 mm^3	
Reflections for cell refinement	6996 (θ range 3.0 to 28.6°)	
Data collection method	Oxford Diffraction Gemini A Ultra diffractometer<br>thick-slice ω scans	
θ range for data collection	3.1 to 28.7°	
Index ranges	h –10 to 10, k –14 to 14, l –31 to 32	
Completeness to θ = 26.0°	98.0 %	
Reflections collected	4902	
Independent reflections	4902 (R_{int} = 0.0000)	
Reflections with $F^2>2\sigma$	3829	
Absorption correction	semi-empirical from equivalents	
Min. and max. transmission	0.956 and 0.982	
Structure solution	direct methods	
Refinement method	Full-matrix least-squares on F^2	
Weighting parameters a, b	0.0678, 0.0000	
Data / restraints / parameters	4902 / 0 / 280	
Final R indices [$F^2>2\sigma$]	R1 = 0.0401, wR2 = 0.1031	
R indices (all data)	R1 = 0.0547, wR2 = 0.1078	
Goodness-of-fit on F^2	1.031	
Extinction coefficient	0.0020(10)	
Largest and mean shift/su	0.001 and 0.000	
Largest diff. peak and hole	0.21 and –0.30 e $Å^{-3}$	

Table 19. Atomic coordinates and equivalent isotropic displacement parameters (Å^2) for **4b**. U_{eq} is defined as one third of the trace of the orthogonalized U^{ij} tensor.

	x	y	z	U_{eq}
P(1)	0.06638(10)	0.82111(6)	0.16596(2)	0.05186(19)
N(1)	–0.19581(17)	0.74610(12)	–0.13925(5)	0.0220(3)
N(2)	0.08490(18)	0.64083(12)	–0.14301(5)	0.0232(3)
B(1)	–0.0635(3)	0.69089(18)	–0.17819(7)	0.0254(4)
C(1)	–0.3451(2)	0.79568(14)	–0.15096(6)	0.0238(4)
C(2)	–0.4221(2)	0.84220(14)	–0.10615(6)	0.0230(4)
C(3)	–0.3136(2)	0.82080(13)	–0.06585(6)	0.0208(3)
C(4)	–0.1705(2)	0.76069(14)	–0.08642(6)	0.0200(3)
C(5)	–0.0229(2)	0.72079(13)	–0.06330(6)	0.0196(3)
C(6)	0.1041(2)	0.66264(14)	–0.09050(6)	0.0211(4)
C(7)	0.2625(2)	0.61539(14)	–0.07437(7)	0.0246(4)
C(8)	0.3355(2)	0.56512(15)	–0.11704(7)	0.0284(4)
C(9)	0.2235(2)	0.58152(15)	–0.15866(7)	0.0285(4)
C(10)	–0.4212(3)	0.80122(17)	–0.20313(7)	0.0341(4)
C(11)	–0.5769(3)	1.03908(17)	–0.11912(8)	0.0397(5)
C(12)	–0.5885(2)	0.90497(16)	–0.10566(7)	0.0293(4)
C(13)	–0.3463(2)	0.85548(15)	–0.01132(6)	0.0248(4)
C(14)	0.3412(2)	0.61987(16)	–0.02207(7)	0.0308(4)
C(15)	0.5013(2)	0.50130(17)	–0.11975(8)	0.0364(5)
C(16)	0.4874(3)	0.36569(18)	–0.11188(9)	0.0478(6)
C(17)	0.2508(3)	0.53762(19)	–0.21203(7)	0.0407(5)
C(18)	0.0027(2)	0.74328(14)	–0.00743(6)	0.0200(3)
C(19)	–0.0419(2)	0.65773(15)	0.02900(6)	0.0265(4)
C(20)	–0.0199(2)	0.68134(16)	0.08067(7)	0.0312(4)
C(21)	0.0478(2)	0.79029(16)	0.09737(6)	0.0282(4)
C(22)	0.0929(2)	0.87554(15)	0.06063(7)	0.0275(4)
C(23)	0.0703(2)	0.85170(14)	0.00912(6)	0.0236(4)
C(24)	–0.1458(3)	0.58060(18)	–0.21018(7)	0.0377(5)
C(25)	0.0104(3)	0.80082(18)	–0.21245(8)	0.0395(5)

Table 20. Bond lengths [Å] and angles [°] for **4b**.

P(1)–H(1A)	1.42(4)	P(1)–H(1B)	1.23(4)
P(1)–C(21)	1.8289(17)	N(1)–B(1)	1.591(2)
N(1)–C(1)	1.344(2)	N(1)–C(4)	1.403(2)
N(2)–B(1)	1.593(2)	N(2)–C(6)	1.400(2)
N(2)–C(9)	1.350(2)	B(1)–C(24)	1.615(3)
B(1)–C(25)	1.622(3)	C(1)–C(2)	1.421(2)
C(1)–C(10)	1.490(2)	C(2)–C(3)	1.379(2)
C(2)–C(12)	1.497(3)	C(3)–C(4)	1.427(2)
C(3)–C(13)	1.500(2)	C(4)–C(5)	1.391(2)
C(5)–C(6)	1.397(2)	C(5)–C(18)	1.493(2)
C(6)–C(7)	1.427(2)	C(7)–C(8)	1.378(2)
C(7)–C(14)	1.501(2)	C(8)–C(9)	1.414(3)
C(8)–C(15)	1.500(3)	C(9)–C(17)	1.494(2)
C(10)–H(10A)	0.980	C(10)–H(10B)	0.980
C(10)–H(10C)	0.980	C(11)–H(11A)	0.980
C(11)–H(11B)	0.980	C(11)–H(11C)	0.980
C(11)–C(12)	1.527(3)	C(12)–H(12A)	0.990
C(12)–H(12B)	0.990	C(13)–H(13A)	0.980
C(13)–H(13B)	0.980	C(13)–H(13C)	0.980
C(14)–H(14A)	0.980	C(14)–H(14B)	0.980
C(14)–H(14C)	0.980	C(15)–H(15A)	0.990
C(15)–H(15B)	0.990	C(15)–C(16)	1.518(3)
C(16)–H(16A)	0.980	C(16)–H(16B)	0.980
C(16)–H(16C)	0.980	C(17)–H(17A)	0.980
C(17)–H(17B)	0.980	C(17)–H(17C)	0.980
C(18)–C(19)	1.390(2)	C(18)–C(23)	1.383(2)
C(19)–H(19)	0.950	C(19)–C(20)	1.385(2)
C(20)–H(20)	0.950	C(20)–C(21)	1.389(3)
C(21)–C(22)	1.394(3)	C(22)–H(22)	0.950
C(22)–C(23)	1.382(2)	C(23)–H(23)	0.950
C(24)–H(24A)	0.980	C(24)–H(24B)	0.980
C(24)–H(24C)	0.980	C(25)–H(25A)	0.980
C(25)–H(25B)	0.980	C(25)–H(25C)	0.980
H(1A)–P(1)–H(1B)	87(2)	H(1A)–P(1)–C(21)	96.6(13)
H(1B)–P(1)–C(21)	101.0(15)	B(1)–N(1)–C(1)	126.88(14)
B(1)–N(1)–C(4)	125.54(14)	C(1)–N(1)–C(4)	107.38(13)
B(1)–N(2)–C(6)	125.59(13)	B(1)–N(2)–C(9)	126.89(14)
C(6)–N(2)–C(9)	107.17(14)	N(1)–B(1)–N(2)	104.89(12)
N(1)–B(1)–C(24)	110.61(15)	N(1)–B(1)–C(25)	108.01(14)
N(2)–B(1)–C(24)	109.49(15)	N(2)–B(1)–C(25)	107.95(15)
C(24)–B(1)–C(25)	115.34(16)	N(1)–C(1)–C(2)	110.25(14)
N(1)–C(1)–C(10)	125.46(16)	C(2)–C(1)–C(10)	124.29(16)
C(1)–C(2)–C(3)	107.16(15)	C(1)–C(2)–C(12)	124.16(16)
C(3)–C(2)–C(12)	128.66(15)	C(2)–C(3)–C(4)	106.92(14)
C(2)–C(3)–C(13)	124.71(15)	C(4)–C(3)–C(13)	128.37(15)
N(1)–C(4)–C(3)	108.28(14)	N(1)–C(4)–C(5)	120.43(15)
C(3)–C(4)–C(5)	131.27(15)	C(4)–C(5)–C(6)	122.60(15)
C(4)–C(5)–C(18)	118.78(14)	C(6)–C(5)–C(18)	118.61(14)
N(2)–C(6)–C(5)	120.14(14)	N(2)–C(6)–C(7)	108.52(14)
C(5)–C(6)–C(7)	131.33(15)	C(6)–C(7)–C(8)	106.62(15)
C(6)–C(7)–C(14)	128.37(15)	C(8)–C(7)–C(14)	125.00(16)
C(7)–C(8)–C(9)	107.62(15)	C(7)–C(8)–C(15)	127.20(18)
C(9)–C(8)–C(15)	125.16(16)	N(2)–C(9)–C(8)	110.07(15)
N(2)–C(9)–C(17)	124.37(17)	C(8)–C(9)–C(17)	125.55(16)
C(1)–C(10)–H(10A)	109.5	C(1)–C(10)–H(10B)	109.5
C(1)–C(10)–H(10C)	109.5	H(10A)–C(10)–H(10B)	109.5
H(10A)–C(10)–H(10C)	109.5	H(10B)–C(10)–H(10C)	109.5

H(11A)–C(11)–H(11B)	109.5	H(11A)–C(11)–H(11C)	109.5
H(11A)–C(11)–C(12)	109.5	H(11B)–C(11)–H(11C)	109.5
H(11B)–C(11)–C(12)	109.5	H(11C)–C(11)–C(12)	109.5
C(2)–C(12)–C(11)	113.19(16)	C(2)–C(12)–H(12A)	108.9
C(2)–C(12)–H(12B)	108.9	C(11)–C(12)–H(12A)	108.9
C(11)–C(12)–H(12B)	108.9	H(12A)–C(12)–H(12B)	107.8
C(3)–C(13)–H(13A)	109.5	C(3)–C(13)–H(13B)	109.5
C(3)–C(13)–H(13C)	109.5	H(13A)–C(13)–H(13B)	109.5
H(13A)–C(13)–H(13C)	109.5	H(13B)–C(13)–H(13C)	109.5
C(7)–C(14)–H(14A)	109.5	C(7)–C(14)–H(14B)	109.5
C(7)–C(14)–H(14C)	109.5	H(14A)–C(14)–H(14B)	109.5
H(14A)–C(14)–H(14C)	109.5	H(14B)–C(14)–H(14C)	109.5
C(8)–C(15)–H(15A)	108.9	C(8)–C(15)–H(15B)	108.9
C(8)–C(15)–C(16)	113.17(17)	H(15A)–C(15)–H(15B)	107.8
H(15A)–C(15)–C(16)	108.9	H(15B)–C(15)–C(16)	108.9
C(15)–C(16)–H(16A)	109.5	C(15)–C(16)–H(16B)	109.5
C(15)–C(16)–H(16C)	109.5	H(16A)–C(16)–H(16B)	109.5
H(16A)–C(16)–H(16C)	109.5	H(16B)–C(16)–H(16C)	109.5
C(9)–C(17)–H(17A)	109.5	C(9)–C(17)–H(17B)	109.5
C(9)–C(17)–H(17C)	109.5	H(17A)–C(17)–H(17B)	109.5
H(17A)–C(17)–H(17C)	109.5	H(17B)–C(17)–H(17C)	109.5
C(5)–C(18)–C(19)	121.47(14)	C(5)–C(18)–C(23)	120.05(14)
C(19)–C(18)–C(23)	118.48(15)	C(18)–C(19)–H(19)	119.7
C(18)–C(19)–C(20)	120.51(16)	H(19)–C(19)–C(20)	119.7
C(19)–C(20)–H(20)	119.5	C(19)–C(20)–C(21)	121.09(16)
H(20)–C(20)–C(21)	119.5	P(1)–C(21)–C(20)	119.89(14)
P(1)–C(21)–C(22)	121.92(14)	C(20)–C(21)–C(22)	118.13(16)
C(21)–C(22)–H(22)	119.7	C(21)–C(22)–C(23)	120.58(16)
H(22)–C(22)–C(23)	119.7	C(18)–C(23)–C(22)	121.21(15)
C(18)–C(23)–H(23)	119.4	C(22)–C(23)–H(23)	119.4
B(1)–C(24)–H(24A)	109.5	B(1)–C(24)–H(24B)	109.5
B(1)–C(24)–H(24C)	109.5	H(24A)–C(24)–H(24B)	109.5
H(24A)–C(24)–H(24C)	109.5	H(24B)–C(24)–H(24C)	109.5
B(1)–C(25)–H(25A)	109.5	B(1)–C(25)–H(25B)	109.5
B(1)–C(25)–H(25C)	109.5	H(25A)–C(25)–H(25B)	109.5
H(25A)–C(25)–H(25C)	109.5	H(25B)–C(25)–H(25C)	109.5

Table 21. Anisotropic displacement parameters (Å^2) for **4b**. The anisotropic displacement factor exponent takes the form: $-2\pi^2[h^2a^{*2}U^{11} + ...+ 2hka^*b^*U^{12}]$

	U^{11}	U^{22}	U^{33}	U^{23}	U^{13}	U^{12}
P(1)	0.0736(4)	0.0631(4)	0.0187(3)	–0.0049(2)	–0.0064(3)	0.0095(3)
N(1)	0.0281(8)	0.0212(7)	0.0167(7)	–0.0004(5)	–0.0009(6)	–0.0010(6)
N(2)	0.0278(8)	0.0204(7)	0.0215(7)	–0.0020(5)	0.0056(6)	–0.0001(6)
B(1)	0.0298(11)	0.0282(10)	0.0183(10)	–0.0024(7)	0.0031(8)	0.0004(8)
C(1)	0.0289(9)	0.0209(8)	0.0214(9)	0.0025(7)	–0.0032(7)	–0.0021(7)
C(2)	0.0261(9)	0.0194(8)	0.0235(9)	0.0020(6)	–0.0007(7)	–0.0012(7)
C(3)	0.0231(8)	0.0165(8)	0.0227(9)	–0.0003(6)	0.0002(6)	–0.0033(6)
C(4)	0.0269(9)	0.0159(8)	0.0171(8)	–0.0010(6)	–0.0005(6)	–0.0030(7)
C(5)	0.0246(9)	0.0140(7)	0.0202(8)	0.0008(6)	0.0008(7)	–0.0044(6)
C(6)	0.0252(9)	0.0161(8)	0.0220(8)	0.0006(6)	0.0006(7)	–0.0028(6)
C(7)	0.0251(9)	0.0145(8)	0.0343(10)	0.0015(7)	0.0008(7)	–0.0022(7)
C(8)	0.0299(10)	0.0173(8)	0.0382(11)	0.0020(7)	0.0092(8)	–0.0009(7)
C(9)	0.0333(10)	0.0203(8)	0.0319(10)	–0.0013(7)	0.0102(8)	–0.0003(7)
C(10)	0.0394(11)	0.0369(10)	0.0259(10)	0.0000(8)	–0.0062(8)	0.0022(9)
C(11)	0.0426(11)	0.0303(10)	0.0462(12)	0.0057(9)	0.0027(10)	0.0098(9)
C(12)	0.0265(9)	0.0302(9)	0.0313(10)	0.0017(8)	–0.0028(7)	0.0031(8)
C(13)	0.0240(9)	0.0260(9)	0.0244(9)	–0.0040(7)	0.0021(7)	–0.0001(7)
C(14)	0.0277(10)	0.0256(9)	0.0389(11)	0.0035(8)	–0.0056(8)	0.0002(8)
C(15)	0.0300(10)	0.0329(10)	0.0463(12)	0.0045(9)	0.0104(9)	0.0067(8)
C(16)	0.0438(13)	0.0319(11)	0.0678(15)	0.0042(10)	0.0089(11)	0.0126(10)
C(17)	0.0467(12)	0.0398(11)	0.0359(12)	–0.0076(9)	0.0158(9)	0.0052(9)
C(18)	0.0187(8)	0.0200(8)	0.0212(9)	0.0007(6)	–0.0005(6)	0.0023(6)
C(19)	0.0324(10)	0.0211(8)	0.0260(9)	0.0013(7)	–0.0005(7)	–0.0028(7)
C(20)	0.0388(11)	0.0306(10)	0.0243(10)	0.0088(7)	0.0014(7)	–0.0002(8)
C(21)	0.0320(10)	0.0344(9)	0.0182(9)	–0.0008(7)	–0.0032(7)	0.0067(8)
C(22)	0.0325(10)	0.0237(9)	0.0263(9)	–0.0044(7)	–0.0047(7)	0.0002(8)
C(23)	0.0275(9)	0.0215(8)	0.0217(9)	0.0030(6)	–0.0012(7)	–0.0005(7)
C(24)	0.0376(11)	0.0425(11)	0.0331(11)	–0.0171(9)	0.0047(9)	–0.0023(9)
C(25)	0.0443(12)	0.0420(11)	0.0324(11)	0.0114(9)	0.0080(9)	0.0024(9)

Table 22. Hydrogen coordinates and isotropic displacement parameters (Å^2) for **4b**.

	x	y	z	U
H(1A)	0.155(5)	0.932(3)	0.1613(12)	0.117(11)
H(1B)	0.202(4)	0.773(3)	0.1759(13)	0.107(11)
H(10A)	–0.3320	0.8031	–0.2288	0.051
H(10B)	–0.4899	0.8744	–0.2062	0.051
H(10C)	–0.4918	0.7298	–0.2087	0.051
H(11A)	–0.6881	1.0762	–0.1162	0.060
H(11B)	–0.5366	1.0479	–0.1543	0.060
H(11C)	–0.4986	1.0791	–0.0955	0.060
H(12A)	–0.6383	0.8967	–0.0712	0.035
H(12B)	–0.6645	0.8647	–0.1304	0.035
H(13A)	–0.4612	0.8861	–0.0083	0.037
H(13B)	–0.2669	0.9185	–0.0008	0.037
H(13C)	–0.3322	0.7845	0.0107	0.037
H(14A)	0.4594	0.5961	–0.0243	0.046
H(14B)	0.2821	0.5642	0.0008	0.046
H(14C)	0.3335	0.7023	–0.0085	0.046
H(15A)	0.5774	0.5352	–0.0933	0.044
H(15B)	0.5521	0.5170	–0.1536	0.044
H(16A)	0.5999	0.3299	–0.1116	0.072
H(16B)	0.4212	0.3303	–0.1398	0.072
H(16C)	0.4323	0.3494	–0.0792	0.072
H(17A)	0.2120	0.5991	–0.2364	0.061
H(17B)	0.1878	0.4626	–0.2175	0.061
H(17C)	0.3707	0.5224	–0.2173	0.061
H(19)	–0.0878	0.5825	0.0184	0.032
H(20)	–0.0515	0.6221	0.1051	0.037
H(22)	0.1396	0.9507	0.0711	0.033
H(23)	0.1018	0.9109	–0.0154	0.028
H(24A)	–0.1513	0.5086	–0.1884	0.057
H(24B)	–0.0767	0.5633	–0.2402	0.057
H(24C)	–0.2592	0.6030	–0.2213	0.057
H(25A)	0.0616	0.8615	–0.1899	0.059
H(25B)	–0.0810	0.8380	–0.2321	0.059
H(25C)	0.0952	0.7693	–0.2360	0.059

Table 23. Torsion angles [°] for **4b**.

C(1)–N(1)–B(1)–N(2)	−177.72(14)	C(1)–N(1)–B(1)–C(24)	−59.8(2)
C(1)–N(1)–B(1)–C(25)	67.3(2)	C(4)–N(1)–B(1)–N(2)	7.9(2)
C(4)–N(1)–B(1)–C(24)	125.90(17)	C(4)–N(1)–B(1)–C(25)	−107.01(17)
C(6)–N(2)–B(1)–N(1)	−10.5(2)	C(6)–N(2)–B(1)–C(24)	−129.18(16)
C(6)–N(2)–B(1)–C(25)	104.52(18)	C(9)–N(2)–B(1)–N(1)	177.27(14)
C(9)–N(2)–B(1)–C(24)	58.6(2)	C(9)–N(2)–B(1)–C(25)	−67.7(2)
B(1)–N(1)–C(1)–C(2)	−176.02(14)	B(1)–N(1)–C(1)–C(10)	4.3(3)
C(4)–N(1)–C(1)–C(2)	−0.84(18)	C(4)–N(1)–C(1)–C(10)	179.47(16)
N(1)–C(1)–C(2)–C(3)	0.65(19)	N(1)–C(1)–C(2)–C(12)	179.13(15)
C(10)–C(1)–C(2)–C(3)	−179.66(16)	C(10)–C(1)–C(2)–C(12)	−1.2(3)
C(1)–C(2)–C(3)–C(4)	−0.18(18)	C(1)–C(2)–C(3)–C(13)	−179.88(14)
C(12)–C(2)–C(3)–C(4)	−178.56(15)	C(12)–C(2)–C(3)–C(13)	1.7(3)
B(1)–N(1)–C(4)–C(3)	175.98(14)	B(1)–N(1)–C(4)–C(5)	−2.9(2)
C(1)–N(1)–C(4)–C(3)	0.72(17)	C(1)–N(1)–C(4)–C(5)	−178.18(14)
C(2)–C(3)–C(4)–N(1)	−0.32(17)	C(2)–C(3)–C(4)–C(5)	178.41(16)
C(13)–C(3)–C(4)–N(1)	179.36(14)	C(13)–C(3)–C(4)–C(5)	−1.9(3)
N(1)–C(4)–C(5)–C(6)	−1.5(2)	N(1)–C(4)–C(5)–C(18)	177.12(13)
C(3)–C(4)–C(5)–C(6)	179.85(15)	C(3)–C(4)–C(5)–C(18)	−1.5(2)
C(4)–C(5)–C(6)–N(2)	−0.9(2)	C(4)–C(5)–C(6)–C(7)	−179.95(15)
C(18)–C(5)–C(6)–N(2)	−179.56(13)	C(18)–C(5)–C(6)–C(7)	1.4(2)
B(1)–N(2)–C(6)–C(5)	8.0(2)	B(1)–N(2)–C(6)–C(7)	−172.79(14)
C(9)–N(2)–C(6)–C(5)	−178.51(14)	C(9)–N(2)–C(6)–C(7)	0.74(17)
N(2)–C(6)–C(7)–C(8)	−0.50(17)	N(2)–C(6)–C(7)–C(14)	178.18(16)
C(5)–C(6)–C(7)–C(8)	178.64(16)	C(5)–C(6)–C(7)–C(14)	−2.7(3)
C(6)–C(7)–C(8)–C(9)	0.07(18)	C(6)–C(7)–C(8)–C(15)	−178.52(16)
C(14)–C(7)–C(8)–C(9)	−178.66(16)	C(14)–C(7)–C(8)–C(15)	2.7(3)
B(1)–N(2)–C(9)–C(8)	172.72(14)	B(1)–N(2)–C(9)–C(17)	−8.6(3)
C(6)–N(2)–C(9)–C(8)	−0.70(18)	C(6)–N(2)–C(9)–C(17)	177.93(16)
C(7)–C(8)–C(9)–N(2)	0.40(19)	C(7)–C(8)–C(9)–C(17)	−178.22(16)
C(15)–C(8)–C(9)–N(2)	179.03(15)	C(15)–C(8)–C(9)–C(17)	0.4(3)
C(1)–C(2)–C(12)–C(11)	−83.5(2)	C(3)–C(2)–C(12)–C(11)	94.6(2)
C(7)–C(8)–C(15)–C(16)	91.5(2)	C(9)–C(8)–C(15)–C(16)	−86.8(2)
C(4)–C(5)–C(18)–C(19)	93.30(19)	C(4)–C(5)–C(18)–C(23)	−85.97(19)
C(6)–C(5)–C(18)–C(19)	−88.0(2)	C(6)–C(5)–C(18)–C(23)	92.74(18)
C(5)–C(18)–C(19)–C(20)	−178.83(16)	C(23)–C(18)–C(19)–C(20)	0.4(3)
C(18)–C(19)–C(20)–C(21)	−0.3(3)	C(19)–C(20)–C(21)–P(1)	177.28(14)
C(19)–C(20)–C(21)–C(22)	0.1(3)	P(1)–C(21)–C(22)–C(23)	−177.04(13)
C(20)–C(21)–C(22)–C(23)	0.1(3)	C(21)–C(22)–C(23)–C(18)	0.0(3)
C(5)–C(18)–C(23)–C(22)	179.02(16)	C(19)–C(18)–C(23)–C(22)	−0.3(2)

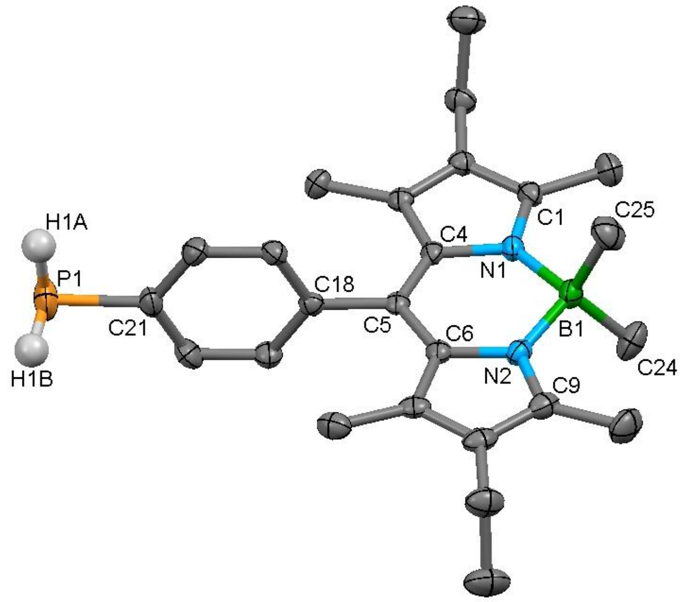

Figure S4. View of the molecular structure of **4b** with 50% probability displacement ellipsoids. Hydrogen atoms bound to carbon have been omitted for clarity.

3.6 Crystallographic data for **6b**

Table 24. Crystal data and structure refinement for **6b**.

Identification code	6b	
Chemical formula (moiety)	$C_{55}H_{60}BClN_2O_2P_3Re$	
Chemical formula (total)	$C_{55}H_{60}BClN_2O_2P_3Re$	
Formula weight	1106.42	
Temperature	150(2) K	
Radiation, wavelength	MoKα, 0.71073 Å	
Crystal system, space group	monoclinic, $P2_1$	
Unit cell parameters	a = 8.1749(4) Å	α = 90°
	b = 10.0931(4) Å	β = 91.786(4)°
	c = 33.0892(12) Å	γ = 90°
Cell volume	2728.9(2) $Å^3$	
Z	2	
Calculated density	1.347 g/cm^3	
Absorption coefficient μ	2.403 mm^{-1}	
F(000)	1124	
Crystal colour and size	red, 0.42 × 0.20 × 0.10 mm^3	
Reflections for cell refinement	6612 (θ range 2.7 to 28.5°)	
Data collection method	Oxford Diffraction Gemini A Ultra diffractometer	
	ω scans	
θ range for data collection	2.7 to 28.6°	
Index ranges	h −10 to 10, k −13 to 13, l −43 to 31	
Completeness to θ = 26.0°	98.4 %	
Reflections collected	17360	
Independent reflections	10027 (R_{int} = 0.0381)	
Reflections with $F^2>2\sigma$	9293	
Absorption correction	semi-empirical from equivalents	
Min. and max. transmission	0.432 and 0.795	
Structure solution	direct methods	
Refinement method	Full-matrix least-squares on F^2	
Weighting parameters a, b	0.0321, 0.0000	
Data / restraints / parameters	10027 / 23 / 597	
Final R indices [$F^2>2\sigma$]	R1 = 0.0348, wR2 = 0.0732	
R indices (all data)	R1 = 0.0392, wR2 = 0.0749	
Goodness-of-fit on F^2	1.013	
Absolute structure parameter	−0.014(5)	
Extinction coefficient	0.00021(18)	
Largest and mean shift/su	0.001 and 0.000	
Largest diff. peak and hole	1.50 and −1.19 e $Å^{-3}$	

Table 25. Atomic coordinates and equivalent isotropic displacement parameters (Å^2) for **6b**. U_{eq} is defined as one third of the trace of the orthogonalized U^{ij} tensor.

	x	y	z	U_{eq}
Re	0.538804(18)	0.68928(2)	0.370363(5)	0.01756(6)
P(1)	0.64056(15)	0.91033(14)	0.37704(4)	0.0201(3)
P(2)	0.33140(15)	0.80579(14)	0.33129(4)	0.0193(3)
P(3)	0.33169(15)	0.52261(14)	0.36616(4)	0.0200(3)
Cl	0.37701(16)	0.76065(14)	0.43027(4)	0.0279(3)
N(1)	0.3144(6)	0.7740(6)	0.07972(15)	0.0452(14)
N(2)	0.0460(6)	0.6673(7)	0.09605(13)	0.0418(17)
B	0.1651(8)	0.6968(16)	0.06001(19)	0.053(2)
C(1)	0.4427(9)	0.8244(9)	0.0611(2)	0.054(2)
O(1)	0.7493(4)	0.5897(4)	0.30293(12)	0.0335(9)
C(2)	0.5563(8)	0.8805(8)	0.0890(2)	0.0503(19)
O(2)	0.7902(5)	0.5472(5)	0.42652(13)	0.0429(11)
C(3)	0.4949(8)	0.8576(8)	0.1269(2)	0.0466(18)
C(4)	0.3445(8)	0.7928(8)	0.12125(17)	0.0418(16)
C(5)	0.2277(7)	0.7516(7)	0.14900(17)	0.0366(15)
C(6)	0.0818(6)	0.6927(11)	0.13703(14)	0.0385(12)
C(7)	–0.0524(7)	0.6475(7)	0.16018(19)	0.0422(18)
C(8)	–0.1655(8)	0.5971(8)	0.1328(2)	0.0521(19)
C(9)	–0.1034(9)	0.6096(9)	0.0945(2)	0.059(2)
C(10)	0.4580(10)	0.8280(11)	0.0156(2)	0.078(3)
C(11)	0.6758(13)	1.1014(11)	0.0752(3)	0.101(4)
C(12)	0.7063(10)	0.9514(10)	0.0782(3)	0.074(3)
C(13)	0.5805(9)	0.9038(9)	0.1657(2)	0.056(2)
C(14)	–0.0720(7)	0.6514(7)	0.20482(19)	0.048(2)
C(15)	–0.3180(16)	0.3934(16)	0.1566(9)	0.129(7)
C(15A)	–0.322(8)	0.440(19)	0.178(5)	0.11(3)
C(16)	–0.3284(9)	0.5341(10)	0.1426(3)	0.075(3)
C(17)	–0.1865(10)	0.5630(11)	0.0553(2)	0.084(3)
C(18)	0.2639(7)	0.7709(7)	0.19285(17)	0.0329(14)
C(19)	0.2135(8)	0.8845(7)	0.21279(18)	0.0423(17)
C(20)	0.2377(7)	0.8970(6)	0.25379(17)	0.0336(14)
C(21)	0.3203(6)	0.7991(6)	0.27611(15)	0.0230(11)
C(22)	0.3767(5)	0.6867(9)	0.25603(12)	0.0239(9)
C(23)	0.3469(6)	0.6744(9)	0.21481(14)	0.0280(14)
C(24)	0.6692(6)	0.6289(5)	0.32737(17)	0.0230(12)
C(25)	0.6965(6)	0.6011(5)	0.40636(17)	0.0237(12)
C(26)	0.3275(6)	0.4224(6)	0.32054(16)	0.0227(12)
C(27)	0.2008(7)	0.4265(6)	0.29118(17)	0.0303(13)
C(28)	0.2111(9)	0.3532(7)	0.25593(18)	0.0430(17)
C(29)	0.3443(9)	0.2742(7)	0.24938(19)	0.0478(18)
C(30)	0.4689(9)	0.2683(6)	0.2776(2)	0.0455(17)
C(31)	0.4605(7)	0.3456(6)	0.31316(17)	0.0314(13)
C(32)	0.3061(6)	0.3992(5)	0.40499(14)	0.0183(11)
C(33)	0.1881(7)	0.2998(6)	0.39987(17)	0.0324(14)
C(34)	0.1672(6)	0.2040(10)	0.43000(15)	0.0340(15)
C(35)	0.2647(6)	0.2089(8)	0.46451(16)	0.0290(15)
C(36)	0.3808(6)	0.3046(6)	0.46991(16)	0.0279(12)
C(37)	0.4019(6)	0.3990(6)	0.44043(16)	0.0262(12)
C(38)	0.7529(6)	0.9873(6)	0.33572(16)	0.0247(12)
C(39)	0.7410(6)	0.9343(6)	0.29696(16)	0.0267(12)
C(40)	0.8101(7)	0.9992(6)	0.26486(18)	0.0322(13)
C(41)	0.8936(8)	1.1148(7)	0.2710(2)	0.0464(18)
C(42)	0.9080(8)	1.1677(10)	0.30949(18)	0.055(3)
C(43)	0.8371(7)	1.1038(7)	0.34174(18)	0.0373(15)
C(44)	0.7546(6)	0.9576(5)	0.42308(15)	0.0207(11)

C(45)	0.7076(6)	1.0617(6)	0.44720(17)	0.0316(13)
C(46)	0.7912(7)	1.0870(7)	0.48353(18)	0.0387(15)
C(47)	0.9249(7)	1.0112(6)	0.49503(16)	0.0324(14)
C(48)	0.9743(7)	0.9100(6)	0.47041(17)	0.0313(13)
C(49)	0.8893(6)	0.8832(5)	0.43492(15)	0.0221(11)
C(50)	0.4583(6)	1.0189(6)	0.37707(16)	0.0242(12)
C(51)	0.3417(7)	0.9841(6)	0.34160(19)	0.0297(14)
C(52)	0.1276(7)	0.7441(6)	0.34501(19)	0.0331(15)
C(53)	0.1320(6)	0.6092(6)	0.36593(18)	0.0281(14)
C(54)	0.0729(10)	0.7903(11)	0.0262(2)	0.077(3)
C(55)	0.2320(10)	0.5582(9)	0.0428(2)	0.068(2)

Table 26. Bond lengths [Å] and angles [°] for **6b**.

Re–P(1)	2.3890(14)	Re–P(2)	2.4068(13)
Re–P(3)	2.3877(13)	Re–Cl	2.5220(13)
Re–C(24)	1.904(6)	Re–C(25)	1.943(5)
P(1)–C(38)	1.842(6)	P(1)–C(44)	1.825(5)
P(1)–C(50)	1.850(5)	P(2)–C(21)	1.827(5)
P(2)–C(51)	1.833(6)	P(2)–C(52)	1.849(6)
P(3)–C(26)	1.817(6)	P(3)–C(32)	1.807(5)
P(3)–C(53)	1.851(6)	N(1)–B	1.572(11)
N(1)–C(1)	1.334(9)	N(1)–C(4)	1.401(7)
N(2)–B	1.591(9)	N(2)–C(6)	1.402(6)
N(2)–C(9)	1.353(9)	B–C(54)	1.630(13)
B–C(55)	1.613(15)	C(1)–C(2)	1.407(10)
C(1)–C(10)	1.516(9)	O(1)–C(24)	1.128(6)
C(2)–C(3)	1.386(9)	C(2)–C(12)	1.474(10)
O(2)–C(25)	1.138(6)	C(3)–C(4)	1.401(9)
C(3)–C(13)	1.514(9)	C(4)–C(5)	1.408(9)
C(5)–C(6)	1.379(9)	C(5)–C(18)	1.485(8)
C(6)–C(7)	1.432(9)	C(7)–C(8)	1.371(9)
C(7)–C(14)	1.491(9)	C(8)–C(9)	1.388(10)
C(8)–C(16)	1.519(11)	C(9)–C(17)	1.518(10)
C(10)–H(10A)	0.980	C(10)–H(10B)	0.980
C(10)–H(10C)	0.980	C(11)–H(11A)	0.980
C(11)–H(11B)	0.980	C(11)–H(11C)	0.980
C(11)–C(12)	1.538(14)	C(12)–H(12A)	0.990
C(12)–H(12B)	0.990	C(13)–H(13A)	0.980
C(13)–H(13B)	0.980	C(13)–H(13C)	0.980
C(14)–H(14A)	0.980	C(14)–H(14B)	0.980
C(14)–H(14C)	0.980	C(15)–H(15A)	0.980
C(15)–H(15B)	0.980	C(15)–H(15C)	0.980
C(15)–C(16)	1.497(16)	C(15A)–H(15D)	0.980
C(15A)–H(15E)	0.980	C(15A)–H(15F)	0.980
C(15A)–C(16)	1.50(2)	C(16)–H(16A)	0.990
C(16)–H(16B)	0.990	C(16)–H(16C)	0.990
C(16)–H(16D)	0.990	C(17)–H(17A)	0.980
C(17)–H(17B)	0.980	C(17)–H(17C)	0.980
C(18)–C(19)	1.392(9)	C(18)–C(23)	1.380(9)
C(19)–H(19)	0.950	C(19)–C(20)	1.371(8)
C(20)–H(20)	0.950	C(20)–C(21)	1.396(8)
C(21)–C(22)	1.400(9)	C(22)–H(22)	0.950
C(22)–C(23)	1.384(6)	C(23)–H(23)	0.950
C(26)–C(27)	1.398(7)	C(26)–C(31)	1.364(8)
C(27)–H(27)	0.950	C(27)–C(28)	1.386(8)
C(28)–H(28)	0.950	C(28)–C(29)	1.373(10)
C(29)–H(29)	0.950	C(29)–C(30)	1.362(9)
C(30)–H(30)	0.950	C(30)–C(31)	1.414(9)
C(31)–H(31)	0.950	C(32)–C(33)	1.398(8)
C(32)–C(37)	1.390(7)	C(33)–H(33)	0.950
C(33)–C(34)	1.403(10)	C(34)–H(34)	0.950
C(34)–C(35)	1.373(7)	C(35)–H(35)	0.950
C(35)–C(36)	1.362(9)	C(36)–H(36)	0.950
C(36)–C(37)	1.378(8)	C(37)–H(37)	0.950
C(38)–C(39)	1.390(8)	C(38)–C(43)	1.375(8)
C(39)–H(39)	0.950	C(39)–C(40)	1.384(8)
C(40)–H(40)	0.950	C(40)–C(41)	1.363(9)
C(41)–H(41)	0.950	C(41)–C(42)	1.383(9)
C(42)–H(42)	0.950	C(42)–C(43)	1.389(9)
C(43)–H(43)	0.950	C(44)–C(45)	1.381(8)
C(44)–C(49)	1.379(7)	C(45)–H(45)	0.950

C(45)–C(46)	1.388(8)	C(46)–H(46)	0.950
C(46)–C(47)	1.378(9)	C(47)–H(47)	0.950
C(47)–C(48)	1.375(9)	C(48)–H(48)	0.950
C(48)–C(49)	1.372(7)	C(49)–H(49)	0.950
C(50)–H(50A)	0.990	C(50)–H(50B)	0.990
C(50)–C(51)	1.529(7)	C(51)–H(51A)	0.990
C(51)–H(51B)	0.990	C(52)–H(52A)	0.990
C(52)–H(52B)	0.990	C(52)–C(53)	1.527(8)
C(53)–H(53A)	0.990	C(53)–H(53B)	0.990
C(54)–H(54A)	0.980	C(54)–H(54B)	0.980
C(54)–H(54C)	0.980	C(55)–H(55A)	0.980
C(55)–H(55B)	0.980	C(55)–H(55C)	0.980

P(1)–Re–P(2)	80.26(5)	P(1)–Re–P(3)	155.18(5)
P(1)–Re–Cl	81.39(5)	P(1)–Re–C(24)	99.58(16)
P(1)–Re–C(25)	98.54(16)	P(2)–Re–P(3)	80.01(5)
P(2)–Re–Cl	84.53(4)	P(2)–Re–C(24)	99.00(15)
P(2)–Re–C(25)	174.70(16)	P(3)–Re–Cl	81.88(5)
P(3)–Re–C(24)	98.25(15)	P(3)–Re–C(25)	99.72(16)
Cl–Re–C(24)	176.44(16)	Cl–Re–C(25)	90.19(16)
C(24)–Re–C(25)	86.3(2)	Re–P(1)–C(38)	120.34(19)
Re–P(1)–C(44)	119.25(18)	Re–P(1)–C(50)	105.98(18)
C(38)–P(1)–C(44)	104.8(2)	C(38)–P(1)–C(50)	99.8(3)
C(44)–P(1)–C(50)	103.6(2)	Re–P(2)–C(21)	122.08(18)
Re–P(2)–C(51)	110.61(18)	Re–P(2)–C(52)	109.18(19)
C(21)–P(2)–C(51)	102.9(3)	C(21)–P(2)–C(52)	102.5(3)
C(51)–P(2)–C(52)	108.8(3)	Re–P(3)–C(26)	115.84(18)
Re–P(3)–C(32)	122.82(17)	Re–P(3)–C(53)	106.9(2)
C(26)–P(3)–C(32)	102.0(3)	C(26)–P(3)–C(53)	105.4(3)
C(32)–P(3)–C(53)	101.9(2)	B–N(1)–C(1)	127.5(5)
B–N(1)–C(4)	125.8(5)	C(1)–N(1)–C(4)	106.5(6)
B–N(2)–C(6)	125.2(5)	B–N(2)–C(9)	128.7(5)
C(6)–N(2)–C(9)	106.1(5)	N(1)–B–N(2)	105.5(5)
N(1)–B–C(54)	109.5(10)	N(1)–B–C(55)	108.0(6)
N(2)–B–C(54)	110.0(6)	N(2)–B–C(55)	109.0(10)
C(54)–B–C(55)	114.5(6)	N(1)–C(1)–C(2)	111.3(6)
N(1)–C(1)–C(10)	123.9(7)	C(2)–C(1)–C(10)	124.6(7)
C(1)–C(2)–C(3)	106.1(6)	C(1)–C(2)–C(12)	125.1(7)
C(3)–C(2)–C(12)	128.8(7)	C(2)–C(3)–C(4)	107.3(6)
C(2)–C(3)–C(13)	123.0(7)	C(4)–C(3)–C(13)	129.6(6)
N(1)–C(4)–C(3)	108.8(6)	N(1)–C(4)–C(5)	119.8(6)
C(3)–C(4)–C(5)	131.4(6)	C(4)–C(5)–C(6)	122.5(5)
C(4)–C(5)–C(18)	118.7(5)	C(6)–C(5)–C(18)	118.8(5)
N(2)–C(6)–C(5)	120.6(5)	N(2)–C(6)–C(7)	108.6(6)
C(5)–C(6)–C(7)	130.8(5)	C(6)–C(7)–C(8)	106.1(6)
C(6)–C(7)–C(14)	129.0(6)	C(8)–C(7)–C(14)	124.9(6)
C(7)–C(8)–C(9)	108.2(6)	C(7)–C(8)–C(16)	126.4(7)
C(9)–C(8)–C(16)	125.4(7)	N(2)–C(9)–C(8)	111.0(6)
N(2)–C(9)–C(17)	123.1(7)	C(8)–C(9)–C(17)	125.9(7)
C(1)–C(10)–H(10A)	109.5	C(1)–C(10)–H(10B)	109.5
C(1)–C(10)–H(10C)	109.5	H(10A)–C(10)–H(10B)	109.5
H(10A)–C(10)–H(10C)	109.5	H(10B)–C(10)–H(10C)	109.5
H(11A)–C(11)–H(11B)	109.5	H(11A)–C(11)–H(11C)	109.5
H(11A)–C(11)–C(12)	109.5	H(11B)–C(11)–H(11C)	109.5
H(11B)–C(11)–C(12)	109.5	H(11C)–C(11)–C(12)	109.5
C(2)–C(12)–C(11)	111.1(8)	C(2)–C(12)–H(12A)	109.4
C(2)–C(12)–H(12B)	109.4	C(11)–C(12)–H(12A)	109.4
C(11)–C(12)–H(12B)	109.4	H(12A)–C(12)–H(12B)	108.0
C(3)–C(13)–H(13A)	109.5	C(3)–C(13)–H(13B)	109.5
C(3)–C(13)–H(13C)	109.5	H(13A)–C(13)–H(13B)	109.5

H(13A)–C(13)–H(13C)	109.5	H(13B)–C(13)–H(13C)	109.5
C(7)–C(14)–H(14A)	109.5	C(7)–C(14)–H(14B)	109.5
C(7)–C(14)–H(14C)	109.5	H(14A)–C(14)–H(14B)	109.5
H(14A)–C(14)–H(14C)	109.5	H(14B)–C(14)–H(14C)	109.5
H(15A)–C(15)–H(15B)	109.5	H(15A)–C(15)–H(15C)	109.5
H(15A)–C(15)–C(16)	109.5	H(15B)–C(15)–H(15C)	109.5
H(15B)–C(15)–C(16)	109.5	H(15C)–C(15)–C(16)	109.5
H(15D)–C(15A)–H(15E)	109.5	H(15D)–C(15A)–H(15F)	109.5
H(15D)–C(15A)–C(16)	109.5	H(15E)–C(15A)–H(15F)	109.5
H(15E)–C(15A)–C(16)	109.5	H(15F)–C(15A)–C(16)	109.5
C(8)–C(16)–C(15)	114.9(8)	C(8)–C(16)–C(15A)	114.9(19)
C(8)–C(16)–H(16A)	108.5	C(8)–C(16)–H(16B)	108.5
C(8)–C(16)–H(16C)	108.5	C(8)–C(16)–H(16D)	108.5
C(15)–C(16)–H(16A)	108.5	C(15)–C(16)–H(16B)	108.5
C(15A)–C(16)–H(16C)	108.5	C(15A)–C(16)–H(16D)	108.5
H(16A)–C(16)–H(16B)	107.5	H(16C)–C(16)–H(16D)	107.5
C(9)–C(17)–H(17A)	109.5	C(9)–C(17)–H(17B)	109.5
C(9)–C(17)–H(17C)	109.5	H(17A)–C(17)–H(17B)	109.5
H(17A)–C(17)–H(17C)	109.5	H(17B)–C(17)–H(17C)	109.5
C(5)–C(18)–C(19)	121.2(6)	C(5)–C(18)–C(23)	120.1(6)
C(19)–C(18)–C(23)	118.7(6)	C(18)–C(19)–H(19)	119.7
C(18)–C(19)–C(20)	120.6(6)	H(19)–C(19)–C(20)	119.7
C(19)–C(20)–H(20)	119.6	C(19)–C(20)–C(21)	120.7(6)
H(20)–C(20)–C(21)	119.6	P(2)–C(21)–C(20)	120.7(4)
P(2)–C(21)–C(22)	119.8(4)	C(20)–C(21)–C(22)	119.0(5)
C(21)–C(22)–H(22)	120.3	C(21)–C(22)–C(23)	119.3(7)
H(22)–C(22)–C(23)	120.3	C(18)–C(23)–C(22)	121.6(7)
C(18)–C(23)–H(23)	119.2	C(22)–C(23)–H(23)	119.2
Re–C(24)–O(1)	177.3(5)	Re–C(25)–O(2)	178.0(5)
P(3)–C(26)–C(27)	123.7(4)	P(3)–C(26)–C(31)	118.1(4)
C(27)–C(26)–C(31)	118.0(5)	C(26)–C(27)–H(27)	119.8
C(26)–C(27)–C(28)	120.4(6)	H(27)–C(27)–C(28)	119.8
C(27)–C(28)–H(28)	119.6	C(27)–C(28)–C(29)	120.8(6)
H(28)–C(28)–C(29)	119.6	C(28)–C(29)–H(29)	120.1
C(28)–C(29)–C(30)	119.8(6)	H(29)–C(29)–C(30)	120.1
C(29)–C(30)–H(30)	120.3	C(29)–C(30)–C(31)	119.5(7)
H(30)–C(30)–C(31)	120.3	C(26)–C(31)–C(30)	121.5(6)
C(26)–C(31)–H(31)	119.3	C(30)–C(31)–H(31)	119.3
P(3)–C(32)–C(33)	120.2(4)	P(3)–C(32)–C(37)	121.7(4)
C(33)–C(32)–C(37)	118.0(5)	C(32)–C(33)–H(33)	119.8
C(32)–C(33)–C(34)	120.5(5)	H(33)–C(33)–C(34)	119.8
C(33)–C(34)–H(34)	120.5	C(33)–C(34)–C(35)	119.0(7)
H(34)–C(34)–C(35)	120.5	C(34)–C(35)–H(35)	119.4
C(34)–C(35)–C(36)	121.3(6)	H(35)–C(35)–C(36)	119.4
C(35)–C(36)–H(36)	120.0	C(35)–C(36)–C(37)	120.0(5)
H(36)–C(36)–C(37)	120.0	C(32)–C(37)–C(36)	121.2(5)
C(32)–C(37)–H(37)	119.4	C(36)–C(37)–H(37)	119.4
P(1)–C(38)–C(39)	120.0(4)	P(1)–C(38)–C(43)	120.8(4)
C(39)–C(38)–C(43)	118.9(5)	C(38)–C(39)–H(39)	119.8
C(38)–C(39)–C(40)	120.5(5)	H(39)–C(39)–C(40)	119.8
C(39)–C(40)–H(40)	119.8	C(39)–C(40)–C(41)	120.4(6)
H(40)–C(40)–C(41)	119.8	C(40)–C(41)–H(41)	120.2
C(40)–C(41)–C(42)	119.7(6)	H(41)–C(41)–C(42)	120.2
C(41)–C(42)–H(42)	119.9	C(41)–C(42)–C(43)	120.2(7)
H(42)–C(42)–C(43)	119.9	C(38)–C(43)–C(42)	120.3(6)
C(38)–C(43)–H(43)	119.8	C(42)–C(43)–H(43)	119.8
P(1)–C(44)–C(45)	122.5(4)	P(1)–C(44)–C(49)	118.5(4)
C(45)–C(44)–C(49)	119.0(5)	C(44)–C(45)–H(45)	119.9
C(44)–C(45)–C(46)	120.1(6)	H(45)–C(45)–C(46)	119.9
C(45)–C(46)–H(46)	119.9	C(45)–C(46)–C(47)	120.1(6)

H(46)–C(46)–C(47)	119.9	C(46)–C(47)–H(47)	120.2
C(46)–C(47)–C(48)	119.6(5)	H(47)–C(47)–C(48)	120.2
C(47)–C(48)–H(48)	119.9	C(47)–C(48)–C(49)	120.2(5)
H(48)–C(48)–C(49)	119.9	C(44)–C(49)–C(48)	120.9(5)
C(44)–C(49)–H(49)	119.5	C(48)–C(49)–H(49)	119.5
P(1)–C(50)–H(50A)	109.6	P(1)–C(50)–H(50B)	109.6
P(1)–C(50)–C(51)	110.2(4)	H(50A)–C(50)–H(50B)	108.1
H(50A)–C(50)–C(51)	109.6	H(50B)–C(50)–C(51)	109.6
P(2)–C(51)–C(50)	113.1(4)	P(2)–C(51)–H(51A)	109.0
P(2)–C(51)–H(51B)	109.0	C(50)–C(51)–H(51A)	109.0
C(50)–C(51)–H(51B)	109.0	H(51A)–C(51)–H(51B)	107.8
P(2)–C(52)–H(52A)	108.8	P(2)–C(52)–H(52B)	108.8
P(2)–C(52)–C(53)	113.8(4)	H(52A)–C(52)–H(52B)	107.7
H(52A)–C(52)–C(53)	108.8	H(52B)–C(52)–C(53)	108.8
P(3)–C(53)–C(52)	115.5(4)	P(3)–C(53)–H(53A)	108.4
P(3)–C(53)–H(53B)	108.4	C(52)–C(53)–H(53A)	108.4
C(52)–C(53)–H(53B)	108.4	H(53A)–C(53)–H(53B)	107.5
B–C(54)–H(54A)	109.5	B–C(54)–H(54B)	109.5
B–C(54)–H(54C)	109.5	H(54A)–C(54)–H(54B)	109.5
H(54A)–C(54)–H(54C)	109.5	H(54B)–C(54)–H(54C)	109.5
B–C(55)–H(55A)	109.5	B–C(55)–H(55B)	109.5
B–C(55)–H(55C)	109.5	H(55A)–C(55)–H(55B)	109.5
H(55A)–C(55)–H(55C)	109.5	H(55B)–C(55)–H(55C)	109.5

Table 27. Anisotropic displacement parameters (Å^2) for **6b**. The anisotropic displacement factor exponent takes the form: $-2\pi^2[h^2a^{*2}U^{11} + ...+ 2hka^*b^*U^{12}]$

	U^{11}	U^{22}	U^{33}	U^{23}	U^{13}	U^{12}
Re	0.01722(9)	0.01801(9)	0.01747(9)	0.00051(13)	0.00076(6)	–0.00151(12)
P(1)	0.0202(6)	0.0199(7)	0.0203(7)	–0.0016(6)	0.0025(5)	–0.0013(6)
P(2)	0.0197(6)	0.0215(7)	0.0167(6)	0.0016(6)	0.0009(5)	0.0009(5)
P(3)	0.0208(6)	0.0201(7)	0.0192(7)	0.0014(6)	0.0008(5)	–0.0022(6)
Cl	0.0309(7)	0.0323(8)	0.0207(6)	–0.0001(6)	0.0038(5)	–0.0028(6)
N(1)	0.042(3)	0.070(4)	0.023(3)	0.001(3)	0.002(2)	0.000(3)
N(2)	0.041(3)	0.052(5)	0.032(2)	–0.012(3)	–0.0017(19)	–0.003(3)
B	0.051(4)	0.082(6)	0.026(3)	–0.016(6)	0.002(3)	0.015(7)
C(1)	0.057(4)	0.076(6)	0.030(4)	0.008(4)	0.010(3)	0.005(4)
O(1)	0.030(2)	0.036(3)	0.035(2)	–0.008(2)	0.0078(18)	–0.0019(19)
C(2)	0.050(4)	0.069(6)	0.033(4)	0.005(4)	0.012(3)	–0.003(4)
O(2)	0.026(2)	0.048(3)	0.054(3)	0.019(2)	–0.012(2)	0.000(2)
C(3)	0.046(4)	0.057(5)	0.036(4)	0.005(3)	–0.002(3)	0.000(3)
C(4)	0.046(4)	0.060(5)	0.020(3)	0.007(3)	0.001(3)	0.004(3)
C(5)	0.043(3)	0.048(4)	0.019(3)	0.001(3)	–0.001(3)	0.006(3)
C(6)	0.044(3)	0.047(3)	0.024(2)	–0.003(5)	0.000(2)	0.004(5)
C(7)	0.036(3)	0.053(5)	0.038(3)	–0.010(3)	0.005(3)	0.006(3)
C(8)	0.043(4)	0.065(5)	0.049(4)	–0.011(4)	0.011(3)	0.002(4)
C(9)	0.046(4)	0.082(6)	0.049(5)	–0.023(4)	–0.007(3)	–0.004(4)
C(10)	0.080(6)	0.123(9)	0.032(4)	0.000(5)	0.016(4)	–0.009(6)
C(11)	0.113(8)	0.087(8)	0.104(9)	0.020(7)	0.024(7)	–0.032(7)
C(12)	0.069(5)	0.096(8)	0.056(5)	0.013(5)	0.014(4)	–0.025(5)
C(13)	0.058(5)	0.068(6)	0.041(4)	–0.001(4)	0.004(3)	–0.017(4)
C(14)	0.044(3)	0.059(6)	0.041(4)	0.002(3)	0.011(3)	–0.003(3)
C(15)	0.107(10)	0.091(9)	0.193(18)	–0.038(11)	0.081(10)	–0.044(8)
C(15A)	0.12(5)	0.10(5)	0.13(5)	0.00(3)	0.03(5)	0.00(5)
C(16)	0.052(5)	0.101(7)	0.073(6)	–0.030(5)	0.011(4)	–0.025(5)
C(17)	0.066(5)	0.130(10)	0.054(5)	–0.026(6)	–0.006(4)	–0.026(6)
C(18)	0.035(3)	0.043(4)	0.022(3)	0.003(3)	0.003(2)	0.002(3)
C(19)	0.055(4)	0.047(4)	0.025(3)	0.004(3)	–0.004(3)	0.017(3)
C(20)	0.046(3)	0.030(3)	0.024(3)	0.002(3)	–0.001(3)	0.015(3)
C(21)	0.025(3)	0.027(3)	0.016(2)	0.003(2)	0.001(2)	–0.005(2)
C(22)	0.025(2)	0.026(2)	0.021(2)	0.006(4)	0.0032(17)	0.000(4)
C(23)	0.031(2)	0.030(4)	0.024(2)	–0.001(3)	0.0057(19)	–0.003(3)
C(24)	0.020(3)	0.016(3)	0.033(3)	–0.002(2)	–0.007(2)	–0.004(2)
C(25)	0.022(3)	0.018(3)	0.031(3)	–0.002(2)	0.002(2)	–0.004(2)
C(26)	0.028(3)	0.020(3)	0.021(3)	0.003(2)	0.006(2)	–0.004(2)
C(27)	0.034(3)	0.025(3)	0.032(3)	0.003(3)	0.003(2)	–0.010(3)
C(28)	0.059(4)	0.043(4)	0.027(3)	0.000(3)	–0.009(3)	–0.022(4)
C(29)	0.080(5)	0.035(4)	0.028(3)	–0.003(3)	0.006(3)	–0.018(4)
C(30)	0.070(5)	0.026(4)	0.042(4)	–0.003(3)	0.024(3)	–0.005(3)
C(31)	0.042(3)	0.023(3)	0.029(3)	0.000(3)	0.006(3)	–0.011(3)
C(32)	0.021(2)	0.019(3)	0.015(2)	–0.001(2)	0.005(2)	0.003(2)
C(33)	0.036(3)	0.035(4)	0.026(3)	0.005(3)	–0.001(2)	–0.011(3)
C(34)	0.037(3)	0.035(4)	0.031(3)	0.005(4)	0.008(2)	–0.015(4)
C(35)	0.036(3)	0.024(4)	0.028(2)	0.008(3)	0.011(2)	0.006(3)
C(36)	0.030(3)	0.031(3)	0.022(3)	0.004(3)	–0.001(2)	0.004(3)
C(37)	0.028(3)	0.024(3)	0.027(3)	0.004(2)	0.004(2)	–0.003(2)
C(38)	0.021(3)	0.027(3)	0.026(3)	0.006(2)	–0.002(2)	–0.004(2)
C(39)	0.030(3)	0.026(3)	0.024(3)	0.004(2)	0.005(2)	–0.006(2)
C(40)	0.037(3)	0.032(4)	0.028(3)	0.003(3)	0.007(2)	–0.002(3)
C(41)	0.043(4)	0.062(5)	0.034(4)	0.023(3)	0.001(3)	–0.018(3)

C(42)	0.065(4)	0.060(7)	0.039(3)	0.014(4)	–0.013(3)	–0.044(5)
C(43)	0.046(4)	0.037(4)	0.028(3)	0.003(3)	–0.009(3)	–0.017(3)
C(44)	0.024(3)	0.020(3)	0.018(3)	0.001(2)	0.002(2)	–0.005(2)
C(45)	0.025(3)	0.033(3)	0.037(3)	–0.013(3)	0.005(2)	–0.006(3)
C(46)	0.037(3)	0.046(4)	0.033(3)	–0.012(3)	0.006(3)	–0.013(3)
C(47)	0.032(3)	0.045(4)	0.020(3)	–0.002(3)	–0.002(2)	–0.018(3)
C(48)	0.029(3)	0.035(3)	0.030(3)	0.009(3)	–0.004(2)	–0.007(3)
C(49)	0.019(3)	0.023(3)	0.025(3)	–0.001(2)	0.006(2)	–0.006(2)
C(50)	0.025(3)	0.018(3)	0.030(3)	–0.003(2)	–0.001(2)	0.000(2)
C(51)	0.037(3)	0.021(3)	0.030(3)	–0.002(3)	–0.005(3)	0.008(3)
C(52)	0.024(3)	0.042(4)	0.034(3)	0.009(3)	0.003(3)	0.000(2)
C(53)	0.016(3)	0.042(4)	0.027(3)	–0.001(3)	0.007(2)	–0.008(2)
C(54)	0.074(5)	0.125(9)	0.031(4)	0.004(5)	–0.009(4)	0.008(6)
C(55)	0.070(5)	0.084(7)	0.049(5)	–0.018(5)	0.016(4)	–0.008(5)

Table 28. Hydrogen coordinates and isotropic displacement parameters (Å^2) for **6b**.

	x	y	z	U
H(10A)	0.3645	0.7820	0.0027	0.117
H(10B)	0.5597	0.7839	0.0082	0.117
H(10C)	0.4598	0.9202	0.0064	0.117
H(11A)	0.7746	1.1454	0.0658	0.152
H(11B)	0.6487	1.1361	0.1018	0.152
H(11C)	0.5846	1.1184	0.0559	0.152
H(12A)	0.7443	0.9180	0.0520	0.088
H(12B)	0.7935	0.9340	0.0990	0.088
H(13A)	0.6859	0.9439	0.1594	0.083
H(13B)	0.5988	0.8280	0.1837	0.083
H(13C)	0.5122	0.9695	0.1790	0.083
H(14A)	–0.1772	0.6116	0.2114	0.072
H(14B)	–0.0689	0.7435	0.2141	0.072
H(14C)	0.0171	0.6015	0.2182	0.072
H(15A)	–0.4273	0.3622	0.1635	0.194
H(15B)	–0.2447	0.3878	0.1806	0.194
H(15C)	–0.2752	0.3379	0.1351	0.194
H(15D)	–0.4328	0.4078	0.1827	0.170
H(15E)	–0.2776	0.4850	0.2016	0.170
H(15F)	–0.2519	0.3642	0.1711	0.170
H(16A)	–0.3800	0.5876	0.1638	0.090
H(16B)	–0.4010	0.5379	0.1181	0.090
H(16C)	–0.4073	0.6055	0.1484	0.090
H(16D)	–0.3705	0.4863	0.1183	0.090
H(17A)	–0.2937	0.5252	0.0611	0.125
H(17B)	–0.1186	0.4954	0.0428	0.125
H(17C)	–0.2007	0.6383	0.0368	0.125
H(19)	0.1619	0.9541	0.1978	0.051
H(20)	0.1978	0.9733	0.2672	0.040
H(22)	0.4349	0.6195	0.2706	0.029
H(23)	0.3844	0.5977	0.2013	0.034
H(27)	0.1072	0.4799	0.2954	0.036
H(28)	0.1249	0.3577	0.2361	0.052
H(29)	0.3496	0.2237	0.2252	0.057
H(30)	0.5607	0.2128	0.2735	0.055
H(31)	0.5495	0.3439	0.3323	0.038
H(33)	0.1216	0.2972	0.3758	0.039
H(34)	0.0867	0.1367	0.4266	0.041
H(35)	0.2510	0.1443	0.4850	0.035
H(36)	0.4472	0.3064	0.4940	0.033
H(37)	0.4835	0.4651	0.4444	0.031
H(39)	0.6849	0.8529	0.2925	0.032
H(40)	0.7994	0.9631	0.2384	0.039
H(41)	0.9416	1.1587	0.2489	0.056
H(42)	0.9666	1.2479	0.3139	0.066
H(43)	0.8468	1.1409	0.3681	0.045
H(45)	0.6178	1.1161	0.4389	0.038
H(46)	0.7563	1.1567	0.5005	0.046
H(47)	0.9827	1.0288	0.5198	0.039
H(48)	1.0675	0.8584	0.4780	0.038
H(49)	0.9238	0.8125	0.4183	0.027
H(50A)	0.4012	1.0080	0.4028	0.029
H(50B)	0.4927	1.1126	0.3749	0.029
H(51A)	0.3780	1.0305	0.3171	0.036

H(51B)	0.2308	1.0166	0.3475	0.036
H(52A)	0.0768	0.8093	0.3631	0.040
H(52B)	0.0573	0.7376	0.3202	0.040
H(53A)	0.0490	0.5514	0.3525	0.034
H(53B)	0.0998	0.6214	0.3943	0.034
H(54A)	0.0337	0.8712	0.0392	0.116
H(54B)	–0.0202	0.7422	0.0140	0.116
H(54C)	0.1497	0.8138	0.0052	0.116
H(55A)	0.2761	0.5044	0.0652	0.101
H(55B)	0.3186	0.5756	0.0236	0.101
H(55C)	0.1421	0.5105	0.0290	0.101

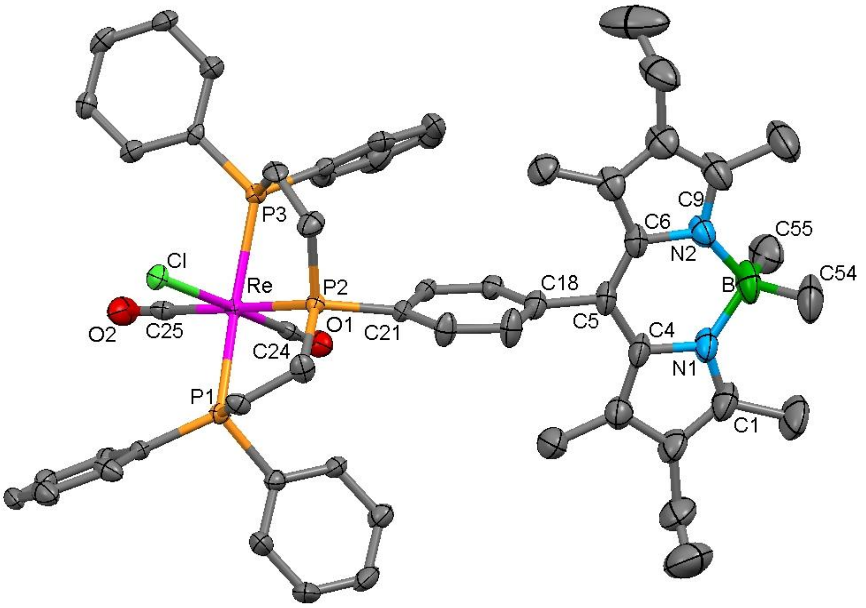

Figure S5. View of the molecular structure of **6b** with 50% probability displacement ellipsoids. Hydrogen atoms have been omitted for clarity.

4 Quantum Chemical Calculations

4.1 General considerations

All calculations were carried out using the Spartan 10 software.[4] Full geometry optimizations of the studied compounds were performed using density functional theory with a B3LYP/6-31G* basis set. A vibrational analysis was performed at the same level to characterize calculated structures as minima.

4.2 Molecular orbital surfaces

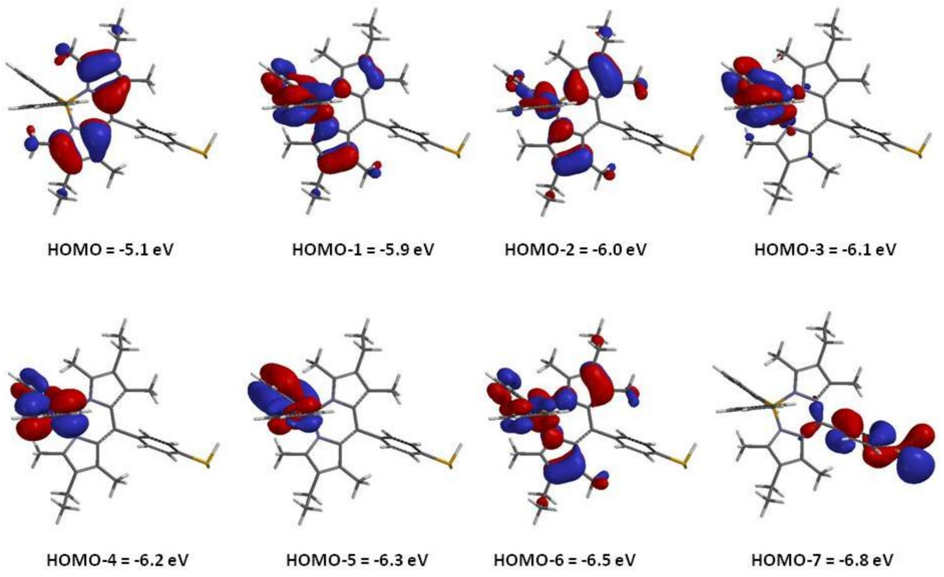

Figure S6. Calculated molecular orbital surfaces from the HOMO to HOMO-7 for **4a**.

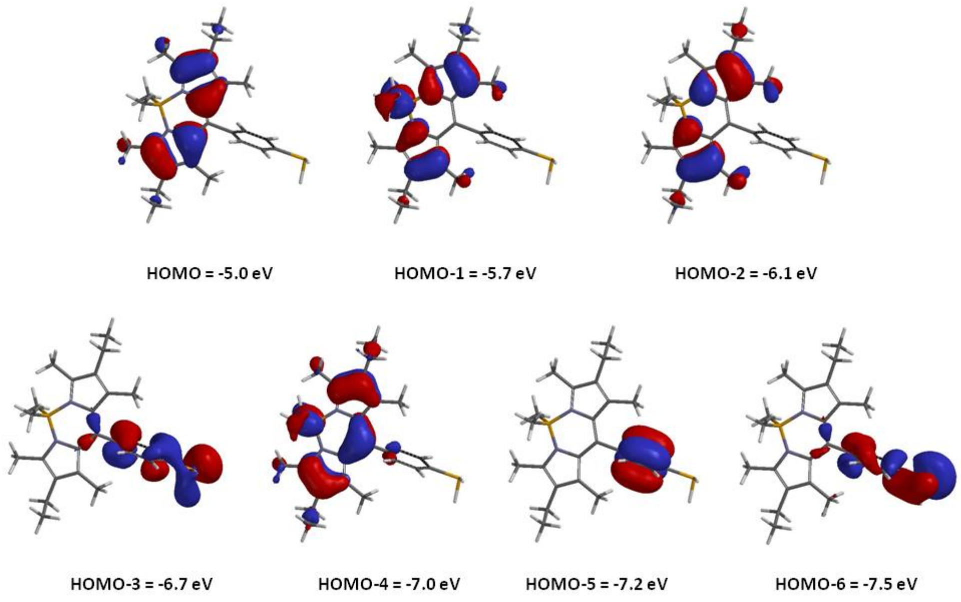

Figure S7. Calculated molecular orbital surfaces from the HOMO to HOMO-6 for **4b**.

5 Absorption and Emission Spectroscopy

5.1 General considerations

Absorption spectra were recorded with a Hitachi Model U-3310 spectrophotometer while fluorescence studies were recorded with a Hitachi F-4500 fluorescence spectrophotometer. Solvents used for spectroscopy experiments were spectrophotometric grade. Absorption and emission spectroscopy was recorded for compounds **1**-**6** in dry degassed tetrahydrofuran solution at room temperature. Fluorescence quantum yields were measured with respect to 4,4-difluoro-8-phenyl-1,3,5,7-tetramethyl -2,6-diethyl-4-bora-3a,4a-diaza-s-indacene (Φ = 0.76, λ_{abs} = 524 nm, λ_{em} = 537 nm, ε = 86,000 $M^{-1}cm^{-1}$, THF).[5] Dyes were excited at 485 nm and excitation and emission slits were both set to 5 nm.

5.2 Absorption and emission spectra

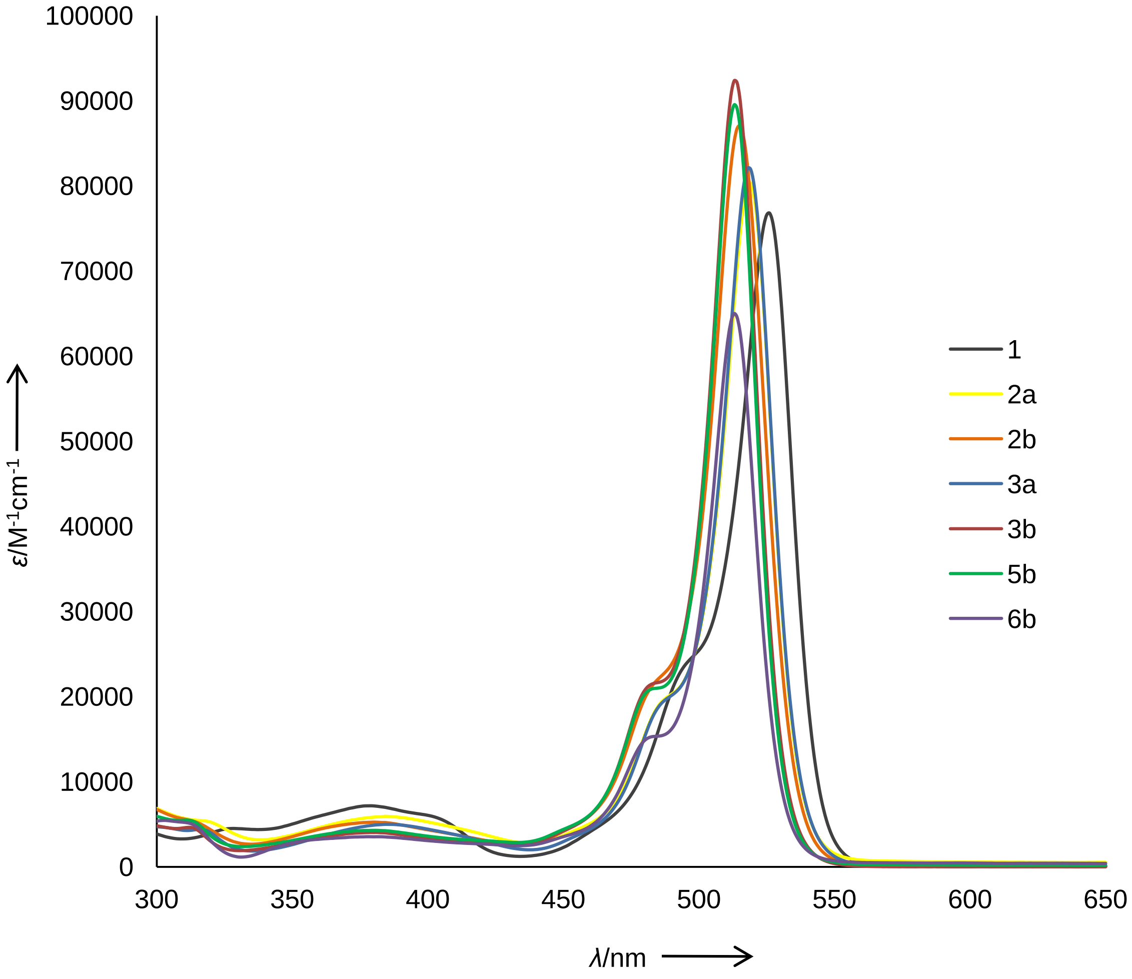

Figure S8. Absorption Spectra for **1**, **2a**/**2b**, **3a**/**3b**, **5b** and **6b**.

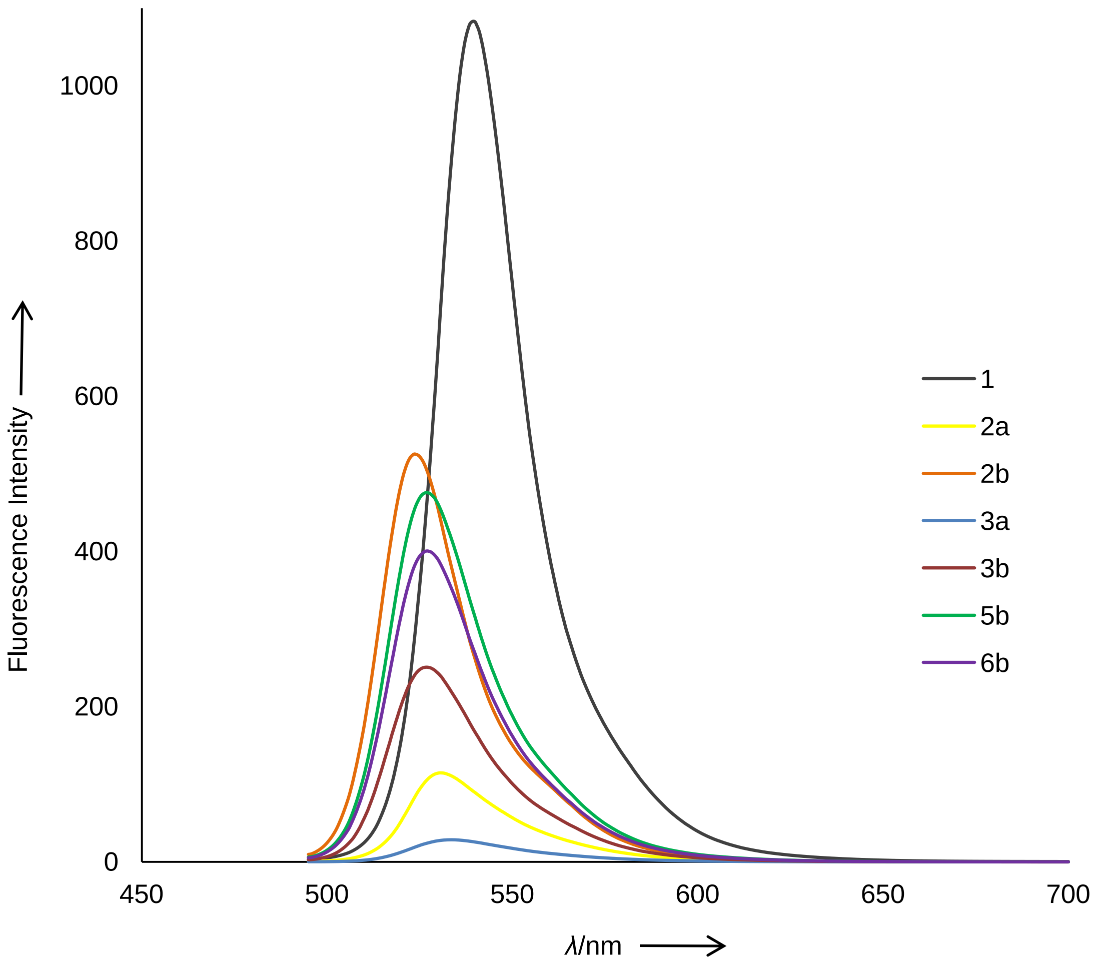

Figure S9. Fluorescence Spectra for **1**, **2a**/**2b**, **3a**/**3b**, **5b** and **6b**.

6 References

[1] A. C. Benniston, G. Copley, K. J. Elliott, R. W. Harrington, W. Clegg, *Eur. J. Org. Chem.* **2008**, *16*, 2705-2713.

[2] Y. Gabe, Y. Urano, K. Kikuchi, H. Kojima, T. Nagano, *J. Am. Chem. Soc.* **2004**, *126*, 3357-3367.

[3] J. Chatt, J. R. Dilworth, H. P. Gunz, G. J. Leigh, *J. Organomet. Chem.* **1974**, *64*, 245-254.

[4] *Spartan 10*; Wavefunction Inc., Irvine, CA, 2011.

[5] A. Coskun, E. U. Akkaya, *J. Am. Chem. Soc.* **2005**, *127*, 10464-10465.
